# Quantitative comparison of taxa and taxon concepts in the diatom genus *Fragilariopsis*: a case study on using slide scanning, multiexpert image annotation, and image analysis in taxonomy^1^

**DOI:** 10.1111/jpy.12767

**Published:** 2018-08-28

**Authors:** Bánk Beszteri, Claire Allen, Gastón O. Almandoz, Leanne Armand, María Ángeles Barcena, Hannelore Cantzler, Xavier Crosta, Oliver Esper, Richard W. Jordan, Gerhard Kauer, Christine Klaas, Michael Kloster, Amy Leventer, Jennifer Pike, Andrés S. Rigual Hernández

**Affiliations:** ^1^ Section Polar Biological Oceanography Alfred Wegener Institute Helmholtz Centre for Polar and Marine Research Am Handelshafen 12 27570 Bremerhaven Germany; ^2^ British Antarctic Survey High Cross, Madingley Road Cambridge CB3 0ET UK; ^3^ División Ficología Facultad de Ciencias Naturales y Museo Universidad Nacional de La Plata Paseo del Bosque s/n (B1900FWA) La Plata Argentina; ^4^ Research School of Earth Sciences The Australian National University Jaeger Building 4, 142 Mills Road Acton ACT 2601 Australia; ^5^ Departamento de Geología Universidad de Salamanca 37008 Salamanca Spain; ^6^ UMR‐CNRS 5805 EPOC Université de Bordeaux Allée Geoffroy Saint Hilaire 33615 Pessac Cedex France; ^7^ Section Marine Geology Alfred Wegener Institute Helmholtz Centre for Polar and Marine Research Am Handelshafen 12 27570 Bremerhaven Germany; ^8^ Department of Earth & Environmental Sciences Faculty of Science Yamagata University 1‐4‐12 Kojirakawa‐machi Yamagata 990‐8560 Japan; ^9^ Bioinformatics University of Applied Sciences Constantiaplatz 4 26723 Emden Germany; ^10^ Geology Department Colgate University Hamilton New York 13346 USA; ^11^ School of Earth and Ocean Sciences Cardiff University Main Building, Park Place Cardiff CF10 3AT UK; ^12^ Área de Paleontología Departamento de Geología Universidad de Salamanca 37008 Salamanca Spain

**Keywords:** automatic diatom identification, Bacillariophyta, high throughput microscopy, image analysis, morphometrics, SHERPA, taxonomic agreement

## Abstract

Semiautomated methods for microscopic image acquisition, image analysis, and taxonomic identification have repeatedly received attention in diatom analysis. Less well studied is the question whether and how such methods might prove useful for clarifying the delimitation of species that are difficult to separate for human taxonomists. To try to answer this question, three very similar *Fragilariopsis* species endemic to the Southern Ocean were targeted in this study: *F. obliquecostata*,* F. ritscheri*, and *F. sublinearis*. A set of 501 extended focus depth specimen images were obtained using a standardized, semiautomated microscopic procedure. Twelve diatomists independently identified these specimen images in order to reconcile taxonomic opinions and agree upon a taxonomic gold standard. Using image analyses, we then extracted morphometric features representing taxonomic characters of the target taxa. The discriminating ability of individual morphometric features was tested visually and statistically, and multivariate classification experiments were performed to test the agreement of the quantitatively defined taxa assignments with expert consensus opinion. Beyond an updated differential diagnosis of the studied taxa, our study also shows that automated imaging and image analysis procedures for diatoms are coming close to reaching a broad applicability for routine use.

**Abbreviations**
CDF: convexity defection factor
CHMDF: convex hull maximum distance factor
EFD: elliptic Fourier descriptor
LDA: linear discriminant analysis
NA: numeric aperture
PCAF: percent concave area fraction
QDA: quadratic discriminant analysis
SVM: support vector machine

Taxonomic identification of specimens is central to a broad range of scientific and applied ecological research areas. The automation of microscopic imaging and taxonomic identification has repeatedly been attempted over the last few decades, targeting individual microalgal groups like dinoflagellates (Benfield et al. 2007), coccolithophores (Beaufort and Dollfus 2004, Bollmann et al. 2005), and diatoms (du Buf and Bayer 2002b), and for phytoplankton in general (Olson and Sosik 2007, Schulze et al. 2013, Laney and Sosik 2014). Technological developments in the field of automated, in or ex situ imaging (Gorsky et al. 2010, Picheral et al. 2010, Schulz et al. 2010, Schoening et al. 2012, Biard et al. 2016) and in computer vision, notably the recent flourishing of deep convolutional neural networks (Dai et al. 2016a,b, Lee et al. 2016, Py et al. 2016, Pedraza et al. 2017), are now giving new momentum for studying a diverse range of organisms.

To date, the most substantial attempt at developing an automated imaging and image‐based taxonomic identification workflow for acid cleaned diatom frustules has been the project Automated Diatom Classification (ADIAC, du Buf and Bayer 2002b). ADIAC attained better‐than‐human identification success (du Buf and Bayer 2002a), but, in spite of this, failed to achieve a broad practical impact. This can be explained by a lack of widespread availability of the hardware and software components required for implementing the ADIAC workflow. However, now this situation is changing, with research addressing automated light microscopic diatom imaging and identification starting to appear again. These recent activities targeted automated microscopic imaging (Kloster et al. 2017), image segmentation and feature extraction (Kloster et al. 2014, Rojas Camacho et al. 2017), and taxonomic identification of images (Bueno et al. 2017, Pedraza et al. 2017).

Development of microscope imaging and image analysis methods for automatic identification has in the past been seen as distinct from, or irrelevant to, traditional taxonomy. Although it is clear that development of training image sets for automated identification needs traditional taxonomic expertise, the possible benefits of an interaction in the other direction have hardly received any attention. It is, however, possible that the everyday practice of diatom taxonomy (and of diatom analysis in general) could benefit from applying (the admittedly incomplete and imperfect, currently available) methods developed in the context of automatic identification. Aspects of potential relevance for taxonomy include: (i) using automated microscopic imaging to generate large numbers of standardized, high quality microphotographs; (ii) sharing such image sets for testing identification agreement, reflecting upon the latter to improve taxon concepts, and finally making them available as taxonomic gold standards both for future human and algorithmic identification; (iii) characterizing large sets of photographed specimens quantitatively using automated image analysis procedures; and (iv) comparing (hypothetical) taxa using numerical‐statistical methods.

This paper explores this two‐way interaction between diatom alpha taxonomy and methods developed in the context of automatic identification. The study remains within the confines of light microscopy, but uses novel, semiautomated approaches for imaging and image analysis, as well as multiexpert taxonomic annotation of a relatively large image set from a small, but taxonomically problematic target group. As an initial exploration of the possible uses of automated methods in diatom taxonomy, we addressed questions such as: are extended focus depth micrographs obtained using a highly standardized, semiautomated procedure useful for both human and image analysis based taxonomic identification? To what extent do experts agree in their identifications of such images of specimens from a highly difficult taxonomic group? Is it possible to quantitatively capture morphological features which are considered as taxonomically informative, but are normally only communicated verbally (such as heteropolarity or presence of a central expansion of the valve outline)? Can simple reflection upon cases generating disagreement, and/or quantitative morphometric analyses help refine the delimitation of the concerned taxa?

The group of taxa targeted herein includes three species from the diatom genus *Fragilariopsis*:* Fragilariopsis obliquecostata*,* Fragilariopsis ritscheri*, and *Fragilariopsis sublinearis*, the separation of which was the subject of intense discussion during the 2015 Polar Marine Diatom Workshop in Salamanca (Hoff and Rigual‐Hernández 2015). The genus *Fragilariopsis* contains around 30, mostly pelagic and sea ice‐related species, many of which occur in the polar regions and include important paleoceanographic indicators (Gersonde et al. 2003, Armand et al. 2005, Crosta et al. 2005, Cefarelli et al. 2010). The three target species are endemic to the Southern Ocean, are highly similar morphologically, and are differentiated in the light microscope almost exclusively by noncategorical characters such as different aspects of size, striation pattern, and valve shape (Hasle 1965, Cefarelli et al. 2010). The main taxonomic aim of this study was to clarify some of the remaining difficulties regarding separation criteria of these species, following on from Cefarelli et al. (2010). It is important to note that some of the material used for this study was selected because it contained problematic morphologies belonging to *F. obliquecostata*/*ritscheri*. Hence, the survey is not representative of overall morphological variation of these taxa in the field, but has a deliberate bias toward problematic specimens.

## Materials and Methods

### Samples

The Hustedt diatom collection (herbarium code BRM) was the main source of material for this research (Table 1) and allowed us to include the slides observed by F. Hustedt (including the type slide of *F. ritscheri*) and by G. Hasle for the publications which laid the foundations for current species concepts of the three target taxa (Hustedt 1958, Hasle 1965). Meta‐data on BRM slides can be obtained online via http://hustedt.awi.de. In addition, several slides from sediment core PS1768‐8 from the South Atlantic (https://doi.org/doi.pangaea.de/10.1594/pangaea.108079) and three slides from East Antarctica that contained problematic forms were included in the analyses (Table 1). With the exception of slides from sediment core PS1768‐8, each image can be traced back to its slide of origin by file name. Images from sediment core PS1768‐8 can be traced back to their slides of origin and core depth using the information recorded in the file “Fragilariopsis‐SHERPA‐output.csv” in the accompanying data archive on PANGAEA (https://doi.org/10.1594/pangaea.879785).

**Table 1:** Slides used in this study

Slide name/nr.	Latitude	Longitude	Sampling date	Sample type	Remarks
PS1768‐8	−52.593	4.476	11‐11‐1989	Sediment core	Several slides, from core depths 60, 80, 100, 110, 120, 130, 140,150, 160, 170, 180, 190, 200, 760, 780, 830, 840, 850, and 870 cm
BRM Wa‐75b	−67.7	−90.233	02‐16‐1948	Water column	Brategg expedition, lectotype of *Fragilariopsis ritscheri*
BRM Wa‐77b	−51.483	−0.133	1938/39^a^	Salp gut	Gut contents of *Salpa fusiformis*, lectotype of *Fragilariopsis separanda*
BRM ANT33‐51	−70.51	−8.195	12‐22‐2011	Water column	Polarstern exp. ANT‐XXVIII/2, station PS79/45‐1, Apstein net 20 μm
BRM ANT33‐76	−68.979	0.014	12‐24‐2011	Water column	Polarstern exp. ANT‐XXVIII/2, station PS79/47‐2, Apstein net 20 μm
BRM ANT33‐100	−67.006	0.061	12‐25‐2011	Water column	Polarstern exp. ANT‐XXVIII/2, station PS79/49‐2, Apstein net 20 μm
BRM Hasle22‐40	−68.667	−90.55	02‐12‐1948	Water column	Hasle slide from Brategg expedition, station 49
BRM Hasle22‐47	−65.617	−71.783	02‐22‐1948	Water column	Hasle slide from Brategg expedition, station 56
BRM Hasle22‐48	−66.067	−69.933	02‐22‐1948	Water column	Hasle slide from Brategg expedition, station 57
NBP‐1402.945‐946cm	−66.184	120.502	02‐21‐2014	Sediment core	NB Palmer expedition 2014‐02, JPC27, 544 m water depth
NBP‐1402.960‐961cm	−66.184	120.502	02‐21‐2014	Sediment core	NB Palmer expedition 2014‐02, JPC27, 544 m water depth
NBP‐1402.999‐996cm	−66.184	120.502	02‐21‐2014	Sediment core	NB Palmer expedition 2014‐02, JPC27, 544 m water depth

a No exact sampling date specification available; sample originates from the 1938/39 German Antarctic Expedition led by A. Ritscher.

### Imaging

**Figure 1:**
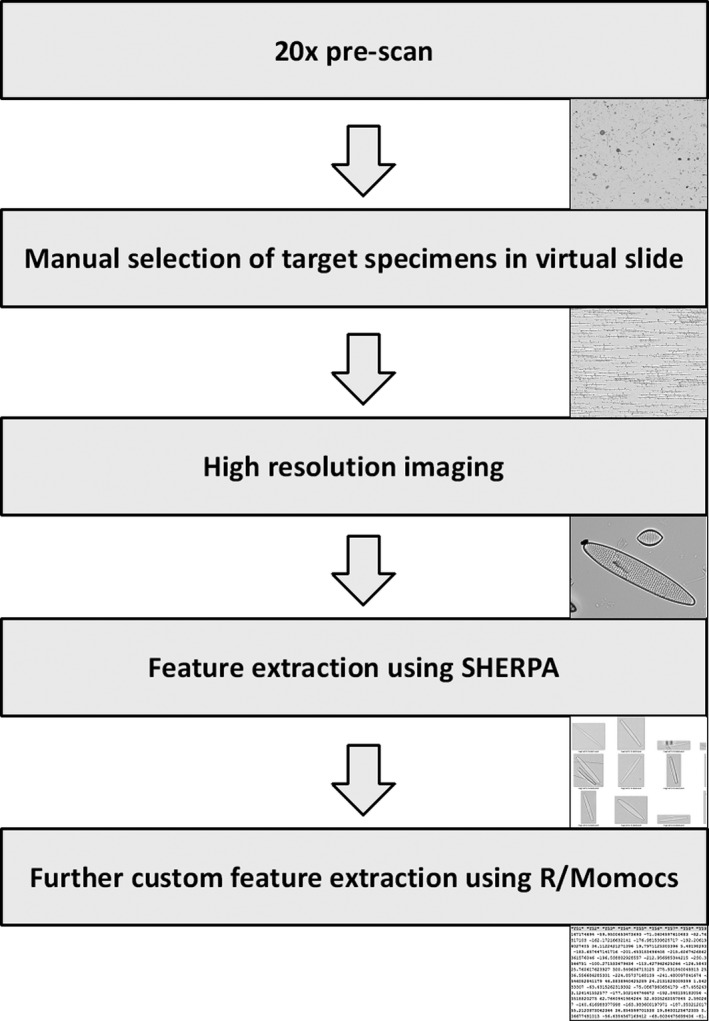
Overview of image and data acquisition workflow.

Imaging and image analyses were performed as described in Kloster et al. (2017), with the exception that for high resolution imaging, valves were selected manually after a low‐resolution prescan of the slides. This manual selection was necessary because of our focus on taxa that tend to occur in low abundances. A manually marked area of each slide was scanned with a 20× objective (ZEISS plan neofluar, NA = 0.5) in overlapping fields‐of‐view using a Metafer slide scanning system (MetaSystems, Altlussheim, Germany; individual field‐of‐view images had 1,360 × 1,024 pixels at 3.1 pixels · μm^−1^). Field‐of‐view images were combined into virtual slides (large overview images zoomable to full original resolution) using the VSlide software (MetaSystems, Altlussheim, Germany). Target valves were located and marked manually in these virtual slides. These positions were then imaged in a second step with a 63× oil immersion objective (ZEISS plan apochromat, NA = 1.40; again 1,360 × 1,024 pixels, at 9.8 pixels · μm^−1^) at 20 focus positions in 0.2 μm distances with the Metafer system. The 20 focus plane images were combined to produce an extended depth‐of‐focus image (performed as part of image processing by the Metafer image acquisition software). Figure 1 provides a schematic overview of the process.

### Species identification

Five hundred and twenty‐seven specimen images were shared with the 12 participants in the manual identification study via a Google Docs table containing basic morphometric information (valve length and width, stria density, as measured by SHERPA, see section Image analysis) alongside the extended focus specimen images. Each individual recorded their taxonomic identification and further remarks in a personal copy of this table, in order to keep individual identifications independent. Not all participants labeled each image, and not all specimens were judged to belong to one of the three target species. After completion, individual identifications were collated and compared. Duplicate images of identical specimens, as well as images depicting nontarget species according to the majority of participants, were removed before further analyses, resulting in a final set of 501 specimen images. The taxonomic label provided by each expert for each image was placed into one of four categories: *ritscheri*,* obliquecostata*,* sublinearis*, and ambiguous (i.e., difficult to decide between two or more species). The majority vote identification, defined as the label with the highest number of votes from the participants, was then determined for each image, and the percentage of votes for this assignment relative to all votes provided for the specimen in question calculated. In four cases, two of the five categories received equal numbers of votes; here the majority vote identification was set to ambiguous.

To help interpret the results, the participants were separated into two groups reflecting their taxonomic expertise with the taxa of interest. The experienced group included participants who had several years of experience identifying the target taxa. The novice group included participants with varying degrees of experience with diatom identification, but not with the target taxa, i.e., they learned to differentiate the target taxa for this study based on available taxonomic literature (Hasle 1965, Hasle and Medlin 1990, Scott and Thomas 2005, Cefarelli et al. 2010).

### Image analysis

Segmentation and initial extraction of morphometric features from extended focus images were performed using SHERPA (Kloster et al. 2014). Additional features which were considered taxonomically informative in the target group were quantified from the outlines as segmented by SHERPA using R 3.2.0 (R Core Team 2015) and the package Momocs (Claude 2014). Although SHERPA calculates elliptic Fourier descriptors (EFDs), these were recalculated using Momocs after an alignment procedure. The reason for this recalculation was that, during initial data exploration, it was noticed that the heteropolarity of several valve outlines in the data set led to bimodal within‐group distributions of EFDs, which could be remedied by aligning outlines accounting for heteropolarity. For this, the 60 points exported by SHERPA along each valve outline were aligned with their major axis to the *x*‐axis of the coordinate system, centered on the midpoint of their major axis, and the slope of the regression line of absolute *y*‐values against the *x*‐values of the outline points was determined. If this was negative, the outline was flipped around the *y*‐axis and the starting point of the chain code was shifted accordingly. The coordinates of 60 points on each valve outline can be found in the file “Frag‐3spp‐all‐Gabor‐2.txt” as variables X1‐X60 and Y1‐Y60, whereas the original outline coordinates preceding the alignment procedure can be found in the files called “*.XY_EFA.csv” in the subfolder “SHERPA output” in the accompanying PANGAEA data archive. Aligned outline coordinates were used for calculating EFDs (the values of which can be also found in the main data file “Frag‐3spp‐all‐Gabor‐2.txt” in the accompanying PANGAEA data archive). Fourteen EFDs (corresponding to 14 × 4 = 56 variables in total) were kept for further analyses because these captured 99.9% in cumulated harmonic power in the data set as determined by the function *calibrate_harmonicpower()* from the Momocs package.

Aspect ratio, the ratio between valve length and width, was among the features quantified using SHERPA. Heteropolarity was quantified by dividing each object outline on the minor axis of their best fitting ellipse, and dividing the difference in the areas of these two nearly‐half‐valves by total valve area; this number is referred to as the heteropolarity index or simply as heteropolarity in the following text, although it only partially captures heteropolarity as perceived by a diatomist. To characterize the presence of a central expansion (bulge) of the valve, five convexity defect measures were used (determined by SHERPA): convexity by perimeter, convexity by area, convexity defection factor (CDF), percent concave area fraction (PCAF), and convex hull maximum distance factor (CHMDF; Kloster et al. 2014). To quantify the eccentricity of the broadest valve position along the apical axis (which can be considered another aspect of heteropolarity), the distance of the broadest position of the valve from the broader apex (as determined in the above alignment procedure) along the apical axis was divided by total valve length.

Stria density was approximated by measuring the average distance of virgae using an approach customized for the investigated species which was implemented in SHERPA 1.1c as available at http://www.awi.de/sherpa. For this purpose, the valve image was segmented by the Adaptive Thresholding filter, resulting in a binary image where contrast‐rich edges are marked, highlighting mostly virgae (and sometimes also high contrast edges of areolae). The central 80% of a line along the valve apical axis of this segmented image was analyzed, with highlighted segments taken as relevant structures. The center points of these segments were used to construct an image depicting the positions of virgae along the apical axis, each 5 pixels wide. Stria edges were smoothed by a binomic filter to reduce overrepresentation of high frequencies in the Fourier spectrum. A forward one‐dimensional discrete Fourier transform was performed on this artificial stria/virga image, and the average distance of neighboring striae/virgae calculated from the location of the maximum of the Fourier spectrum. The result of this stria density analysis was checked manually for each image by overlaying dots corresponding to the determined average costae distance onto the image of the valve. The results were accepted as accurate in 435 cases by this manual check. For the remaining 66 images, as well as for 49 additional images for which stria density measurement using SHERPA was accepted, stria density was also determined manually by measuring the distance covered by 5 striae along the apical axis in ImageJ (Schneider et al. 2012). To validate the SHERPA measurements, the values determined manually and those using SHERPA for the latter 49 specimens were compared (Fig. S1 in the Supporting Information). The largest relative difference between both values was found to be below 15%. This was considered good agreement, in light of the precision of manual determination of stria density, and of the fact that stria density also varies with position along the valve.

To quantify stria orientation, each image with the background masked out (as exported by SHERPA with every pixel outside the valve outline set to a gray value of 0) was rotated, so that the major axis of the specimen was vertical, and cropped to the width of the original image. The integrated response of a Gabor filter with a periodicity fixed to average stria distance (as determined by SHERPA) converted into pixels was maximized by numerical optimization, in principle finding an average stria orientation over the middle portion of the valve face, using the R function *optim()*.

### Statistical analysis

Statistical analyses were performed in R 3.2.0 (R Core Team 2015). Univariate analyses of variance (ANOVA), as well as bivariate analyses of covariance (ANCOVA), were performed using the *lm()* function; *P*‐values associated with individual coefficients are reported as provided by *summary.lm()* and a *P*‐value significance limit of 0.05 is used. For visualizing group‐wise distributions of individual variables, the sinaplot package (Sidiropoulos et al. 2015) was used. For multivariate classification, the functions *naiveBayes()* and *svm()* from the R package e1071 (Meyer et al. 2015); *lda()* and *qda()* from MASS (Venables and Ripley 2002); and *randomForest()* from package randomForest (Liaw and Wiener 2002) were used. Three sets of features were used in three sets of classification experiments. The first feature set referred to as non‐EFD features included area, perimeter, length and width of valves; the heuristic shape descriptors rectangularity, compactness, ellipticity, triangularity, and roundness; the convexity indices convexity by perimeter, convexity by area, CDF, PCAF, CHMDF; and aspect ratio, stria density, stria orientation, and relative location of broadest position. The second set of features included the 56 coefficients of the 14 EFDs. The third set of features was a combination of the previous two.

Images, data and analysis scripts for each substantial step of the study are provided in a Supplementary archive available from PANGAEA under https://doi.org/10.1594/pangaea.879785.

## Results

### Introducing the target species

**Figure 2:**
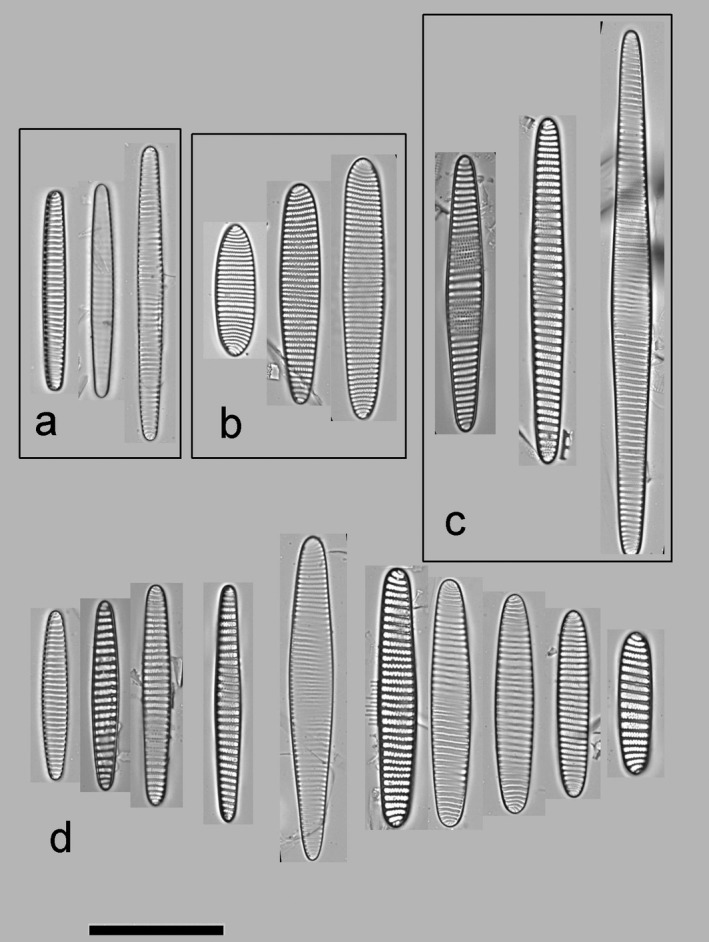
The target taxa: a selection of typical (a–c) and difficult (d) specimens. (a) *Fragilariopsis sublinearis*: narrow‐linear valve shape, fibulae often visible; (b) *Fragilariopsis ritscheri*, broad‐elliptical, heteropolar valve shape; (c) *Fragilariopsis obliquecostata*, oblique striation pattern, valve outline expanded around center; (d) difficult‐to‐identify specimens showing combinations of characters considered typical of different species, for instance slightly elliptic or centrally expanded valves with straight striae and markedly visible fibulae; strong heteropolarity with slight central expansion; or elliptic valve shape with oblique striae.

To help interpret the following sections, a short introduction of each target taxon is provided based on the literature. *Fragilariopsis sublinearis* and *F. obliquecostata* were described by Van Heurck (1909), whereas the third species, *F. ritscheri*, was described later by Hustedt (1958). The key references on the current taxonomy of the group are Hasle (1965) and Cefarelli et al. (2010). Summarizing the characters in these references observable using LM, *F. sublinearis* is 30–92 μm long, has the narrowest and most linear valve outline of the three species, is isopolar, has poroids near the resolution limit, and fibulae that are often clearly discernible in the LM (Fig. 2a). *Fragilariopsis ritscheri* is between 22 and 57 μm long, has wider valves and a more elliptic valve outline than the other two species, and shows a pronounced heteropolarity; virgae generally straight except toward the broader apex, and poroids are generally small but can be resolved in LM (Fig. 2b). Finally, *F. obliquecostata* is between 48 and 125 μm long, has an oblique striation pattern, a central expansion of the valve outline, isopolar to slightly heteropolar valve outline, and poroids that are generally coarser than in the two other species (Fig. 2c). In spite of the clarity of these descriptions, differentiating between small *F. obliquecostata* versus large *F. ritscheri* specimens (Hasle 1965), as well as between large *F. sublinearis* and small *F. obliquecostata* specimens (Cefarelli et al. 2010), has proved difficult. To visually illustrate the nature of the difficulties, some examples are provided of valves with character combinations which make the application of the published differentiating criteria less than straightforward (Fig. 2d: for example, narrow‐linear or broadly elliptical valve shape in combination with oblique striae; or central expansion together with pronounced heteropolarity). Confronted with such character combinations, which order of preference or weighting should be given to individual traits for separating the taxa? In the following sections, an answer to this question is attempted through automated analysis of light micrographs and taxonomic identifications attached to these images by several diatomists.

### Comparison of expert identifications

All participants in this study were in complete taxonomic agreement for 33.1% (166 of 501) specimens. The number (percentage) increased to 281 (56.1%), 370 (73.9%), and 421 (84.0%) when disagreement by one, two, and three participants, respectively, was allowed. When comparing results for the eight participants in the experienced group, 63.2% (307 images of the 486) were identified in full agreement, whereas the four participants in the novice group agreed in 51.3% of cases (134 of 261 specimens). As pointed out in the Introduction, it should be borne in mind that some of the samples were deliberately chosen because they were considered taxonomically problematic.

**Figure 3:**
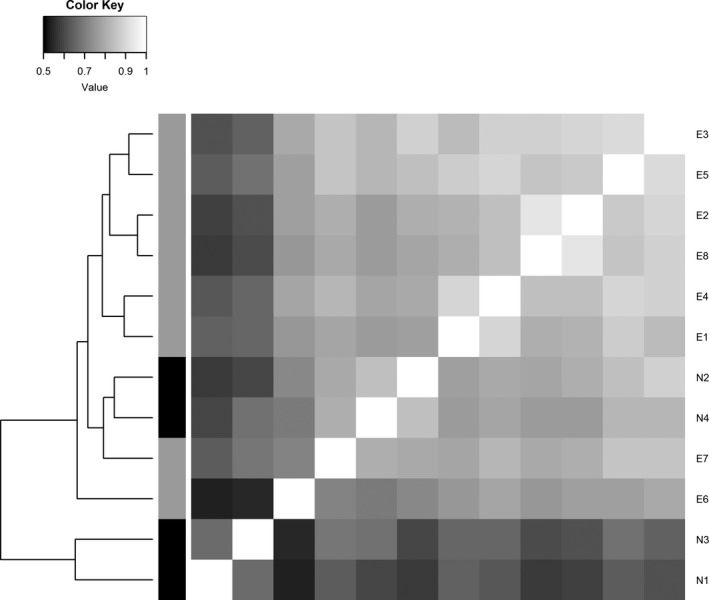
Pairwise similarities between sets of identifications, calculated as the proportions of identical, nonempty, nonambiguous identifications between pairs of investigators, displayed as a heatmap. The matrix is symmetric around the white diagonal since pairwise similarities between pairs of participants are symmetric. Stripes to the left of heatmap: black: novice; gray: experienced participant. Lighter color in the heatmap signifies higher agreement between a pair of participants. Note that experts E3 and E5 show the highest overall agreement with all other participants, i.e., they represent a central tendency around which individual identifications are spread. Interestingly novice participants N2 and N4 are most similar to each other and to E3 in their identifications. The two other novice participants N1 and N3 appear as outliers compared to all other participants.

Figure 3 depicts the pairwise similarities of individual expert identifications in the form of a heatmap and clusters participants on this basis. Whereas two of the novice participants (N2 and N4) grouped well within the expert group, two others (N1 and N3) appeared not only as outliers when compared to the experts, but they also clustered together, indicating that their concepts of the taxa were in some agreement but diverged from the more experienced participants.

**Figure 4:**
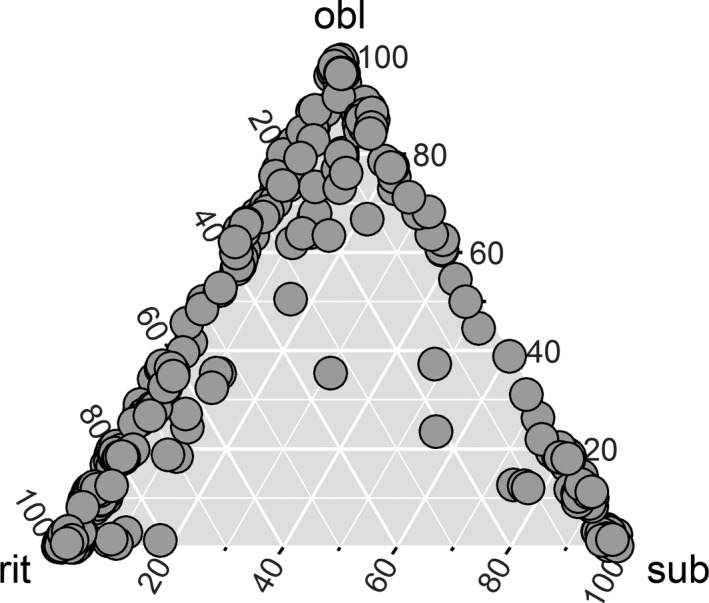
Ternary plot showing how votes for each image were distributed among the three species. Species names are abbreviated as: obl for *Fragilariopsis obliquecostata*; rit for *Fragilariopsis ritscheri*; and sub for *Fragilariopsis sublinearis*. Each circle represents a specimen; their distance from each tip of the triangle, when measured along the height of the triangle ending in that tip, corresponds to the percentage of participants who labeled the concerned specimen with the species name represented by that tip. Hence, closeness to each corner of the triangle represents strong agreement in taxonomic labeling, whereas positions near the midpoint represent the most equivocal cases. Points at the tips represent unequivocally labeled specimens (100% of votes for a single name); those along vertices represent specimens which received two different labels (0% of votes for a single name), and points in the inner area of the triangle mark specimens which received three different labels from different participants. Slight random noise was added to percentage distribution of votes to reduce overplotting.

Disagreement was more pronounced for particular length ranges, especially between 60 and 90 μm, and again slightly at 100–110 μm (although the number of specimens in the latter range was low and thus this result is less robust, Fig. S2 in the Supporting Information). The 60–90 μm length range represents the range over which *Fragilariopsis obliquecostata* and *F. ritscheri* are thought to overlap. Indeed, most disagreement in labeling occurred between this pair of species, and participants separated *F. ritscheri* from *F. sublinearis* in substantially more agreement (Fig. 4). Nevertheless, several specimens of the latter pair also generated disagreement.

Specimens substantially beyond previously published length ranges were identified as *Fragilariopsis ritscheri* or *F. obliquecostata*, although not always in high agreement. For instance, the longest specimen identified unequivocally by all participants as *F. ritscheri* was 57.6 μm long, but the longest specimen which was identified as *F. ritscheri* by the majority was 93.7 μm long, and even a 103.1 μm long specimen received two *F. ritscheri* votes (both from the experienced group; Table S1 in the Supporting Information). Several similar examples can be seen in Table 2 and Tables S1–S3 in the Supporting Information for the other species and other features as well.

**Table 2:** Updated statistics of morphometric characters for the three investigated species. For each character, range is followed by average ± standard deviation in parentheses. The number of observations (*n*) for each species is identical as specified in the column header for all features except stria orientation, for which *n* is given in addition in the parentheses. For readability, and since both indices are bounded to the 0‐1 interval, the values of the heteropolarity index and of the eccentricity of broadest position are converted to percentages

	*Fragilariopsis obliquecostata* (*n* = 135)	*Fragilariopsis ritscheri* (*n* = 293)	*Fragilariopsis sublinearis* (*n* = 67)
Valve length (μm)	32.2–120.5 (67.8 ± 16.8)	20.3–93.7 (50.7 ± 12.9)	30.7–75.3 (51.4 ± 11.1)
Valve width (μm)	5.9–10.7 (8.16 ± 0.96)	6.3–11.3 (8.62 ± 0.88)	5.1–7.4 (6.21 ± 0.49)
Aspect ratio	4.0–14.5 (8.4 ± 2.0)	2.4–11.0 (5.9 ± 1.6)	4.8–13.2 (8.3 ± 1.9)
Heteropolarity index (%)	0–7.9 (1.7 ± 1.4)	0.4–8.8 (3.9 ± 1.5)	0–4.0 (0.8 ± 0.7)
Eccentricity of broadest point (%)	36.7–60.0 (50.1 ± 4.4)	26.7–62.1 (47.2 ± 5.4)	38.7–71.0 (50.8 ± 5.9)
Stria density (1 in 10 μm)	4.7–9.6 (6.5 ± 1.0)	5.2–10.4 (7.4 ± 1.1)	6.0–10.1 (8.2 ± 0.7)
Stria orientation (° to transapical)	0.3–18.9 (6.1 ± 3.4, *n* = 127)	0–16.0 (1.8 ± 2.3, *n* = 251)	0–16.7 (3.9 ± 3.2, *n* = 52)

The clustering in Figure 3 shows that all experts were in high agreement with expert E3 (and, to a slightly lower extent, with E5). This means that the identifications of E3 in some way represent the central tendency in the spread of identifications among experts. Based on this, one could designate the identifications by expert E3 to be the gold standard for identifying the three species. However, a potentially preferable alternative, acknowledging that even the best expert might be wrong occasionally (and that this could be recognized by her/his deviation from the majority of other experts), would be to simply say that the gold standard is defined by how the majority of experts identified a specimen (Kelly et al. 2011, Schoening et al. 2016). For the following analyses, we took this latter approach and grouped specimens into one of the three taxa based on majority votes.

### Morphometric comparisons

As a next step, an attempt was made to identify quantitative features which might statistically discriminate the three species. For this, some generic, mostly outline‐based features were used, and, in addition, an attempt was made to capture as numeric feature descriptors some quantitative traits on which the experts reported that they based their identifications (Fig. 1).

**Figure 5:**
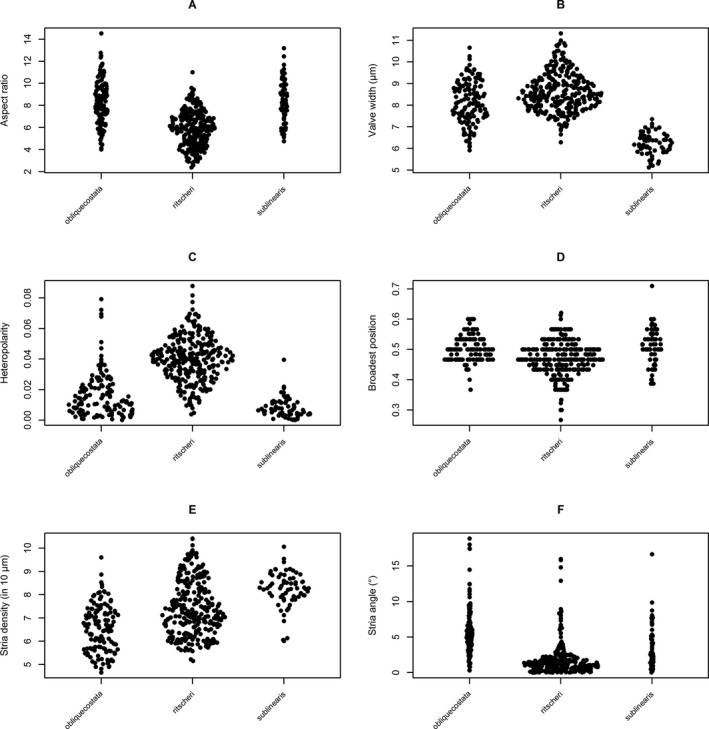
Group‐wise distributions of six morphometric characters in the three taxa, as reflected in the majority votes of participants. Position on the *y*‐axis represents the value of an observation, relative spread of points within groups on the *x*‐axis is random noise proportional to the density distribution of observations (sinaplots). (A) aspect ratio; (B) valve width in μm; (C) heteropolarity index (unitless, in the range between 0‐1); (D) eccentricity of broadest position (unitless, in the range between 0‐1); (E) number of striae in 10 μm; (F) orientation of striae (in degrees, relative to the transapical axis of the valve).

In all cases, distributions of feature values among the three species overlapped (Fig. 5), but there were statistically significant differences (as tested using ANOVA; Figs. S3 and S4 in the Supporting Information).

Heteropolarity index (*F*_2,492_ = 216.3, *P* < 10^−4^), stria density (*F*_2,492_ = 62.66, *P* < 10^−13^) and orientation (*F*_2,492_ = 95.67, *P* < 10^−5^), rectangularity (*F*_2,492_ = 30.45, *P* < 10^−10^), and three of five convexity indices tested (convexity by area, CDF, *F*_2,492_ = 22.24, *P* < 10^−9^; and PCAF, *F*_2,492_ = 92.5, *P* < 10^−15^), showed highly significant differences among all three taxa.

Aspect ratios (ANOVA *F*_2,492_ = 116.2, *P* < 2 × 10^−16^), eccentricity of the broadest position along the apical axis (*F*_2,492_ = 21.88, *P* < 2 × 10^−7^), and the heuristic shape descriptors compactness (*F*_2,492_ = 93.55), form factor (*F*_2,492_ = 92.3), and roundness (*F*_2,492_ = 81.1; in all three cases, *P* < 2 × 10^−16^) showed significant differences for *F. ritscheri* compared to the other two species, but not between the latter pair. Finally, there were significant differences in convexity by perimeter (*F*_2,492_ = 3.6, *P* = 0.029) and CHMDF (*F*_2,492_ = 13.52, *P* < 10^−5^) between *F. sublinearis* and the other two species, but not between the latter two. The heuristic shape descriptors ellipticity (*F*_2,492_ = 1.7, *P* = 0.188) and triangularity (*F*_2,492_ = 1.69, *P* = 0.186) did not show any significant between‐species differences.

**Figure 6:**
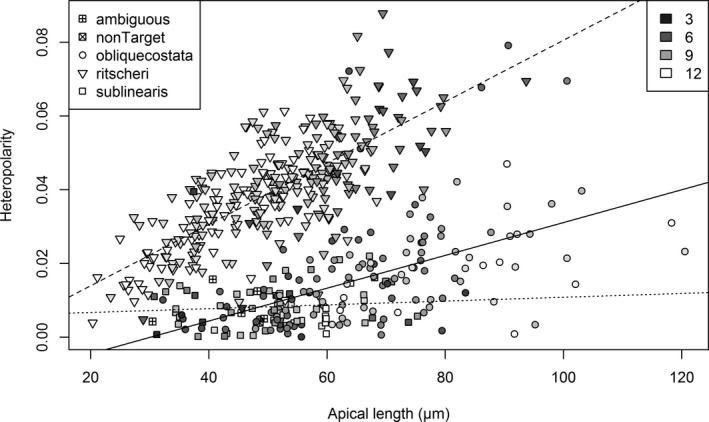
Heteropolarity index versus valve length. Different plotting symbols represent the majority identification assigned to each specimen (legend at upper left); gray levels reflect the level of agreement in the identification of each specimen (number of votes counted for the majority identification; legend upper right; i.e., specimens identified in higher agreement appear lighter). The lines represent group‐wise least squares linear regression for *Fragilariopsis obliquecostata* (solid line); *Fragilariopsis ritscheri* (dashed); and *Fragilariopsis sublinearis* (dotted).

**Figure 7:**
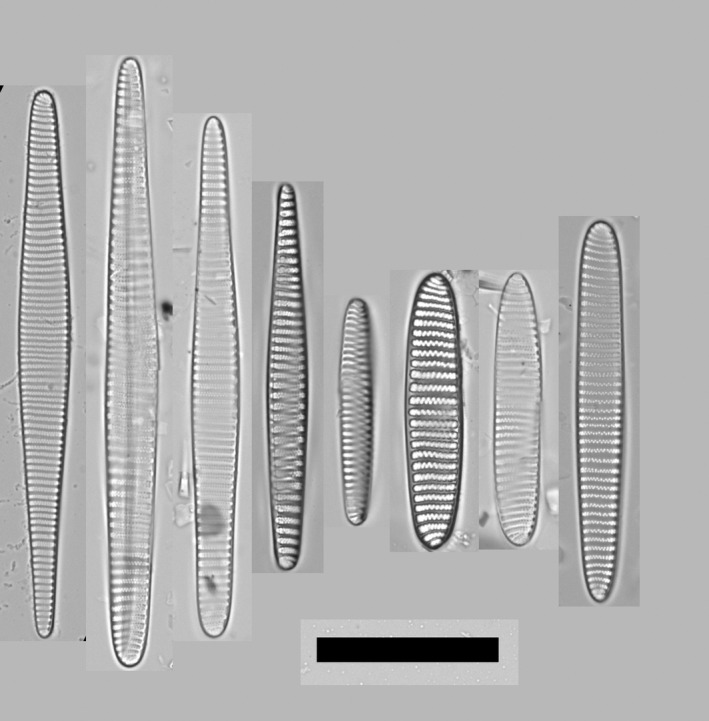
The most prominent group outliers in terms of heteropolarity, from left to right: four specimens identified as *Fragilariopsis obliquecostata* as majority votes, with exceptionally high values of the heteropolarity index; one specimen identified as *Fragilariopsis sublinearis* as majority vote with an exceptionally high value of the heteropolarity index; and three specimens identified as *Fragilariopsis ritscheri* as majority vote with exceptionally low values of the heteropolarity index (specimen IDs from left to right, followed by numbers of votes they received for ritscheri/obliquecostata/sublinearis/ambiguous: ANT33‐100.000106: 3/7/1/1, NBP‐1402.945‐946cm.000040: 3/8/0/1, NBP‐1402.945‐946cm.000066: 4/7/0/1, PS1768‐8.000769: 2/6/1/3, PS1768‐8.000855: 0/2/5/2, PS1768‐8.000578: 5/3/1/2, NBP‐1402.945‐946cm.000082: 11/0/0/0, NBP‐1402.999‐996cm.000007: 11/1/0/0). Scale bar = 30 μm.

As an example, we provide further detail on heteropolarity, a character considered important for differentiating *Fragilariopsis ritscheri* from *F. obliquecostata*. In agreement with expert opinion, a plot of the heteropolarity index against valve length (Fig. 6) shows that *F. ritscheri* (mean heteropolarity index 3.9%) tends to be more heteropolar at all sizes than *F. obliquecostata* (1.7% on average) and *F. sublinearis* (0.08%). It also shows that heteropolarity increases pronouncedly with valve length in *F. ritscheri*. Heteropolarity is independent of valve length in *F. sublinearis*, whereas in *F. obliquecostata* it shows a slight positive trend, but not as strong as in *F. ritscheri*. In spite of the overlap (Figs. 5C and 6), the three species, as defined by expert consensus, are clearly distinguishable statistically, both in univariate (ANOVA, *F*_2,492_ = 216.3, *P* < 10^−4^ for all group coefficients) and bivariate (regression against length of apical axis) comparisons (ANCOVA, all coefficients with *P* < 0.016, *F*_5,489_ = 233.8). However, there were some outlier cases that did not conform to this general pattern, including specimens identified as *F. obliquecostata* and *F. sublinearis* with atypically high heteropolarity values, as well as valves highly consistently identified as *F. ritscheri* with low values of the heteropolarity index (the most prominent outliers are shown in Fig. 7). Whether these specimens represent rare genuine outliers in terms of their heteropolarity for their respective taxa, or if their consensus identification is incorrect, cannot be ultimately answered yet. However, these examples do illustrate how explicit quantification can help to reflect upon ideas of taxon delimitation. In this case, a conflict between heteropolarity (considered typical of *F. ritscheri*) versus presence of a central expansion (typical of *F. obliquecostata*) becomes apparent. A resolution of this conflict is proposed below (in the section Updated differential diagnoses).

### Multivariate classification attempts

Univariate comparisons showed statistically significant differences among the three species, albeit with overlapping ranges. In the algorithmic identification experiments of this study, automatic identification algorithms were tested to see how well they could identify the three species using combinations of these features.

**Figure 8:**
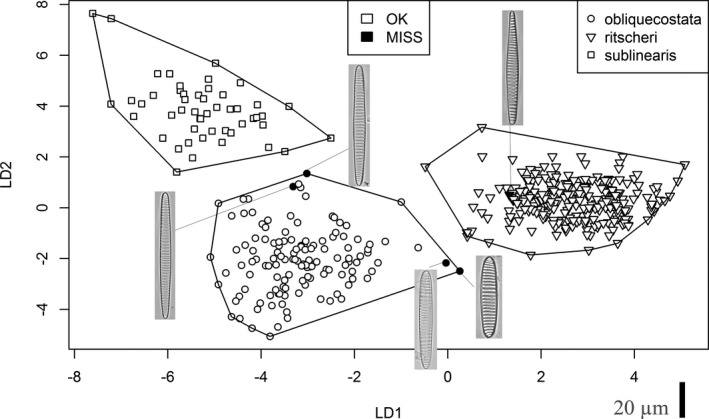
Linear discriminant scores based on the combined (non‐EFD plus EFD) feature set. Plot symbols (legend upper right) indicate the taxon into which the LDA model classified each specimen. In the case of five specimens, these classifications differed from the majority vote, these points are plotted with filled symbols, whereas specimens classified by the LDA model in agreement with the majority vote are shown by empty symbols. The images of the five specimens misclassified by the LDA model are inserted and linked with a gray line to the corresponding points (all at the same scale; scale bar at bottom right corresponds to 20 μm). Specimen IDs in increasing order of LD1 scores (i.e., left to right on the plot): PS1768‐8.000425, PS1768‐8.000423 (majority vote for both: *Fragilariopsis sublinearis*); NBP‐1402.945‐946cm.000065, PS1768‐8.000578 (majority vote for both: *Fragilariopsis ritscheri*); and PS1768‐8.000769 (majority vote: *Fragilariopsis obliquecostata*).

Three series of identification experiments were carried out using three sets of features: non‐EFD features (see details in the Materials and Methods section), EFDs, and a combined set of both types of features. For each set of features, a series of classification algorithms was tested, ranging from naïve Bayes classifier through linear and quadratic discriminant analysis (LDA/QDA) to support vector machines (SVM) and random forests (Table 3). Not surprisingly, an increasing amount of information (number of features) and nonlinearity of classification algorithms led to improved performance (as measured by the number of misclassifications). While naïve Bayes classifiers showed a relatively poor performance, LDA, QDA, and SVM gave substantially better results, and a random forest with 500 learners was able to differentiate the three species in complete agreement with majority votes no matter which data set was used (although this high apparent performance represents serious overfitting, as the cross‐validation results below show). As an example, more detail on linear discriminant analysis of the combined (non‐EFD plus EFD) feature set is presented in Figure 8, highlighting those specimens for which expert consensus identification was in conflict with the LDA results.

**Table 3:** Summary of results of classification experiments. The three columns represent the three data sets used in the experiments: non‐EFD stands for the set of morphometric variables excluding elliptic Fourier descriptors (19 variables); EFD: elliptic Fourier descriptors (4 × 14 = 56 variables); both: both sets of variables combined (75 variables). The rows stand for classification algorithms as follows: nBayes: naïve Bayes classifier without cross‐validation; LDA: linear discriminant analysis without cross‐validation; QDA: quadratic discriminant analysis without cross‐validation; SVM: support vector machine without cross‐validation; rForest: random forest without cross‐validation; SVM‐cv: support vector machine with 10‐fold cross‐validation performed in 1,000 replicates; rF‐cv: random forest with 10‐fold cross‐validation performed in 1,000 replicates. Table entries for analyses without cross‐validation represent number of misclassified cases out of 430, followed by the percentage this represents in parentheses. For cross‐validation analyses, average percentage of misclassified cases as measured on an independent test set is given, followed by the range of the same quantity across 1,000 random replicates in parentheses

	Non‐EFD	EFD	Both
nBayes	60 (14%)	39 (7.9%)	25 (5.8%)
LDA	15 (3.5%)	10 (2.0%)	5 (1.2%)
QDA	16 (3.7%)	1 (0.2%)	N.A.
SVM	11 (2.6%)	11 (2.2%)	4 (0.9%)
rForest	0	0	0
SVM‐cv	5.6% (1%–12%)	6.7% (1.6%–15.3%)	3.6% (0%–9.3%)
rF‐cv	5.9% (0%–14.8%)	5.5% (0.8%–12.9%)	4.0% (0%–11%)

The two best performing algorithms (SVM and random forest) were further tested in a cross‐validation experiment. As expected, this led to a higher proportion of misidentifications (Table 3), but the results still indicate that using the measured morphometric features, automatic identification is possible with an error rate of ~10% (i.e., within the range of uncertainty when compared to the identification by experts).

## Discussion

The light microscopic delimitation of difficult‐to‐separate diatom taxa was addressed in a Southern Ocean species complex using methods not routinely applied in such a context. First, a comparison was made of the identifications of several taxonomists using a set of 501 images of three *Fragilariopsis* species, to generate a gold standard training image set. Second, a range of morphometric features (in part modeled after “real” quantitative taxonomic traits) was quantified using a semiautomated procedure, and the discriminating ability of these features, individually and in combination, was tested among the three species as defined based on expert consensus.

### Extent of taxonomic agreement

Full consensus in taxonomic labeling of individual diatom specimens (valve images) may not be given for all taxa, even among experts who in principle largely agree on their discriminative criteria. This is not unique to the present target group but has been observed in other studies as well, both with diatoms and other organisms (Culverhouse et al. 2003, Kelly et al. 2011, Schoening et al. 2016). The taxa targeted in this study, and some of the samples analyzed, were selected exactly because their separation was perceived as problematic, and this explains the lower congruence observed here when compared to the above studies. Another factor contributing to this comparatively low congruence might have been the unusual setup of the study for taxonomists: identification by observing images, rather than physical specimens directly on the microscope, proved an unusually difficult task (e.g., impossibility to focus through specimens, different scaling of different specimens).

Looking at patterns of agreement among participants, it is possible to speculate about different factors that might influence congruence in taxonomic identifications. Experienced participants agreed for almost two‐thirds of specimens in their taxonomic assignments, which was much higher than the agreement in the novice group of participants (diatomists without specific expertise with the target taxa: 51.3%). It is not possible to reliably tease apart the relative importance of experience versus communication among experts in this study since most of them regularly participate in the Polar Marine Diatom Workshops (https://polarmarinediatomworkshop.org), a platform for regular exchange of taxonomic knowledge, among other activities. Other studies indicated that such exchange is critical for reaching taxonomic consistency (Kahlert et al. 2009).

Participants generally perceived the distinction between *Fragilariopsis obliquecostata* versus *F. ritscheri* to be quite difficult in some cases, but saw the recognition of the third species, *F. sublinearis*, as unproblematic (with the exception of one participant from the novice group who found the differentiation between *F. sublinearis* and *F. obliquecostata* the most difficult). In spite of this, several specimens received votes distributed between *F. sublinearis* and *F. obliquecostata*, and some (although few) between *F. sublinearis* and *F. ritscheri* or all three taxa (Figs. 3 and 7). Some of these cases of disagreement represented a conflict between experienced versus novice opinion; however, this was not always the case and identifications of *F. sublinearis* were also not 100% unequivocal within the experienced group (Fig. 4, Appendix S1 in the Supporting Information). Thus, individual expert perception of a high certainty in morphological distinctness of a taxon is not necessarily a guarantee of full taxonomic consistency among multiple investigators.

### Morphometric characters for species discrimination

Identifications given by all participants were not in full agreement with the morphometric data given in the literature. This was not by mistake, as some participants explicitly reported that they interpreted morphometric ranges regularly provided in taxonomic descriptions (in this case, for valve length, width, stria density) with caution, knowing that they rarely cover the full range of variation occurring in nature (Crosta 2009a, Shukla et al. 2013, Kloster et al. 2017). This observation is not surprising, since increasing sample sizes (as well as increasing habitat diversity) are expected to lead to broader estimates of sample ranges (Edgar et al. 2015). Some participants (especially from the novice group) reported that if a valve with dimensions substantially outside the ranges given in the literature for a particular species was encountered, they tended to avoid labeling it as that species. It seems that with increasing experience, identifiers can rely on a broader range of features to recognize taxa that are not explicitly documented in their literature resources.

The following features were considered taxonomically informative in the case of the target taxa of this study: heteropolarity, location of the broadest position of the valve along the apical axis, the presence of a central bulge, aspect ratio, obliqueness of striae, degree of silicification, size of poroids, visibility of whitish raphe keel puncta, and shape of the apical costae. In the literature, the first systematic comparison and explicit differential diagnosis of the three target species were given by G. Hasle (Hasle 1965), which was recently updated substantially by Cefarelli et al. (2010). In G. Hasle's opinion (Hasle 1965), none of the previously suggested differentiating characters were stable, for instance, oblique stria orientation can also occur in specimens of *Fragilariopsis kerguelensis* and *F. ritscheri*, besides *F. obliquecostata*. She proposed the presence of an expansion (= bulge as termed above) of the middle part of the valve as a character unique to *F. obliquecostata*, and a less pronounced heteropolarity to differentiate it from *F. ritscheri*. The more recent comparison (Cefarelli et al. 2010) proposed to differentiate *F. obliquecostata* from *F. ritscheri* by its narrower valve shape (i.e., higher aspect ratio; but without an explicit quantification) and less pronounced heteropolarity, and reported an overlap in the length ranges of these two taxa. *Fragilariopsis sublinearis* was found to be clearly distinguishable from both these species by its narrower valve width (Table 3 of Cefarelli et al. 2010); in spite of this, and in line with our results, they stated that *F. sublinearis* can be confused with *F. obliquecostata*, and proposed the density of poroids as the main differentiating character between them, a character we were unfortunately not able to quantify in this study.

The aims of the morphological comparisons undertaken were twofold. First, they were attempted in order to bring taxonomic knowledge into the realm of automatic identification by exploring whether and how individual morphological characters judged to be of taxonomic value by experts could be quantified (as far as possible, without manual interaction). Second, the discriminating ability of those characteristics which could be quantified was tested visually and statistically.

For our first aim, it was possible to translate some of the taxonomic characters into numerical indices (aspect ratio, heteropolarity, location of broadest position, stria density and orientation). Some of these might prove more generically applicable to other diatom taxa (i.e., heteropolarity index); in other cases, further thought will be necessary for a generic formulation of more broadly useful features. A number of further characteristics remain which were reported by the participants as useful for discrimination, including the degree of silicification, clear visibility of keel puncta, poroid size, changing stria orientation along the apical axis of the valve, or the shape of the apical virgae, but which were not quantified herein. Quantifying some of these might be feasible with intelligent application of standard image analysis methods in the future.

The quantification exercise gave a picture that was broadly consistent with expert opinion about the morphological separation of the three target taxa, but it also revealed cases where different characters seemed to suggest conflicting identifications (especially the conflict between heteropolarity and presence of central expansion/oval valve shape; Figs. 6 and 7). It showed that valves identified as *Fragilariopsis ritscheri* were generally more heteropolar and their heteropolarity increased with valve length more than was the case for *F. obliquecostata*, although there were exceptions to this pattern. The broadest position of the valve was on average found to be more centrally located in the group of specimens identified as *F. obliquecostata* and *F. sublinearis* than in *F. ritscheri*. *Fragilariopsis obliquecostata* featured more oblique striae on average than the other two species. In terms of quantitative distinction, clear‐cut range gaps among the three taxa were not observed in any of these characteristics, but analyses of variance indicated a significant (at *P* ≪ 0.05) separation of the species in several features, and multivariate classification attempts reached an accuracy within the range of congruence among experts. This is encouraging for future automatic classification attempts, especially considering that an inherent limitation of the morphometric comparisons was that specimens assigned to taxa based on majority votes do not need to correspond to the “truth.” This is, however, a situation that often needs to be dealt with, i.e., whenever independent information for ground‐truthing taxonomic identifications (for example, from molecular markers), is not available, as is the case for most recent and all fossil taxa. The availability of multiple taxonomic opinions still enables the generation of useful reference image sets and corresponding training data for computational classification even in the face of, and acknowledging, taxonomic disagreement, as done here and in other studies (Culverhouse et al. 2003, Kelly et al. 2011).

### Updated differential diagnoses

Table 2 can be seen as a direct continuation of the morphometric table given by Cefarelli et al. (2010) (their table 3). The largest differences between both tables concern the minimum length for *Fragilariopsis obliquecostata* (32.2 μm vs. 48 μm) and the maximum length for *F. ritscheri* (93.7 μm vs. 57 μm), extending the range of overlap between both taxa from 9 to over 61 μm. It has been stated previously (Hasle 1965, Cefarelli et al. 2010) that valve length is not a good discriminating character between these taxa, a point that is further underlined by the explicit quantification of a larger set of specimens undertaken here.

A motivation behind the morphometric comparisons was the expectation that an explicit comparison and quantification might help reach an improved consensus on taxonomic concepts and/or to make the latter more explicit. Concerning the distinction between *Fragilariopsis ritscheri* and *F. obliquecostata*, less experienced participants generally appeared to place more importance on literature ranges in length/width or the presence of a central expansion, whereas more experienced participants gave higher weight to heteropolarity (one rounded and one more pointed end; eccentricity of broadest position). Several examples can be found in Appendix S1 where short (length ≪ 50–60 μm) and isopolar specimens were assigned to *F. obliquecostata* by some or all participants, but to *F. ritscheri* by others. On the other side of the size spectrum, long (length > 70 μm) specimens appearing heteropolar and sometimes also expanded in the middle were often called *F. ritscheri* by experienced participants, but *F. obliquecostata* by others. This conflict between heteropolarity and other traits is also illustrated by Figures 6 and 7. The consensus emerging from confronting these views among the authors is that for the distinction between *F. obliquecostata* and *F. ritscheri*, heteropolarity should be given more weight than length or the presence of a central expansion, since the latter can appear in large *F. ritscheri* specimens. Comparisons herein do not prove this distinction or favor it more than other possible distinctions, but this is put forward as a working hypothesis. One argument in support of this consensus is that length decreases substantially during vegetative growth, so it is not generally expected to be a robust differentiating character for diatoms. A second argument is that if it seems consistent with allometric shape change for one species (*F. obliquecostata*) to display a central expansion at large apical lengths, and to lack it at shorter lengths, the same phenomenon may also reasonably appear in closely related species (e.g., *F. ritscheri*). Indeed, such simplification of outline shapes with decreasing size is common in pennate diatoms (Woodard et al. 2016). A nice illustration is to compare the 94 μm long, heteropolar, centrally expanded specimen NBP‐1402.960‐961cm.000091 (a specimen far exceeding the previously reported apical length range for *F. ritscheri*, yet still identified as such by the majority of participants) with the 92 μm long, also centrally expanded, but more or less isopolar specimen ANT33‐76.000041 (a specimen identified as *F. obliquecostata* in full agreement) in Appendix S1. A final, ecological argument supporting this species distinction is the observation of somewhat bulged valves in the Subantarctic Zone of the Indian Ocean (X. Crosta, unpubl. data) which are probably not *F. obliquecostata* since that species is not known to appear so far equatorward. The distinction is important, precisely for its ecological implications: *F. obliquecostata* is recognized as an indicator of the location of summer sea ice edge in Antarctic paleoceanography (Gersonde and Zielinski 2000, Crosta 2009b, Collins et al. 2012, 2013).

**Figure 9:**
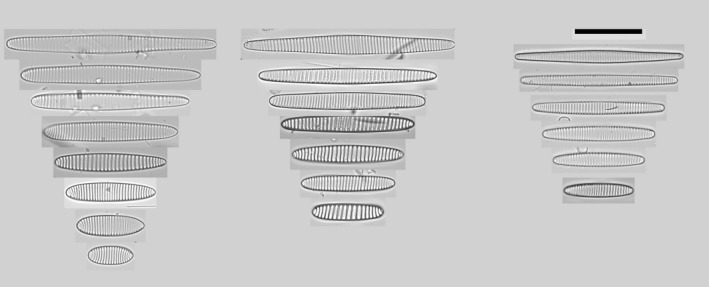
Illustrating an updated concept for the delimitation of the three taxa investigated. Left: *Fragilariopsis ritscheri*, heteropolar valves with one rounded and one pointed end, slightly eccentric broadest position, close to straight striae; broadly elliptical valve shape at smaller sizes, linear‐lanceolate, slightly expanded valves at larger sizes. Middle: *Fragilariopsis obliquecostata*, isopolar valves with oblique striae, elliptic valve shape at smaller, centrally expanded at larger sizes. Right: *Fragilariopsis sublinearis*, isopolar, narrow‐linear to slightly elliptic valve shape at lower sizes, slight central expansion at larger sizes. Scale bar: 30 μm.

A comparably clear‐cut update on the distinction between *Fragilariopsis obliquecostata* and *F. sublinearis* cannot be given here, apart from stating that the assignment of individual specimens to either of these species is perhaps also not as simple as first perceived by most participants at the start of this study. Two examples are the leftmost valves illustrated in Figure 8, but more cases can be found in the supplementary images. The clearest indication of difficulty in separating these species is that it happened that the same participant identified duplicate images of the same specimen once as *F. obliquecostata* and once as *F. sublinearis*. An important criterion to tell these species apart is whether the raphe keel puncta are clearly visible on the valve margin (the case for *F. sublinearis*). Unfortunately, this character does not seem trivial to quantify using image analysis, and, as discussed below, is not even always resolved in the extended focus depth images used here. The consensus suggests that longer valves of *F. sublinearis* might display a central expansion, similarly the other two target species. Figure 9 gives a visual summary of our updated diagnoses. We repeat here that these diagnoses should be considered a working hypothesis which can in the future be tested using independent, for instance, molecular data to arrive at a more solid concept for the delimitation of these taxa.

### (Semi) automated methods for diatom taxonomy

Beyond the taxonomic motivations, this study was also an experiment to use automated imaging and image analysis methods as a possible improvement of the more conventional taxonomic workflow. The semiautomated imaging technique in this study involves algorithmic autofocusing followed by capture of images in 20 different focus depths and combining these into extended focus depth images. This highly standardized imaging (in terms of illumination, exposure, autofocus) is expected to lead to a higher uniformity in image modalities than what can be obtained with manual microscopy, and this higher uniformity looks advantageous for downstream image analyses. This expectation was, however, only partially fulfilled by the images included in this study: in spite of a combination of autofocusing and image stacking, not all taxonomically important morphological detail is clearly discernible in each image. This particularly affects valve face texture whereby individual pores or raphe keel puncta were occasionally blurred in extended focus depth images, even though they were resolved in individual focus level images. It can be expected, however, that this situation can be improved by further optimization of the imaging workflow.

Direct transference of traditional taxonomic characters into numeric descriptors using image analysis, as attempted in this study, might prove unnecessary if the aim is simply automatic taxonomic identification. Recent work shows that a more generic approach, either based on an explicit separate feature extraction (Bueno et al. 2017), or using convolutional neural networks combining feature extraction and highly nonlinear classification into a single tool (Pedraza et al. 2017), might be just as successful or better, and more readily applicable to a broader set of taxa. This study, however, illustrates that using image analysis to quantify traditional characters used in diatom taxonomy and uni‐, bi‐ or multivariate analyses of such quantitative features, can aid the species delimitation process by making explicit fine patterns that are difficult to discern by observation (Figs. 6 and 7).

A final technical note is that the imaging workflow used previously (Kloster et al. 2017) and in this study can lead to duplicate images of individual specimens when multiple target valves lie in close proximity to each other on a slide. In these cases, such neighboring valves might end up being depicted in full in more than one image entering downstream analyses. At the time this study was initiated, this issue was not fully realized and there was no method available for automatically filtering out such duplicates. For transparency, in spite of having removed these duplicate images from our analyses, they were kept in Appendix S1, marked as duplicates. R code applicable for identifying such multiply imaged specimens automatically is now also available (Kloster et al. 2017). In spite of these drawbacks, automated diatom slide imaging procedures (Pech‐Pacheco and Cristóbal 2002, Kloster et al. 2017) are now coming close to a level of maturity for routine use. However, the everyday diatom analysis workflows will still require further rethinking to fully harvest the potential of these methods, not only for automatic identification but also for alpha taxonomy of diatoms. Our study takes a step in that direction.

## Conclusions

This study explored whether and how methods developed in the context of automatic identification and collaborative image identification could facilitate light microscopy‐based species delimitation in diatoms. It extends the so far most complete taxonomic characterization of the Southern Ocean diatom species *Fragilariopsis obliquecostata*,* F. ritscheri*, and *F. sublinearis* (Cefarelli et al. 2010) in the following ways: (i) by using automated methods supporting measurement, a larger number of specimens could be measured, substantially extending the ranges of basic morphometric characters; (ii) a series of characters considered taxonomically informative in the group but for which no quantification has been done previously were quantified using image analysis; (iii) by contrasting and reconciling the opinions of a number of experts and reflecting upon morphometric comparisons, a refined differential diagnosis was produced. We have demonstrated that an automatic identification of the three taxa with an accuracy comparable to human experts is possible. We propose that (i) highly standardized (semi)automated light microscopic imaging, (ii) web‐based multiexpert image identification, and (iii) algorithmic extraction of quantitative features designed after taxonomic characters, all have the potential for supporting diatom analysis.

Funding to Beszteri, Kloster and Kauer was provided by the DFG priority programme 1158 “Antarctic Research with comparative investigations in Arctic ice areas” under funding codes BE4316/4‐1 | KA1655/3‐1. Leventer was supported by NSF grant no. 1143836. Barcena was supported by University of Salamanca grant ID2014/0019, The Scientific Committee on Antarctic Research (PAIS and AnT‐ERA), The International Arctic Science Committee, The Palaeontological Association and The Micropalaeontological Society in the organization of the 5th Polar Marine Diatom Workshop.

## Supporting information


**Figure S1.** Validation of striae density measurement by SHERPA (on the *x*‐axis) versus measured manually (on the *y*‐axis). Black line: *y*=*x*. Red line: least squares regression line.

**Figure S2.** Dependence of identification agreement on apical valve length. The gray line represents the percentage of specimens within a 10 μm broad apical length range which received at least 90% identical taxonomic labels; the black dotted line depicts the absolute number of these cases within the 10 μm size window. The solid black line depicts the distribution of apical valve lengths in our test set of specimens (for comparability, also counted in 10 μm broad size windows). Note that although the *y*‐axis labeling is identical for the three curves, the scale is absolute for the black ones (black empty and filled circles) but refers to percentages for the gray line.

**Figure S3.** Relationship between valve width and apical length shows a much clearer separation than aspect ratio, and substantially less dependence on apical length.

**Figure S4.** Eccentricity of the broadest valve position along the apical axis hardly depends on apical length, and is slightly higher (away from 0.5 on the *y*‐axis) in *Fragilariopsis ritscheri* than in the other two species.

**Table S1.** Apical valve length ranges of the three species when considering (a) only specimens identified in full agreement (unequivocal); (b) specimens identified as belonging to the species considered by the majority of participants (majority); and (c) by any single participant (single vote).

**Table S2.** Valve width ranges of the three species when considering (a) only specimens identified in full agreement (unequivocal); (b) specimens identified as belonging to the species considered by the majority of participants (majority); and (c) by any single participant (single vote).

**Table S3.** Striae density ranges of the three species when considering (a) only specimens identified in full agreement (unequivocal); (b) specimens identified as belonging to the species considered by the majority of participants giving an identification for that specimen (majority); and (c) by any single participant (single vote).

**Appendix S1.** Table summarizing results of identifications by individual participants. The first five columns were provided to participants to enter their identifications. Columns N1‐N4 and E1‐E8 show the votes of individual participants which are summed up and summarized in the next eleven columns: NrVotes, total number of participants who gave an identification to the specimen image concerned; the next five columns count the numbers of votes falling into five categories (one for each species name, plus ambiguous and out‐of‐group votes); percentAgree, the proportion of participants voting for the category receiving the highest number of votes; MajorityVote codes the group receiving the highest number of votes (1, *ritscheri*; 2, *obliquecostata*; 3, *sublinearis*); tie indicates with one case where two or more categories received the same number of votes; ConsensusID gives the final identification used in the analyses. The remaining columns give free text remarks entered by the participants during their identifications.
