# Supplementary material for: Quantitative comparison of taxa and taxon concepts in the diatom genus Fragilariopsis: a case study on using slide scanning, multiexpert image annotation, and image analysis in taxonomy1
Source: J Phycol. 2018 Aug 28;54(5):703–19. doi: 10.1111/jpy.12767 (PMC6220827; doi:10.1111/jpy.12767)
Supplement: Supplementary file 1 — Figure S1. Validation of striae density measurement by SHERPA (on the x‐axis) versus measured manually (on the y‐axis). Black line: y=x. Red line: least squares regression line. [file JPY-54-703-s001.pdf]

Supplementary figure S1

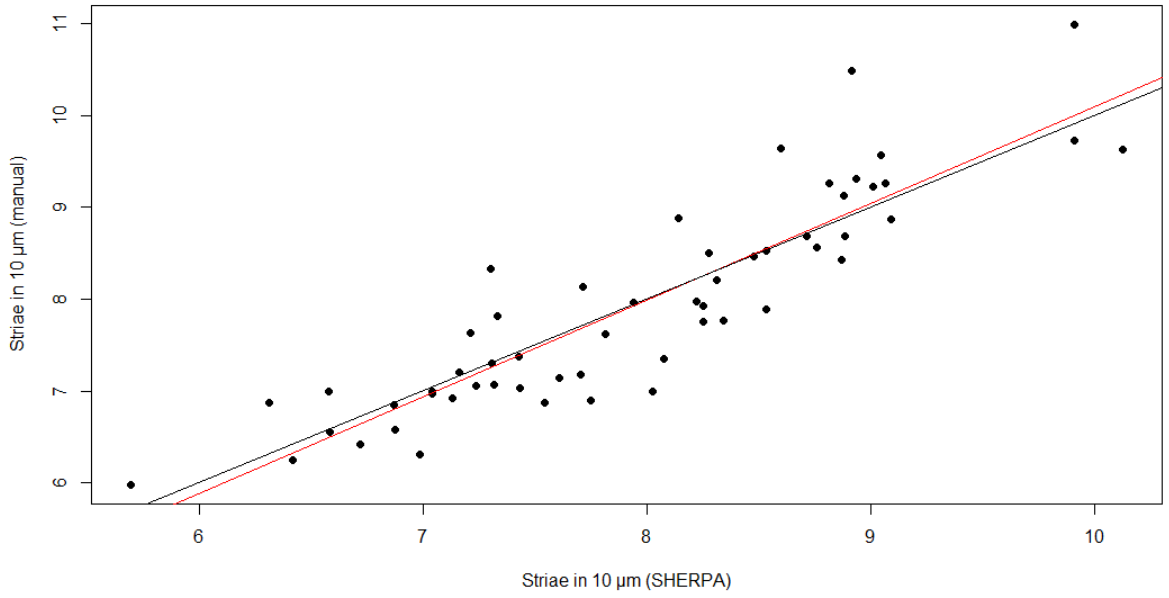

Supplementary Figure S1. Validation of striae density measurement by SHERPA (on the x axis) vs. measured manually (on the y axis). Black line: y=x. Red line: least squares regression line.
