# Supplementary material for: Quantitative comparison of taxa and taxon concepts in the diatom genus Fragilariopsis: a case study on using slide scanning, multiexpert image annotation, and image analysis in taxonomy1
Source: J Phycol. 2018 Aug 28;54(5):703–19. doi: 10.1111/jpy.12767 (PMC6220827; doi:10.1111/jpy.12767)
Supplement: Supplementary file 2 — Figure S2. Dependence of identification agreement on apical valve length. The gray line represents the percentage of specimens within a 10 μm broad apical length range which received at least 90% identical taxonomic labels; the black dotted line depicts the absolute number of these cases within the 10 μm size window. The solid black line depicts the distribution of apical valve lengths in our test set of specimens (for comparability, also counted in 10 μm broad size windows). Note that although the y‐axis labeling is identical for the three curves, the scale is absolute for the black ones (black empty and filled circles) but refers to percentages for the gray line. [file JPY-54-703-s002.pdf]

Supplementary figure S2

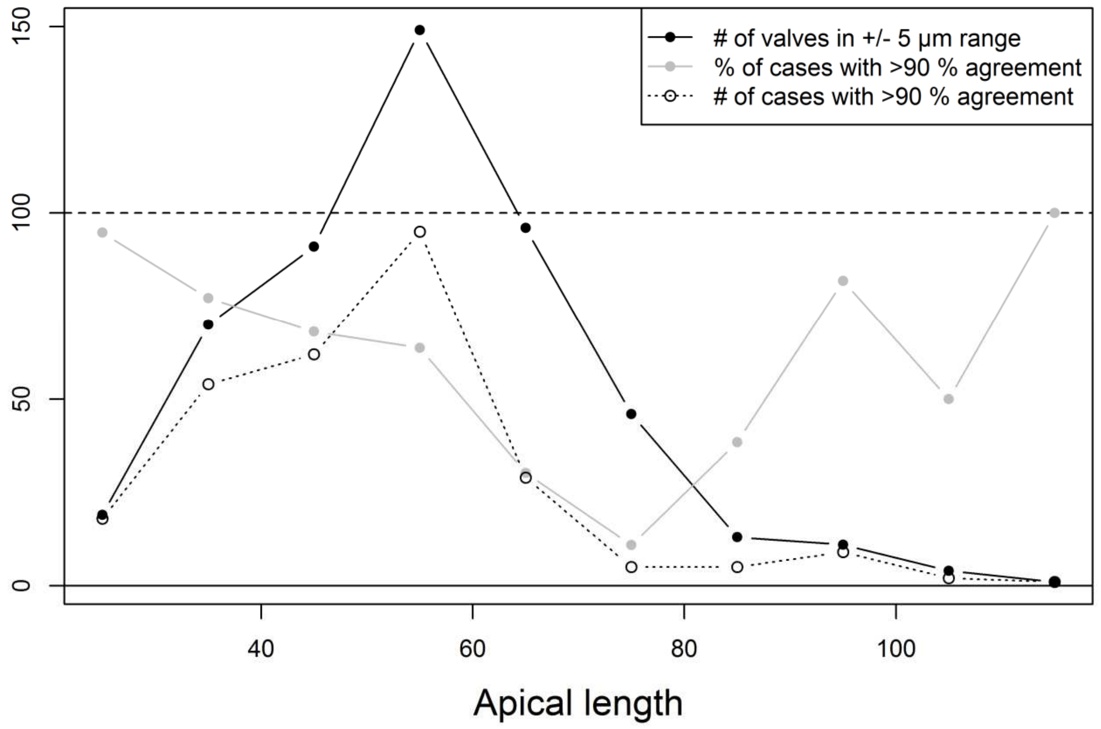

Supplementary Figure S2. Dependence of identification agreement on apical valve length. The gray line represents the percentage of specimens within a 10 µm broad apical length range which received at least 90 % identical taxonomic labels; the black dotted line depicts the absolute number of these cases within the 10 µm size window. The solid black line depicts the distribution of apical valve lengths in our test set of specimens (for comparability, also counted in 10 µm broad size windows). Note that although the y axis labelling is identical for the three curves, the scale is absolute for the black ones (black empty and filled circles) but refers to percentages for the gray line.
