# Supplementary material for: Quantitative comparison of taxa and taxon concepts in the diatom genus Fragilariopsis: a case study on using slide scanning, multiexpert image annotation, and image analysis in taxonomy1
Source: J Phycol. 2018 Aug 28;54(5):703–19. doi: 10.1111/jpy.12767 (PMC6220827; doi:10.1111/jpy.12767)
Supplement: Supplementary file 3 — Figure S3. Relationship between valve width and apical length shows a much clearer separation than aspect ratio, and substantially less dependence on apical length. [file JPY-54-703-s003.pdf]

Supplementary figure S3

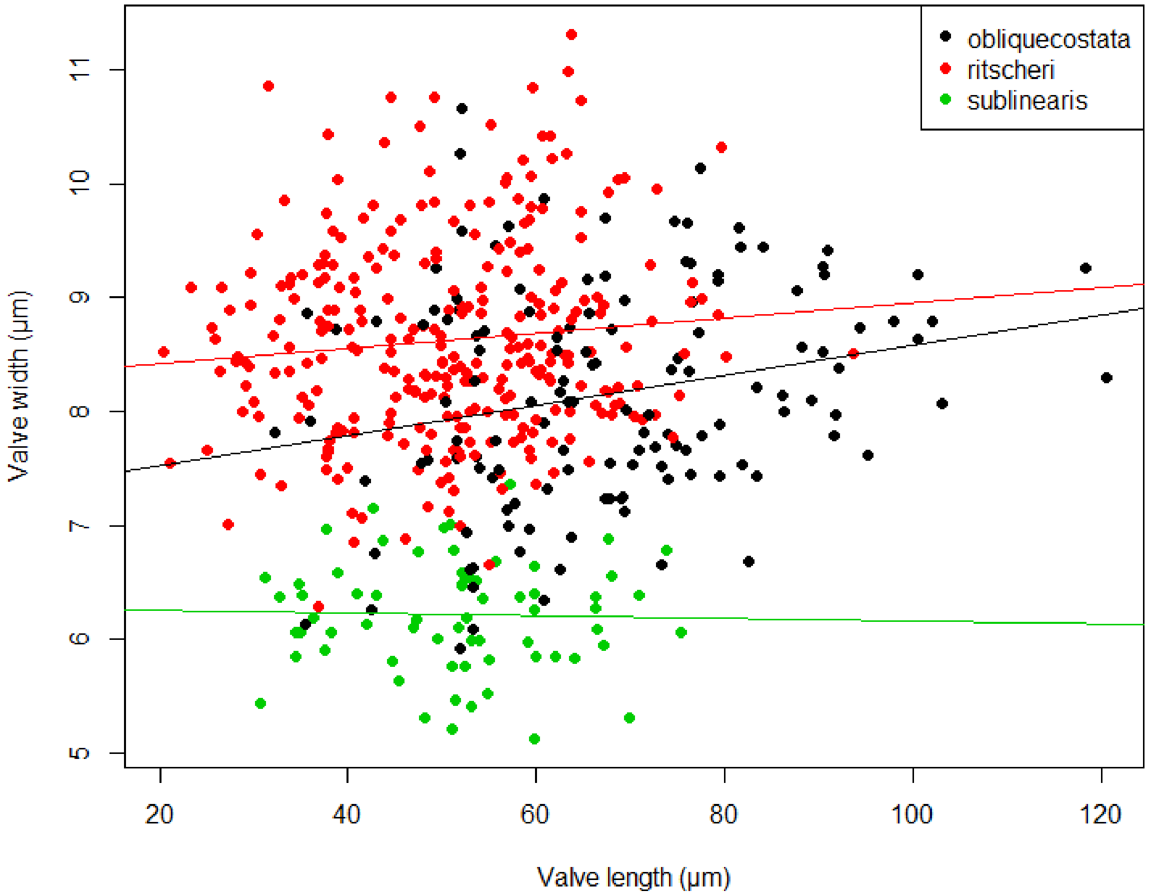

Supplementary Figure S3. Relationship between valve width and apical length shows a much clearer separation than aspect ratio, and substantially less dependence on apical length.
