# Supplementary material for: Quantitative comparison of taxa and taxon concepts in the diatom genus Fragilariopsis: a case study on using slide scanning, multiexpert image annotation, and image analysis in taxonomy1
Source: J Phycol. 2018 Aug 28;54(5):703–19. doi: 10.1111/jpy.12767 (PMC6220827; doi:10.1111/jpy.12767)
Supplement: Supplementary file 4 — Figure S4. Eccentricity of the broadest valve position along the apical axis hardly depends on apical length, and is slightly higher (away from 0.5 on the y‐axis) in Fragilariopsis ritscheri than in the other two species. [file JPY-54-703-s004.pdf]

Supplementary figure S4

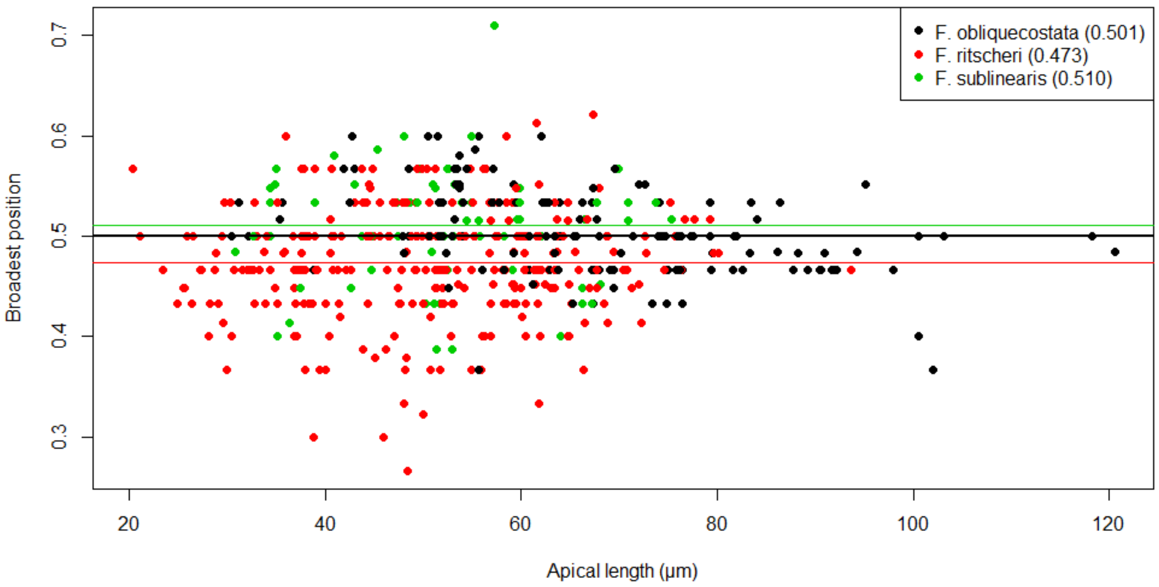

Supplementary Figure S4. Eccentricity of the broadest valve position along the apical axis hardly depends on apical length, and is slightly higher (away from 0.5 on the y-axis) in *F. ritscheri* than in the other two species.
