# Supplementary material for: Quantitative comparison of taxa and taxon concepts in the diatom genus Fragilariopsis: a case study on using slide scanning, multiexpert image annotation, and image analysis in taxonomy1
Source: J Phycol. 2018 Aug 28;54(5):703–19. doi: 10.1111/jpy.12767 (PMC6220827; doi:10.1111/jpy.12767)
Supplement: Supplementary file 5 — Table S1. Apical valve length ranges of the three species when considering (a) only specimens identified in full agreement (unequivocal); (b) specimens identified as belonging to the species considered by the majority of participants (majority); and (c) by any single participant (single vote). [file JPY-54-703-s005.pdf]

Supplementary material accompanying

QUANTITATIVE COMPARISON OF TAXA AND TAXON CONCEPTS IN THE DIATOM GENUS *FRAGILARIOPSIS*: A CASE STUDY ON USING SLIDE SCANNING, MULTI-EXPERT IMAGE ANNOTATION AND IMAGE ANALYSIS IN TAXONOMY

by Bánk Beszteri, Claire Allen, Gaston Almandoz, Leanne Armand, María Ángeles Barcena, Hannelore Cantzler, Xavier Crosta, Oliver Esper, Richard Jordan, Gerhard Kauer, Christine Klaas, Michael Kloster, Amy Leventer, Jennifer Pike and Andrés Rigual

Further supplementary data and code available at https://doi.org/10.1594/PANGAEA.879785

Supplementary table S1

	Valve length (apical axis, µm)					
	maximum			minimum		
	unequivocal	majority	single vote	unequivocal	majority	single vote
F. obliquecostata	118.3	120.5	120.5	60.8	32.2	28.9
F. ritscheri	57.6	93.7	103.1	20.3	20.3	20.3
F. sublinearis	66.3	75.3	120.5	48.1	30.7	30.5

Supplementary Table S1. Apical valve length ranges of the three species when considering a) only specimens identified in full agreement (unequivocal); b) specimens identified as belonging to the species considered by the majority of participants (majority); and c) by any single participant (single vote).
