# Supplementary material for: Quantitative comparison of taxa and taxon concepts in the diatom genus Fragilariopsis: a case study on using slide scanning, multiexpert image annotation, and image analysis in taxonomy1
Source: J Phycol. 2018 Aug 28;54(5):703–19. doi: 10.1111/jpy.12767 (PMC6220827; doi:10.1111/jpy.12767)
Supplement: Supplementary file 6 — Table S2. Valve width ranges of the three species when considering (a) only specimens identified in full agreement (unequivocal); (b) specimens identified as belonging to the species considered by the majority of participants (majority); and (c) by any single participant (single vote). [file JPY-54-703-s006.pdf]

Supplementary table S2

	Valve width (transapical axis, µm)					
	maximum			minimum		
	unequivocal	majority	single vote	unequivocal	majority	single vote
F. obliquecostata	9.3	10.7	11.3	7.8	5.9	5.1
F. ritscheri	10.8	11.3	11.3	7.1	6.3	5.4
F. sublinearis	6.7	7.4	10.7	5.1	5.1	5.1

Supplementary Table S2. Valve width ranges of the three species when considering a) only specimens identified in full agreement (unequivocal); b) specimens identified as belonging to the species considered by the majority of participants (majority); and c) by any single participant (single vote).
