# Supplementary material for: Quantitative comparison of taxa and taxon concepts in the diatom genus Fragilariopsis: a case study on using slide scanning, multiexpert image annotation, and image analysis in taxonomy1
Source: J Phycol. 2018 Aug 28;54(5):703–19. doi: 10.1111/jpy.12767 (PMC6220827; doi:10.1111/jpy.12767)
Supplement: Supplementary file 7 — Table S3. Striae density ranges of the three species when considering (a) only specimens identified in full agreement (unequivocal); (b) specimens identified as belonging to the species considered by the majority of participants giving an identification for that specimen (majority); and (c) by any single participant (single vote). [file JPY-54-703-s007.pdf]

Supplementary table S3

	Striae density (1 in 10 µm)					
	maximum			minimum		
	unequivocal	majority	single vote	unequivocal	majority	single vote
F. obliquecostata	7.3	9.6	10.4	5.7	4.7	4.7
F. ritscheri	9.8	11	11	5.6	5.2	4.9
F. sublinearis	10.3	10.5	10.5	7.4	6	4.7

Supplementary Table S3. Striae density ranges of the three species when considering a) only specimens identified in full agreement (unequivocal); b) specimens identified as belonging to the species considered by the majority of participantsgiving an identification for that specimen (majority); and c) by any single participant (single vote).
