# Supplementary material for: Quantitative comparison of taxa and taxon concepts in the diatom genus Fragilariopsis: a case study on using slide scanning, multiexpert image annotation, and image analysis in taxonomy1
Source: J Phycol. 2018 Aug 28;54(5):703–19. doi: 10.1111/jpy.12767 (PMC6220827; doi:10.1111/jpy.12767)
Supplement: Supplementary file 8 — Appendix S1. Table summarizing results of identifications by individual participants. The first five columns were provided to participants to enter their identifications. Columns N1‐N4 and E1‐E8 show the votes of individual participants which are summed up and summarized in the next eleven columns: NrVotes, total number of participants who gave an identification to the specimen image concerned; the next five columns count the numbers of votes falling into five categories (one for each species name, plus ambiguous and out‐of‐group votes); percentAgree, the proportion of participants voting for the category receiving the highest number of votes; MajorityVote codes the group receiving the highest number of votes (1, ritscheri; 2, obliquecostata; 3, sublinearis); tie indicates with one case where two or more categories received the same number of votes; ConsensusID gives the final identification used in the analyses. The remaining columns give free text remarks entered by the participants during their identifications. [file JPY-54-703-s008.pdf]

Categ	
	ritscheri
	obliquecostata
	sublinearis
	ambiguous
	nonTarget

ImageURL	ApicalLength	TransapicalWidth	StriaeIn10muSHERPA	StriaeIn10muManual	Image	N1	E1	E2	E3	E4	E5	N2	N3	E6	N4	E7	E8	NrVotes	votesForRitscheri	votesForObliquecostata	votesForSublinearis	votesAmbiguous	votesNonTarget	Nr. votes for winning name	percentAgree	MajorityVote	tie	ConsensusID	Remarks-N1	Remarks-E1	Remarks-E2	Remarks-E3	Remarks-E4	Remarks-E5	Remarks-N2	Remarks-N3	Remarks-E6	Remarks-N4	Remarks-E7	Remarks-E8
http://apps3.awi.de/Hustedt-Diatoms/FragCl/Set1/L020_W9.0-S9.3-PS1768-8.000190.png	20.3	8.5	9.3	NA	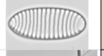	ritscheri	ritscheri	ritscheri	ritscheri	ritscheri	ritscheri	ritscheri	ritscheri	ritscheri	ritscheri	ritscheri	ritscheri	12	12	0	0	0	0	12	1	1	0	ritscheri												
http://apps3.awi.de/Hustedt-Diatoms/FragCl/Set1/L021_W8.0-S8.1-PS1768-8.000240.png	21.1	7.5	8.1	NA	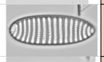	ritscheri	ritscheri	ritscheri	ritscheri	ritscheri	ritscheri	ritscheri	ritscheri	ritscheri	ritscheri	ritscheri	ritscheri	12	12	0	0	0	0	12	1	1	0	ritscheri												
http://apps3.awi.de/Hustedt-Diatoms/FragCl/Set1/L023_W9.0-SNA-PS1768-8.000127.png	23.4	9.1	NA	10.4	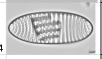	ritscheri	ritscheri	ritscheri	ritscheri	ritscheri	ritscheri	ritscheri	ritscheri	ambiguous	ritscheri	ritscheri	ritscheri	12	11	0	0	1	0	11	0.916	1	0	ritscheri												
http://apps3.awi.de/Hustedt-Diatoms/FragCl/Set1/L025_W8.0-S7.8-PS1768-8.000340.png	25.0	7.7	7.8	NA	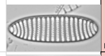	ritscheri	ritscheri	ritscheri	ritscheri	ritscheri	ritscheri	ritscheri	ritscheri	ritscheri	ritscheri	ritscheri	ritscheri	12	12	0	0	0	0	12	1	1	0	ritscheri												
http://apps3.awi.de/Hustedt-Diatoms/FragCl/Set1/L026_W9.0-S8.3-PS1768-8.000152.png	25.6	8.7	8.3	NA	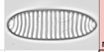	ritscheri	ritscheri	ritscheri	ritscheri	ritscheri	ritscheri	ritscheri	ritscheri	ritscheri	ritscheri	ritscheri	ritscheri	12	12	0	0	0	0	12	1	1	0	ritscheri												
http://apps3.awi.de/Hustedt-Diatoms/FragCl/Set1/L026_W9.0-S7.5-PS1768-8.000341.png	25.9	8.6	7.5	NA	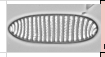	ritscheri	ritscheri	ritscheri	ritscheri	ritscheri	ritscheri	ritscheri	ritscheri	ritscheri	ritscheri	ritscheri	ritscheri	12	12	0	0	0	0	12	1	1	0	ritscheri												
http://apps3.awi.de/Hustedt-Diatoms/FragCl/Set1/L026_W8.0-S7.6-PS1768-8.000309.png	26.4	8.4	7.6	NA	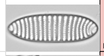	ritscheri	ritscheri	ritscheri	ritscheri	ritscheri	ritscheri	ritscheri	ritscheri	ritscheri	ritscheri	ritscheri	ritscheri	12	12	0	0	0	0	12	1	1	0	ritscheri												
http://apps3.awi.de/Hustedt-Diatoms/FragCl/Set1/L027_W9.0-S8.2-PS1768-8.000250.png	26.6	9.1	8.2	NA	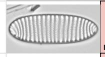	ritscheri	ritscheri	ritscheri	ritscheri	ritscheri	ritscheri	ritscheri	ritscheri	ritscheri	ritscheri	ritscheri	ritscheri	12	12	0	0	0	0	12	1	1	0	ritscheri												
http://apps3.awi.de/Hustedt-Diatoms/FragCl/Set1/L027_W7.0-S6.4-PS1768-8.000192.png	27.3	7.0	6.4	NA	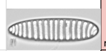	ritscheri	ritscheri	ritscheri	ritscheri	ritscheri	ritscheri	ritscheri	ritscheri	ritscheri	ritscheri	ritscheri	ambiguous	12	11	0	0	1	0	11	0.916	1	0	ritscheri				(3 rows of poroids)								XXXXXX: highly sil
http://apps3.awi.de/Hustedt-Diatoms/FragCl/Set1/L027_W9.0-S9.7-Hasle22-48.000002.png	27.4	8.9	9.7	NA	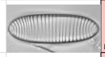	ritscheri	ritscheri	ritscheri	ritscheri	ritscheri	ritscheri	ritscheri	ritscheri	ritscheri	ritscheri	ritscheri	ritscheri	12	12	0	0	0	0	12	1	1	0	ritscheri												
http://apps3.awi.de/Hustedt-Diatoms/FragCl/Set1/L028_W8.0-S7.4-Hasle22-47.000013.png	28.1	8.4	7.4	NA	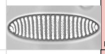	ritscheri	ritscheri	ritscheri	ritscheri	ritscheri	ritscheri	ritscheri	ritscheri	ritscheri	ritscheri	ritscheri	ritscheri	12	12	0	0	0	0	12	1	1	0	ritscheri												
http://apps3.awi.de/Hustedt-Diatoms/FragCl/Set1/L028_W8.0-SNA-FragCl.NBP-1402.960-961cm.000023.png	28.2	8.5	NA	8.8	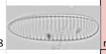	ritscheri	ritscheri	ritscheri	ritscheri	ritscheri	ritscheri	ritscheri	ritscheri	ritscheri	ritscheri	ritscheri		11	11	0	0	0	0	11	1	1	0	ritscheri												
http://apps3.awi.de/Hustedt-Diatoms/FragCl/Set1/L029_W8.0-S9.8-Hasle22-47.000034.png	28.7	8.0	9.8	NA	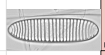	ritscheri	ritscheri	ritscheri	ritscheri	ritscheri	ritscheri	ritscheri	ritscheri	ritscheri	ritscheri	ritscheri	ritscheri	12	12	0	0	0	0	12	1	1	0	ritscheri												
http://apps3.awi.de/Hustedt-Diatoms/FragCl/Set1/L029_W8.0-S6.9-PS1768-8.000162.png	28.9	8.4	6.9	NA	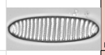	ritscheri	ritscheri	obliquecost	obliquecost	ritscheri	ritscheri	obliquecos	ritscheri	ambiguous	ritscheri	ritscheri	obliquecostata	12	7	4	0	1	0	7	0.583	1	0	ritscheri			seems isopolar to me, lanceolated, max transapical width mid way from				Not 100% sure: could also be ritscheri					
http://apps3.awi.de/Hustedt-Diatoms/FragCl/Set1/L029_W8.0-S8.5-PS1768-8.000402.png	29.1	8.2	8.5	NA	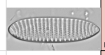	ritscheri	ritscheri	ritscheri	ritscheri	ritscheri	ritscheri	ritscheri	ritscheri	ritscheri	ritscheri	ritscheri	ritscheri	12	12	0	0	0	0	12	1	1	0	ritscheri												
http://apps3.awi.de/Hustedt-Diatoms/FragCl/Set1/L030_W8.0-S9.1-Hasle22-47.000010.png	29.5	8.4	9.1	NA	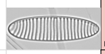	ritscheri	ritscheri	ritscheri	ritscheri	ritscheri	ritscheri	ritscheri	ritscheri	ritscheri	ritscheri	ritscheri	ritscheri	12	12	0	0	0	0	12	1	1	0	ritscheri												
http://apps3.awi.de/Hustedt-Diatoms/FragCl/Set1/L030_W9.0-S9.7-Hasle22-47.000011.png	29.6	8.9	9.7	NA	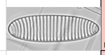	ritscheri	ritscheri	ritscheri	ritscheri	ritscheri	ritscheri	ritscheri	ritscheri	ritscheri	ritscheri	ritscheri	ritscheri	12	12	0	0	0	0	12	1	1	0	ritscheri												
http://apps3.awi.de/Hustedt-Diatoms/FragCl/Set1/L030_W9.0-S9.6-Wa-75b_Wa-77b.000082.png	29.7	9.2	9.6	NA	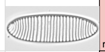	ritscheri	ritscheri	ritscheri	ritscheri	ritscheri	ritscheri	ritscheri	ritscheri	ritscheri	ritscheri	ritscheri	ritscheri	12	12	0	0	0	0	12	1	1	0	ritscheri												
http://apps3.awi.de/Hustedt-Diatoms/FragCl/Set1/L030_W8.0-S6.5-PS1768-8.000401.png	30.0	8.1	6.5	NA	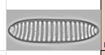	ritscheri	ritscheri	ritscheri	ritscheri	ritscheri	ritscheri	ritscheri	ritscheri	ritscheri	ritscheri	ritscheri	ritscheri	12	12	0	0	0	0	12	1	1	0	ritscheri												
http://apps3.awi.de/Hustedt-Diatoms/FragCl/Set1/L030_W10.0-S8.9-PS1768-8.000316.png	30.4	9.6	8.9	NA	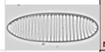	ritscheri	ritscheri	ritscheri	ritscheri	ritscheri	ritscheri	ritscheri	ritscheri	ritscheri	ritscheri	ritscheri	ritscheri	12	12	0	0	0	0	12	1	1	0	ritscheri												
http://apps3.awi.de/Hustedt-Diatoms/FragCl/Set1/L030_W8.0-S8.1-PS1768-8.000197.png	30.4	8.0	8.1	NA	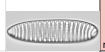	ritscheri	ritscheri	ritscheri	ritscheri	ritscheri	ritscheri	ritscheri	ritscheri	ritscheri	ritscheri	ritscheri	ritscheri	12	12	0	0	0	0	12	1	1	0	ritscheri												
http://apps3.awi.de/Hustedt-Diatoms/FragCl/Set1/L030_W7.0-S5.4-PS1768-8.000679.png	30.5	6.9	5.4	NA	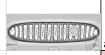	ambiguous	ambiguous	obliquecost	obliquecost	ritscheri	ritscheri	obliquecos	kerguelensis	ambiguous	sublinearis	ambiguous		11	2	3	1	4	1	4	0.363	4	0	ambiguous	?ritscherii/kergue	probably F. ritscheri, but much coarser striae			striae too wide? S	or very small obliquecostata- or non target specimen					indet: can´t be determined in only o	
http://apps3.awi.de/Hustedt-Diatoms/FragCl/Set1/L031_W7.0-S7.5-PS1768-8.000702.png	30.7	7.4	7.5	NA	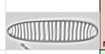	ritscheri	ritscheri	ritscheri	ritscheri	ritscheri	ritscheri	ritscheri	ritscheri	ritscheri	ritscheri	ritscheri	ritscheri	12	12	0	0	0	0	12	1	1	0	ritscheri												
http://apps3.awi.de/Hustedt-Diatoms/FragCl/Set1/L031_W5.0-S8.9-PS1768-8.000783.png	30.7	5.4	8.9	NA	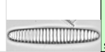	sublinearis	ambiguous	sublinearis	sublinearis	ritscheri	sublinearis	obliquecos	curta	ambiguous	sublinearis	sublinearis	sublinearis	12	1	1	7	2	1	7	0.583	3	0	sublinearis		probably F. sublinearis			ritscheri by default becasue I can't work out what else it could be - not s				sublinearis if marginal poroids were more distinct on both sides			
http://apps3.awi.de/Hustedt-Diatoms/FragCl/Set1/L031_W7.0-S8.1-PS1768-8.000523.png	31.2	6.5	8.1	NA	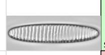	sublinearis	ambiguous	sublinearis	obliquecost	ritscheri	sublinearis	obliquecos	peragallii	ambiguous	obliquecostata	ambiguous	sublinearis	12	1	3	4	3	1	4	0.333	3	0	sublinearis		apical axis a little	even though it is a big wide, would		ritscheri by default becasue I can't work out what else it could be - not s				sublinearis if marginal poroids were		obliquecostata?	
http://apps3.awi.de/Hustedt-Diatoms/FragCl/Set1/L032_W11.0-S9.8-PS1768-8.000191.png	31.6	10.9	9.8	NA	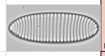	ritscheri	ritscheri	ritscheri	ritscheri	ritscheri	ritscheri	ritscheri	separanda	ritscheri	ritscheri	ritscheri	ritscheri	12	11	0	0	0	1	11	0.916	1	0	ritscheri												
http://apps3.awi.de/Hustedt-Diatoms/FragCl/Set1/L032_W9.0-S9.2-Hasle22-48.000001.png	32.0	8.7	9.2	NA	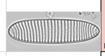	ritscheri	ritscheri	ritscheri	ritscheri	ritscheri	ritscheri	ritscheri	ritscheri	ritscheri	ritscheri	ritscheri	ritscheri	12	12	0	0	0	0	12	1	1	0	ritscheri												
http://apps3.awi.de/Hustedt-Diatoms/FragCl/Set1/L032_W8.0-S4.9-PS1768-8.000595.png	32.2	7.8	4.9	NA	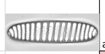	ambiguous	ritscheri	obliquecost	obliquecost	ritscheri	ritscheri	obliquecos	ritscheri	ambiguous	obliquecostata	obliquecostata	obliquecostata	12	4	6	0	2	0	6	0.5	2	0	obliquecostata	?ritscherii/kergue	oblique costae ar	seems isopolar to me, oblique costa		not confident - striae not right				probably obliquecostata			
http://apps3.awi.de/Hustedt-Diatoms/FragCl/Set1/L032_W8.0-SNA-FragCl.NBP-1402.960-961cm.000065.png	32.3	8.3	NA	6.9	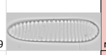	ritscheri	ritscheri	ritscheri	ritscheri	ritscheri	ritscheri	ritscheri	ritscheri	ritscheri	ritscheri	ambiguous	ritscheri	12	11	0	0	1	0	11	0.916	1	0	ritscheri		odd shape	weird one, very wedge like								indet: specimen not in focus	
http://apps3.awi.de/Hustedt-Diatoms/FragCl/Set1/L033_W9.0-S7.1-Hasle22-47.000012.png	32.7	8.8	7.1	NA	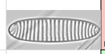	ritscheri	ritscheri	ritscheri	ritscheri	ritscheri	ritscheri	ritscheri	ritscheri	ritscheri	ritscheri	ritscheri	ritscheri	12	12	0	0	0	0	12	1	1	0	ritscheri												
http://apps3.awi.de/Hustedt-Diatoms/FragCl/Set1/L033_W6.0-S8.2-PS1768-8.000854.png	32.7	6.4	8.2	NA	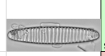	sublinearis	sublinearis	sublinearis	ambiguous	sublinearis	sublinearis	obliquecos	sublinearis	ambiguous	sublinearis	sublinearis	sublinearis	12	0	1	9	2	0	9	0.75	3	0	sublinearis				obliquecostata/su	ritscheri - sublineraris? gone with sublinearis because of poles				sublinearis if marginal poroids were more distinct on both sides			
http://apps3.awi.de/Hustedt-Diatoms/FragCl/Set1/L033_W9.0-S9.6-PS1768-8.000587.png	32.8	8.8	9.6	NA	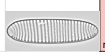	ritscheri	ritscheri	ritscheri	ritscheri	ritscheri	ritscheri		ritscheri	ritscheri	ritscheri	ritscheri	ritscheri	11	11	0	0	0	0	11	1	1	0	ritscheri												
http://apps3.awi.de/Hustedt-Diatoms/FragCl/Set1/L033_W9.0-S8.9-PS1768-8.000690.png	32.8	9.1	8.9	NA	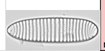	ritscheri	ritscheri	ritscheri	ritscheri	ritscheri	ritscheri		ritscheri	ritscheri	ritscheri	ritscheri	ritscheri	11	11	0	0	0	0	11	1	1	0	ritscheri												

http://apps3.awi.de/Hustedt-Diatoms/FragCl/Set1/L033_W7.0-S6.6-PS1768-8.000614.png	33.0	7.4	6.6	NA	ritscheri	ritscheri	ritscheri	ritscheri	ritscheri	ritscheri	ritscheri	ritscheri	ritscheri	ritscheri	ritscheri	ritscheri	12	12	0	0	0	0	12	1	1	0	ritscheri												
http://apps3.awi.de/Hustedt-Diatoms/FragCl/Set1/L033_W10.0-S7.5-PS1768-8.000280.png	33.3	9.9	7.5	NA	ritscheri	ritscheri	ritscheri	ritscheri	ritscheri	ritscheri	ritscheri	separanda	ambiguous	ritscheri	ritscheri	ritscheri	12	10	0	0	1	1	10	0.833	1	0	ritscheri									probably ritscheri but not sure			
http://apps3.awi.de/Hustedt-Diatoms/FragCl/Set1/L034_W9.0-S6.1-PS1768-8.000619.png	33.7	8.6	6.1	NA	ritscheri	ritscheri	ritscheri	ritscheri	ritscheri	ritscheri		ritscheri	ritscheri	ritscheri	ritscheri	ritscheri	11	11	0	0	0	0	11	1	1	0	ritscheri												
http://apps3.awi.de/Hustedt-Diatoms/FragCl/Set1/L034_W8.0-S6.7-PS1768-8.000285.png	33.8	8.4	6.7	NA	ritscheri	ritscheri	ritscheri	ritscheri	ritscheri	ritscheri		ritscheri	ritscheri	ritscheri	ritscheri	ritscheri	11	11	0	0	0	0	11	1	1	0	ritscheri												
http://apps3.awi.de/Hustedt-Diatoms/FragCl/Set1/L034_W9.0-S8.5-FragCl.ANT33-76.000014.png	33.8	9.1	8.5	NA	ritscheri	ritscheri	ritscheri	ritscheri	ritscheri	ritscheri		ritscheri	ritscheri	ritscheri	ritscheri	ritscheri	11	11	0	0	0	0	11	1	1	0	ritscheri												
http://apps3.awi.de/Hustedt-Diatoms/FragCl/Set1/L034_W9.0-S8.9-FragCl.ANT33-76.000006.png	34.0	9.2	8.9	NA	ritscheri	ritscheri	ritscheri	ritscheri	ritscheri	ritscheri		ritscheri	ritscheri	ritscheri	ritscheri	ritscheri	11	11	0	0	0	0	11	1	1	0	ritscheri												
http://apps3.awi.de/Hustedt-Diatoms/FragCl/Set1/L034_W9.0-S7.2-PS1768-8.000537.png	34.3	9.0	7.2	NA	ritscheri	ritscheri	ritscheri	ritscheri	ritscheri	ritscheri		ritscheri	ritscheri	ritscheri	ritscheri	ritscheri	11	11	0	0	0	0	11	1	1	0	ritscheri												
http://apps3.awi.de/Hustedt-Diatoms/FragCl/Set1/L034_W6.0-S6.9-FragCl.ANT33-100.000027.png	34.4	5.9	6.9	6.8	sublinearis	sublinearis	sublinearis	sublinearis	sublinearis	sublinearis		sublinearis	ambiguous	sublinearis	sublinearis	sublinearis	11	0	0	10	1	0	10	0.909	3	0	sublinearis					ritscheri - sublineraris? gone with sublinearis because of poles				sublinearis if marginal poroids were more distinct on both sides			
http://apps3.awi.de/Hustedt-Diatoms/FragCl/Set1/L034_W6.0-S8.9-FragCl.ANT33-100.000110.png	34.4	6.1	8.9	10.5	sublinearis	sublinearis	sublinearis	sublinearis	sublinearis	sublinearis		sublinearis	ambiguous	sublinearis	sublinearis	sublinearis	11	0	0	10	1	0	10	0.909	3	0	sublinearis					ritscheri - sublineraris? gone with sublinearis because of poles				sublinearis if marginal poroids were more distinct on both sides			
http://apps3.awi.de/Hustedt-Diatoms/FragCl/Set1/L035_W8.0-S6.8-PS1768-8.000577.png	34.8	7.9	6.8	NA	ritscheri	ritscheri	ritscheri	ritscheri	ritscheri	ritscheri		ritscheri	ritscheri	ritscheri	ritscheri	ritscheri	11	11	0	0	0	0	11	1	1	0	ritscheri												
http://apps3.awi.de/Hustedt-Diatoms/FragCl/Set1/L035_W6.0-S7.3-FragCl.ANT33-51.000124.png	34.9	6.5	7.3	7.8	sublinearis	sublinearis	sublinearis	sublinearis	sublinearis	sublinearis		sublinearis	ambiguous	sublinearis	sublinearis	sublinearis	11	0	0	10	1	0	10	0.909	3	0	sublinearis					ritscheri - sublineraris? gone with sublinearis because of poles				sublinearis if marginal poroids were more distinct on both sides			
http://apps3.awi.de/Hustedt-Diatoms/FragCl/Set1/L035_W6.0-S8.1-FragCl.ANT33-100.000086.png	35.0	6.1	8.1	8.9	sublinearis	sublinearis	sublinearis	sublinearis	sublinearis	sublinearis		sublinearis	ambiguous	sublinearis	sublinearis	sublinearis	11	0	0	10	1	0	10	0.909	3	0	sublinearis					ritscheri - sublineraris? gone with sublinearis because of poles				sublinearis if marginal poroids were more distinct on both sides			
http://apps3.awi.de/Hustedt-Diatoms/FragCl/Set1/L035_W8.0-S7.6-Hasle22-47.000026.png	35.1	8.1	7.6	NA	ritscheri	ritscheri	ritscheri	ritscheri	ritscheri	ritscheri		ritscheri	ritscheri	ritscheri	ritscheri	ritscheri	11	11	0	0	0	0	11	1	1	0	ritscheri												
http://apps3.awi.de/Hustedt-Diatoms/FragCl/Set1/L035_W6.0-S7.9-PS1768-8.000513.png	35.1	6.4	7.9	NA	sublinearis	sublinearis	sublinearis	sublinearis	sublinearis	sublinearis		sublinearis	ambiguous	sublinearis	sublinearis	sublinearis	11	0	0	10	1	0	10	0.909	3	0	sublinearis					ritscheri - sublineraris? gone with sublinearis because of poles				sublinearis if marginal poroids were more distinct on both sides			
http://apps3.awi.de/Hustedt-Diatoms/FragCl/Set1/L035_W9.0-S8.5-Hasle22-48.000003.png	35.2	9.2	8.5	NA	ritscheri	ritscheri	ritscheri	ritscheri	ritscheri	ritscheri		ritscheri	ritscheri	ritscheri	ritscheri	ritscheri	11	11	0	0	0	0	11	1	1	0	ritscheri												
http://apps3.awi.de/Hustedt-Diatoms/FragCl/Set1/L035_W6.0-S5.4-PS1768-8.000789.png	35.4	6.1	5.4	NA	ambiguous	ambiguous	obliquecost	obliquecost	ritscheri	obliquecostata		kerguelensis	ambiguous	sublinearis	obliquecostata		10	1	4	1	3	1	4	0.4	2	0	obliquecostata	?sublinearis/kerg	deformed specim	isopolar, I have n	Curved valve (ver	striae too wide? S	very small form, or ritscheri abberant			probably obliquecostata			
http://apps3.awi.de/Hustedt-Diatoms/FragCl/Set1/L036_W9.0-S6.6-Hasle22-40.000013.png	35.6	8.9	6.6	NA	ritscheri	ritscheri		obliquecost	ritscheri	obliquecost	obliquecos	ritscheri	ambiguous	obliquecostata	obliquecostata	obliquecostata	11	4	6	0	1	0	6	0.545	2	0	obliquecostata		very oblique stria	out of focus, not sure what it is. Isop		very slanted stria	could be confused with ritscherii, but not heteropolar			probably obliquecostata			isopolar?
http://apps3.awi.de/Hustedt-Diatoms/FragCl/Set1/L036_W8.0-S8.2-Hasle22-47.000029.png	35.7	8.4	8.2	NA	ritscheri	ritscheri	ritscheri	ritscheri	ritscheri	ritscheri	ritscheri	ritscheri	ritscheri	ritscheri	ritscheri	ritscheri	12	12	0	0	0	0	12	1	1	0	ritscheri												
http://apps3.awi.de/Hustedt-Diatoms/FragCl/Set1/L036_W8.0-S7.6-Wa-75b_Wa-77b.000039.png	35.8	8.1	7.6	NA	ritscheri	ritscheri	ritscheri	ritscheri	ritscheri	ritscheri	ritscheri	ritscheri	ritscheri	ritscheri	ritscheri	ritscheri	12	12	0	0	0	0	12	1	1	0	ritscheri										In this picture is hard to discern the terminal costae th		
http://apps3.awi.de/Hustedt-Diatoms/FragCl/Set1/L036_W8.0-S6.1-PS1768-8.000659.png	36.0	7.9	6.1	NA	ritscheri	ritscheri	obliquecost	obliquecost	ritscheri	obliquecost	obliquecos	ritscheri	ambiguous	obliquecostata	ritscheri	obliquecostata	12	5	6	0	1	0	6	0.5	2	0	obliquecostata			seems isopolar to	Hard to decide (it looks slightly hete		very small form, or ritscheri abberant			probably obliquecostata			
http://apps3.awi.de/Hustedt-Diatoms/FragCl/Set1/L036_W6.0-S8.3-PS1768-8.000074.png	36.3	6.2	8.3	NA	sublinearis	sublinearis	sublinearis	sublinearis	ritscheri	sublinearis		sublinearis	ambiguous	obliquecostata	sublinearis	sublinearis	11	1	1	8	1	0	8	0.727	3	0	sublinearis						abberant form- weakly heteropolar, slightly oblique c			sublinearis if marginal poroids were more distinct on both sides			
http://apps3.awi.de/Hustedt-Diatoms/FragCl/Set1/L037_W8.0-S8.1-Hasle22-47.000032.png	36.7	8.2	8.1	NA	ritscheri	ritscheri	ritscheri	ritscheri	ritscheri	ritscheri	ritscheri	ritscheri	ritscheri	ritscheri	ritscheri	ritscheri	12	12	0	0	0	0	12	1	1	0	ritscheri												
http://apps3.awi.de/Hustedt-Diatoms/FragCl/Set1/L037_W9.0-S7.4-PS1768-8.000308.png	36.8	9.3	7.4	NA	ritscheri	ritscheri	ritscheri	ritscheri	ritscheri	ritscheri	ritscheri	ritscheri	ritscheri	ritscheri	ritscheri	ritscheri	12	12	0	0	0	0	12	1	1	0	ritscheri												
http://apps3.awi.de/Hustedt-Diatoms/FragCl/Set1/L037_W6.0-S5.9-PS1768-8.000183.png	36.8	6.3	5.9	NA	sublinearis	ritscheri	ritscheri	ritscheri	ritscheri	ritscheri	ritscheri	ritscheri	ritscheri	ritscheri	ambiguous	ritscheri	12	10	0	1	1	0	10	0.833	1	0	ritscheri		transapcial axis a little narrower than average									obliquecostata?: costae oblique, bu	
http://apps3.awi.de/Hustedt-Diatoms/FragCl/Set1/L037_W9.0-S7.8-PS1768-8.000096.png	36.8	9.1	7.8	NA	ritscheri	ritscheri	ritscheri	ritscheri	ritscheri	ritscheri	ritscheri	ritscheri	ritscheri	ritscheri	ritscheri	ritscheri	12	12	0	0	0	0	12	1	1	0	ritscheri												
http://apps3.awi.de/Hustedt-Diatoms/FragCl/Set1/L037_W9.0-S7.0-PS1768-8.000848.png	36.9	8.8	7.0	NA	ritscheri	ritscheri	ritscheri	ritscheri	ritscheri	ritscheri	ritscheri	ritscheri	ritscheri	sublinearis	ritscheri	ritscheri	12	11	0	1	0	0	11	0.916	1	0	ritscheri												
http://apps3.awi.de/Hustedt-Diatoms/FragCl/Set1/L037_W8.0-S9.2-Hasle22-47.000031.png	37.1	8.5	9.2	NA	ritscheri	ritscheri	ritscheri	ritscheri	ritscheri	ritscheri	ritscheri	ritscheri	ritscheri	ritscheri	ritscheri	ritscheri	12	12	0	0	0	0	12	1	1	0	ritscheri												
http://apps3.awi.de/Hustedt-Diatoms/FragCl/Set1/L037_W9.0-S7.6-Hasle22-47.000028.png	37.2	8.7	7.6	NA	ritscheri	ritscheri	ritscheri	ritscheri	ritscheri	ritscheri	ritscheri	ritscheri	ritscheri	ritscheri	ritscheri	ritscheri	12	12	0	0	0	0	12	1	1	0	ritscheri												
http://apps3.awi.de/Hustedt-Diatoms/FragCl/Set1/L037_W9.0-S9.5-PS1768-8.000133.png	37.3	9.3	9.5	NA	ritscheri	ritscheri	ritscheri	ritscheri	ritscheri	ritscheri	ritscheri	ritscheri	ritscheri	ritscheri	ritscheri	ritscheri	12	12	0	0	0	0	12	1	1	0	ritscheri												
http://apps3.awi.de/Hustedt-Diatoms/FragCl/Set1/L037_W6.0-S7.9-PS1768-8.000855.png	37.4	5.9	7.9	NA	sublinearis	ambiguous			sublinearis	sublinearis	obliquecos	sublinearis	ambiguous	obliquecostata	sublinearis		9	0	2	5	2	0	5	0.555	3	0	sublinearis			tilted wiew. Mayb	I am not sure... It	difficult photo - guess sublinearis				sublinearis if marginal poroids were more distinct on b			out of focus
http://apps3.awi.de/Hustedt-Diatoms/FragCl/Set1/L037_W9.0-S8.2-PS1768-8.000144.png	37.5	9.4	8.2	NA	ritscheri	ritscheri	ritscheri	ritscheri	ritscheri	ritscheri	ritscheri	ritscheri	ritscheri	ritscheri	ritscheri	ritscheri	12	12	0	0	0	0	12	1	1	0	ritscheri												
http://apps3.awi.de/Hustedt-Diatoms/FragCl/Set1/L038_W9.0-SNA-PS1768-8.000259.png	37.5	9.2	NA	7.2	ritscheri	ritscheri	ritscheri	ritscheri	ritscheri	ritscheri		ritscheri	ritscheri	ritscheri	ritscheri	ritscheri	11	11	0	0	0	0	11	1	1	0	ritscheri												
http://apps3.awi.de/Hustedt-Diatoms/FragCl/Set1/L038_W7.0-S5.9-PS1768-8.000579.png	37.6	7.5	5.9	NA	ambiguous	ritscheri	ritscheri	ritscheri	ritscheri	ritscheri	ritscheri	ritscheri	ritscheri	ritscheri	ritscheri	ritscheri	12	11	0	0	1	0	11	0.916	1	0	ritscheri	?ritscherii/kerguelensis											
http://apps3.awi.de/Hustedt-Diatoms/FragCl/Set1/L038_W10.0-S8.7-Hasle22-48.000004.png	37.6	9.7	8.7	NA	ritscheri	ritscheri	ritscheri	ritscheri	ritscheri	ritscheri	ritscheri	ritscheri	ritscheri	ritscheri	ritscheri	ritscheri	12	12	0	0	0	0	12	1	1	0	ritscheri												
http://apps3.awi.de/Hustedt-Diatoms/FragCl/Set1/L038_W7.0-SNA-FragCl.ANT33-51.000380.png	37.6	7.0	NA	9.5	ambiguous	sublinearis	sublinearis		ritscheri	sublinearis	obliquecos	angulata	ambiguous	sublinearis	sublinearis	sublinearis	11	1	1	6	2	1	6	0.545	3	0	sublinearis	?sublinearis/ritscherii		a large one, but k	I am not sure... (n	difficult photo				sublinearis if marg	Not a clear pictur	specimen not in focus	
http://apps3.awi.de/Hustedt-Diatoms/FragCl/Set1/L038_W8.0-S6.8-PS1768-8.000497.png	37.8	7.6	6.8	NA	ritscheri	ritscheri	ritscheri	ritscheri	ritscheri	ritscheri		ritscheri	ritscheri	ritscheri	ambiguous	ritscheri	11	10	0	0	1	0	10	0.909	1	0	ritscheri									poss kerguelensis - if areolae patter		ritscheri?: specimen isopolar	
http://apps3.awi.de/Hustedt-Diatoms/FragCl/Set1/L038_W8.0-S7.4-PS1768-8.000588.png	37.8	7.7	7.4	NA	ritscheri	ritscheri	ritscheri	ritscheri	ritscheri	ritscheri		ritscheri	ritscheri	ritscheri	ritscheri	ritscheri	11	11	0	0	0	0	11	1	1	0	ritscheri												

http://apps3.awi.de/Hustedt-Diatoms/FragCl/Set1/L038_W8.0-S6.7-FragCl.NBP-1402.945-946cm.000032.png	37.8	7.6	6.7	NA	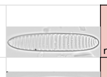	ritscheri	ritscheri	ritscheri	ritscheri	ritscheri	ritscheri		ritscheri	ritscheri	ritscheri	ritscheri	ritscheri	11	11	0	0	0	0	11	1	1	0	ritscheri												
http://apps3.awi.de/Hustedt-Diatoms/FragCl/Set1/L038_W10.0-S8.8-PS1768-8.000653.png	37.8	10.4	8.8	NA	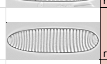	ritscheri	ritscheri	ritscheri	ritscheri	ritscheri	ritscheri	ritscheri	ritscheri	ritscheri	ritscheri	ritscheri	ritscheri	12	12	0	0	0	0	12	1	1	0	ritscheri												
http://apps3.awi.de/Hustedt-Diatoms/FragCl/Set1/L038_W9.0-S9.0-PS1768-8.000699.png	37.9	8.8	9.0	NA	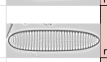	ritscheri	ritscheri	ritscheri	ritscheri	ritscheri	ritscheri		ritscheri	ritscheri	ritscheri	ritscheri	ritscheri	11	11	0	0	0	0	11	1	1	0	ritscheri												
http://apps3.awi.de/Hustedt-Diatoms/FragCl/Set1/L038_W9.0-S9.0-FragCl.NBP-1402.960-961cm.000056.png	37.9	8.9	9.0	NA	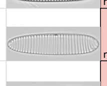	ritscheri	ritscheri	ritscheri	ritscheri	ritscheri	ritscheri		ritscheri	ritscheri	ritscheri	ritscheri	ritscheri	11	11	0	0	0	0	11	1	1	0	ritscheri												
http://apps3.awi.de/Hustedt-Diatoms/FragCl/Set1/L038_W8.0-S6.7-PS1768-8.000869.png	38.0	7.7	6.7	NA	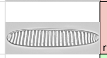	ritscheri	ritscheri	ritscheri	ritscheri	ritscheri	ritscheri		ritscheri	ritscheri	obliquecostata	ritscheri	ritscheri	11	10	1	0	0	0	10	0.909	1	0	ritscheri												
http://apps3.awi.de/Hustedt-Diatoms/FragCl/Set1/L038_W6.0-S8.3-FragCl.ANT33-51.000115.png	38.2	6.1	8.3	8.5	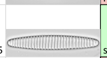	sublinearis	sublinearis	sublinearis	ambiguous	sublinearis	sublinearis	obliquecos	sublinearis	sublinearis	obliquecostata	sublinearis	sublinearis	12	0	2	9	1	0	9	0.75	3	0	sublinearis				obliquecostata/sublinearis								
http://apps3.awi.de/Hustedt-Diatoms/FragCl/Set1/L038_W10.0-S9.8-PS1768-8.000137.png	38.3	9.6	9.8	NA	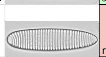	ritscheri	ritscheri	ritscheri	ritscheri	ritscheri	ritscheri	ritscheri	ritscheri	ritscheri	ritscheri	ritscheri	ritscheri	12	12	0	0	0	0	12	1	1	0	ritscheri												
http://apps3.awi.de/Hustedt-Diatoms/FragCl/Set1/L038_W9.0-S7.5-Hasle22-47.000027.png	38.3	9.3	7.5	NA	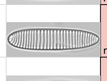	ritscheri	ritscheri	ritscheri	ritscheri	ritscheri	ritscheri		ritscheri	ritscheri	ritscheri	ritscheri	ritscheri	11	11	0	0	0	0	11	1	1	0	ritscheri												
http://apps3.awi.de/Hustedt-Diatoms/FragCl/Set1/L039_W9.0-S9.9-FragCl.ANT33-100.000071.png	38.6	8.9	9.9	9.7	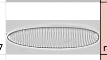	ritscheri	ritscheri	ritscheri	ritscheri	ritscheri	ritscheri		ritscheri	ritscheri	ritscheri	ritscheri	ritscheri	11	11	0	0	0	0	11	1	1	0	ritscheri						could be confused with sublinearis- went with fact that I could seethe poroids and the width/length of valve and slight hetropo						
http://apps3.awi.de/Hustedt-Diatoms/FragCl/Set1/L039_W8.0-S7.3-PS1768-8.000608.png	38.7	7.8	7.3	NA	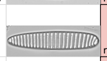	ritscheri	ritscheri	ritscheri	ritscheri	ritscheri	ritscheri		ritscheri	ritscheri	ritscheri	ritscheri	ritscheri	11	11	0	0	0	0	11	1	1	0	ritscheri												
http://apps3.awi.de/Hustedt-Diatoms/FragCl/Set1/L039_W9.0-S6.9-PS1768-8.000529.png	38.8	8.7	6.9	NA	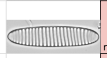	ritscheri	ritscheri	obliquecost	obliquecost	ritscheri	obliquecostata		angulata	obliquecostata	obliquecostata	ambiguous	obliquecostata	11	3	6	0	1	1	6	0.545	2	0	obliquecostata						could be confused with ritscherii, but weakly heteropolar and costae oblique, otherwise si					ritscheri?: specimen not in focus	
http://apps3.awi.de/Hustedt-Diatoms/FragCl/Set1/L039_W7.0-S7.4-FragCl.NBP-1402.945-946cm.000083.png	38.8	7.4	7.4	NA	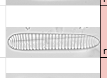	ritscheri	ambiguous	ritscheri	ritscheri	ritscheri	ritscheri		ritscheri	ritscheri	ritscheri	ritscheri	ritscheri	11	10	0	0	1	0	10	0.909	1	0	ritscheri		probably F. ritsch	again, never saw this wedgy shape I		not typical because of pinched valve centre							
http://apps3.awi.de/Hustedt-Diatoms/FragCl/Set1/L039_W10.0-S7.1-PS1768-8.000120.png	38.9	10.0	7.1	NA	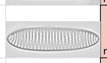	ritscheri	ritscheri	ritscheri	ritscheri	ritscheri	ritscheri		ritscheri	ritscheri	ritscheri	ritscheri	ritscheri	11	11	0	0	0	0	11	1	1	0	ritscheri												
http://apps3.awi.de/Hustedt-Diatoms/FragCl/Set1/L039_W8.0-S6.1-Wa-75b_Wa-77b.000167.png	38.9	7.9	6.1	NA	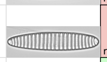	ritscheri	ritscheri	ritscheri	ritscheri	ritscheri	ritscheri		ritscheri	ritscheri	ritscheri	ritscheri	ritscheri	11	11	0	0	0	0	11	1	1	0	ritscheri												
http://apps3.awi.de/Hustedt-Diatoms/FragCl/Set1/L039_W7.0-S6.1-PS1768-8.000151.png	38.9	6.6	6.1	NA	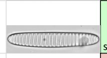	sublinearis	ambiguous	sublinearis	obliquecost	ritscheri	ritscheri		sublinearis	ambiguous	sublinearis	sublinearis	obliquecostata	11	2	2	5	2	0	5	0.454	3	0	sublinearis		deformed			difficult - hint of s	abberant form- weakly heteropolar, oblique costae, c			probably obliquecostata			isopolar?
http://apps3.awi.de/Hustedt-Diatoms/FragCl/Set1/L039_W9.0-S8.6-PS1768-8.000788.png	39.0	9.1	8.6	NA	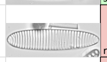	ritscheri	ritscheri	ritscheri	ritscheri	ritscheri	ritscheri		ritscheri	ritscheri	ritscheri	ritscheri	ritscheri	11	11	0	0	0	0	11	1	1	0	ritscheri												
http://apps3.awi.de/Hustedt-Diatoms/FragCl/Set1/L039_W10.0-S6.8-PS1768-8.000543.png	39.2	9.5	6.8	NA	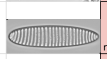	ritscheri	ritscheri	ritscheri	ritscheri	ritscheri	ritscheri		ritscheri	ritscheri	ritscheri	ritscheri	ritscheri	11	11	0	0	0	0	11	1	1	0	ritscheri												
http://apps3.awi.de/Hustedt-Diatoms/FragCl/Set1/L039_W8.0-S7.5-FragCl.NBP-1402.960-961cm.000060.png	39.4	7.8	7.5	NA	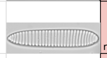	ritscheri	ritscheri	ritscheri	ritscheri	ritscheri	ritscheri		ritscheri	ritscheri	ritscheri	ritscheri	ritscheri	11	11	0	0	0	0	11	1	1	0	ritscheri												
http://apps3.awi.de/Hustedt-Diatoms/FragCl/Set1/L040_W8.0-SNA-FragCl.NBP-1402.945-946cm.000064.png	40.0	7.5	NA	7.1	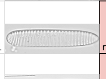	ritscheri	ritscheri	ritscheri	ritscheri	ritscheri	ritscheri	ritscheri	ritscheri	ritscheri	ritscheri	ritscheri	ritscheri	12	12	0	0	0	0	12	1	1	0	ritscheri		large end a little blunter than average										
http://apps3.awi.de/Hustedt-Diatoms/FragCl/Set1/L040_W9.0-S8.6-Wa-75b_Wa-77b.000173.png	40.0	8.7	8.6	NA	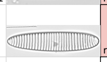	ritscheri	ritscheri	ritscheri	ritscheri	ritscheri	ritscheri	ritscheri	ritscheri	ritscheri	ritscheri	ritscheri	ritscheri	12	12	0	0	0	0	12	1	1	0	ritscheri												
http://apps3.awi.de/Hustedt-Diatoms/FragCl/Set1/L040_W7.0-S6.3-PS1768-8.000526.png	40.4	7.1	6.3	NA	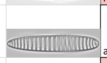	ambiguous	ritscheri	ritscheri	ritscheri	ritscheri	ritscheri	ritscheri	ritscheri	ambiguous	ritscheri	ritscheri	ritscheri	12	10	0	0	2	0	10	0.833	1	0	ritscheri	?ritscherii/kergue	striae a little wider than average							ritscheri or obliquecostata			highly silicified
http://apps3.awi.de/Hustedt-Diatoms/FragCl/Set1/L040_W9.0-SNA-Hasle22-47.000003.png	40.5	8.6	NA	8.6	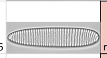	ritscheri	ritscheri	ritscheri	ritscheri	ritscheri	ritscheri	ritscheri	ritscheri	ritscheri	ritscheri	ritscheri	ritscheri	12	12	0	0	0	0	12	1	1	0	ritscheri												
http://apps3.awi.de/Hustedt-Diatoms/FragCl/Set1/L041_W9.0-S6.0-PS1768-8.000500.png	40.5	9.2	6.0	NA	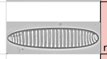	ritscheri	ritscheri	ritscheri	ritscheri	ritscheri	ritscheri	ritscheri	ritscheri	ritscheri	ritscheri	ritscheri	ritscheri	12	12	0	0	0	0	12	1	1	0	ritscheri												
http://apps3.awi.de/Hustedt-Diatoms/FragCl/Set1/L041_W8.0-S7.0-PS1768-8.000470.png	40.6	7.8	7.0	NA	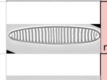	ritscheri	ritscheri	ritscheri	ritscheri	ritscheri	ritscheri	ritscheri	ritscheri	ambiguous	ritscheri	ritscheri	ritscheri	12	11	0	0	1	0	11	0.916	1	0	ritscheri									ritscheri or obliquecostata			
http://apps3.awi.de/Hustedt-Diatoms/FragCl/Set1/L041_W8.0-S7.0-PS1768-8.000485.png	40.6	7.9	7.0	NA	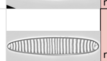	ritscheri	ritscheri	ritscheri	ritscheri	ritscheri	ritscheri	ritscheri	ritscheri	ambiguous	ritscheri	ritscheri	ritscheri	12	11	0	0	1	0	11	0.916	1	0	ritscheri									ritscheri or obliquecostata			
http://apps3.awi.de/Hustedt-Diatoms/FragCl/Set1/L041_W7.0-S7.0-PS1768-8.000467.png	40.6	6.8	7.0	NA	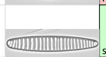	sublinearis	ambiguous	ritscheri	ritscheri	ritscheri	ritscheri	ritscheri	ritscheri	ambiguous	ritscheri	ritscheri	ritscheri	12	9	0	1	2	0	9	0.75	1	0	ritscheri		probably F. ritscheri, a little narrower transapical axis							ritscheri or obliquecostata			
http://apps3.awi.de/Hustedt-Diatoms/FragCl/Set1/L041_W9.0-S7.4-FragCl.ANT33-100.000062.png	40.7	8.7	7.4	7.4	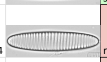	ritscheri	ritscheri	obliquecost	obliquecost	ritscheri	ritscheri	obliquecos	angulata	ambiguous	obliquecostata	obliquecostata	ritscheri	12	5	5	0	1	1	5	0.416	1	1	ambiguous			tricky one, I would need several focus plans to be sure			could be confused with sublinearis- went with fact tha			ritscheri or obliquecostata			
http://apps3.awi.de/Hustedt-Diatoms/FragCl/Set1/L041_W9.0-S9.2-Hasle22-48.000007.png	40.8	9.1	9.2	NA	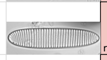	ritscheri	ritscheri	ritscheri	ritscheri	ritscheri	ritscheri	ritscheri	ritscheri	ritscheri	ritscheri	ritscheri	ritscheri	12	12	0	0	0	0	12	1	1	0	ritscheri												
http://apps3.awi.de/Hustedt-Diatoms/FragCl/Set1/L041_W9.0-S7.0-Hasle22-47.000001.png	40.9	8.5	7.0	NA	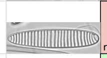	ritscheri	ritscheri	ritscheri	ritscheri	ritscheri	ritscheri	ritscheri	ritscheri	ritscheri	ritscheri	ritscheri	ritscheri	12	12	0	0	0	0	12	1	1	0	ritscheri												
http://apps3.awi.de/Hustedt-Diatoms/FragCl/Set1/L041_W6.0-S8.9-FragCl.ANT33-51.000118.png	40.9	6.4	8.9	8.7	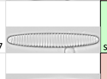	sublinearis	sublinearis	sublinearis	sublinearis	sublinearis	sublinearis	obliquecos	sublinearis	sublinearis	sublinearis	sublinearis	sublinearis	12	0	1	11	0	0	11	0.916	3	0	sublinearis					because not heteropolar							
http://apps3.awi.de/Hustedt-Diatoms/FragCl/Set1/L041_W9.0-S7.7-PS1768-8.000582.png	41.2	8.9	7.7	NA	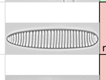	ritscheri	ritscheri	ritscheri	ritscheri	ritscheri	ritscheri	ritscheri	ritscheri	ritscheri	ritscheri	ritscheri	ritscheri	12	12	0	0	0	0	12	1	1	0	ritscheri												
http://apps3.awi.de/Hustedt-Diatoms/FragCl/Set1/L041_W9.0-S7.3-PS1768-8.000584.png	41.4	8.8	7.3	NA	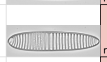	ritscheri	ritscheri	ritscheri	ritscheri	ritscheri	ritscheri	ritscheri	ritscheri	ritscheri	ritscheri	ritscheri	ritscheri	12	12	0	0	0	0	12	1	1	0	ritscheri												
http://apps3.awi.de/Hustedt-Diatoms/FragCl/Set1/L042_W7.0-S7.1-FragCl.NBP-1402.999-996cm.000003.png	41.5	7.1	7.1	NA	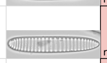	ritscheri	ritscheri	ritscheri	ritscheri	ritscheri	ritscheri	ritscheri	ritscheri	ritscheri	ritscheri	ritscheri	ritscheri	12	12	0	0	0	0	12	1	1	0	ritscheri												
http://apps3.awi.de/Hustedt-Diatoms/FragCl/Set1/L042_W10.0-S8.3-PS1768-8.000622.png	41.6	9.7	8.3	NA	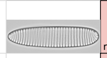	ritscheri	ritscheri	ritscheri	ritscheri	ritscheri	ritscheri	ritscheri	ritscheri	ritscheri	ritscheri	ritscheri	ritscheri	12	12	0	0	0	0	12	1	1	0	ritscheri												
http://apps3.awi.de/Hustedt-Diatoms/FragCl/Set1/L042_W7.0-S7.4-PS1768-8.000780.png	41.9	7.4	7.4	NA	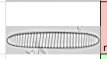	ritscheri	ritscheri	obliquecost	obliquecost	ritscheri	obliquecost	obliquecos	angulata	obliquecostata	obliquecostata	obliquecostata	ritscheri	12	4	7	0	0	1	7	0.583	2	0	obliquecostata												
http://apps3.awi.de/Hustedt-Diatoms/FragCl/Set1/L042_W6.0-S8.5-FragCl.ANT33-76.000044.png	41.9	6.1	8.5	NA	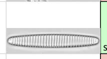	sublinearis	sublinearis	sublinearis	sublinearis	sublinearis	sublinearis	obliquecos	sublinearis	sublinearis	obliquecostata	sublinearis	sublinearis	12	0	2	10	0	0	10	0.833	3	0	sublinearis												
http://apps3.awi.de/Hustedt-Diatoms/FragCl/Set1/L042_W9.0-SNA-PS1768-8.000607.png	42.1	9.4	NA	8.8	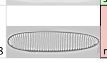	ritscheri	ritscheri	ritscheri	ritscheri	ritscheri	ritscheri	ritscheri	ritscheri	ritscheri	ritscheri	ritscheri	ritscheri	12	12	0	0	0	0	12	1	1	0	ritscheri												

http://apps3.awi.de/Hustedt-Diatoms/FragCl/Set1/L042_W6.0-S5.6-PS1768-8.000856.png	42.4	6.3	5.6	NA	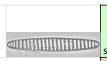	sublinearis	ambiguous	obliquecost	obliquecost	obliquecost	kerguelensi	obliquecos	sublinearis	obliquecostata	obliquecostata	sublinearis	obliquecostata		12	0	7	3	1	1	7	0.583	2	0	obliquecostata		obliquecostata - ritscheri				out of focus, maybe abberant ritscheri with coasre poroids						
http://apps3.awi.de/Hustedt-Diatoms/FragCl/Set1/L043_W10.0-S8.1-Hasle22-48.000005.png	42.6	9.8	8.1	NA	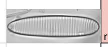	ritscheri	ritscheri	ritscheri	ritscheri	ritscheri	ritscheri	ritscheri	ritscheri	ritscheri	ritscheri	ritscheri	ritscheri		12	12	0	0	0	0	12	1	1	0	ritscheri												
http://apps3.awi.de/Hustedt-Diatoms/FragCl/Set1/L043_W7.0-SNA-FragCl.ANT33-51.000381.png	42.7	7.1	NA	8.4	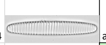	ambiguous	sublinearis	sublinearis	sublinearis	sublinearis	sublinearis		cylindrus	sublinearis	obliquecostata	sublinearis	sublinearis		11	0	1	8	1	1	8	0.727	3	0	sublinearis	?sublinearis/ritscherii											
http://apps3.awi.de/Hustedt-Diatoms/FragCl/Set1/L043_W7.0-S4.7-PS1768-8.000420.png	42.8	6.7	4.7	NA	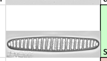	sublinearis	ambiguous	obliquecost	obliquecost	obliquecost	obliquecostata		kerguelensis	obliquecostata	obliquecostata	sublinearis	obliquecostata		11	0	7	2	1	1	7	0.636	2	0	obliquecostata		obliquecostata - ritscheri			difficult - ritscheri	but too small- maybe abberant ritscheri						
http://apps3.awi.de/Hustedt-Diatoms/FragCl/Set1/L043_W9.0-SNA-FragCl.NBP-1402.960-961cm.000058.png	43.0	9.3	NA	8.0	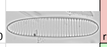	ritscheri	ritscheri	ritscheri	ritscheri	ritscheri	ritscheri		ritscheri	ritscheri	ritscheri	ritscheri	ritscheri		11	11	0	0	0	0	11	1	1	0	ritscheri												
http://apps3.awi.de/Hustedt-Diatoms/FragCl/Set1/L043_W6.0-S8.9-FragCl.ANT33-100.000054.png	43.0	6.4	8.9	9.1	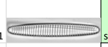	sublinearis	sublinearis	sublinearis	sublinearis	sublinearis	sublinearis		sublinearis	sublinearis	obliquecostata	sublinearis	sublinearis		11	0	1	10	0	0	10	0.909	3	0	sublinearis												
http://apps3.awi.de/Hustedt-Diatoms/FragCl/Set1/L043_W9.0-S5.5-PS1768-8.000561.png	43.0	8.8	5.5	NA	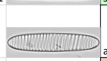	ambiguous	ritscheri	obliquecost	obliquecost	ritscheri	obliquecostata		ritscheri	obliquecostata	obliquecostata	obliquecostata	ritscheri		11	4	6	0	1	0	6	0.545	2	0	obliquecostata	?ritscherii/kerguelensis					but too small- maybe abberant ritscheri						
http://apps3.awi.de/Hustedt-Diatoms/FragCl/Set1/L044_W9.0-S6.7-PS1768-8.000571.png	43.7	9.4	6.7	NA	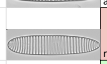	ritscheri	ritscheri	ritscheri	ritscheri	ritscheri	ritscheri		ritscheri	ritscheri	ritscheri	ritscheri	ritscheri		11	11	0	0	0	0	11	1	1	0	ritscheri				Curved valve (I have never seen it before)								
http://apps3.awi.de/Hustedt-Diatoms/FragCl/Set1/L044_W7.0-S8.8-FragCl.ANT33-100.000090.png	43.8	6.9	8.8	8.6	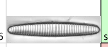	sublinearis	sublinearis	sublinearis	sublinearis	sublinearis	sublinearis		peragallii	sublinearis	obliquecostata	sublinearis	sublinearis		11	0	1	9	0	1	9	0.818	3	0	sublinearis												
http://apps3.awi.de/Hustedt-Diatoms/FragCl/Set1/L044_W8.0-S8.4-Hasle22-47.000018.png	43.8	8.4	8.4	NA	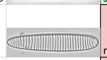	ritscheri	ritscheri	ritscheri	ritscheri	ritscheri	ritscheri		ritscheri	ritscheri	ritscheri	ritscheri	ritscheri		11	11	0	0	0	0	11	1	1	0	ritscheri												
http://apps3.awi.de/Hustedt-Diatoms/FragCl/Set1/L044_W10.0-SNA-FragCl.ANT33-51.000028.png	43.9	10.4	NA	8.7	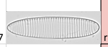	ritscheri	ritscheri	ritscheri	ritscheri	ritscheri	ritscheri		ritscheri	ritscheri	ritscheri	ritscheri	ritscheri		11	11	0	0	0	0	11	1	1	0	ritscheri												
http://apps3.awi.de/Hustedt-Diatoms/FragCl/Set1/L044_W8.0-S7.6-FragCl.NBP-1402.945-946cm.000063.png	44.2	7.8	7.6	NA	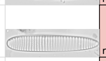	ritscheri	ritscheri	ritscheri	ritscheri	ritscheri	ritscheri		ritscheri	ritscheri	ritscheri	ritscheri	ritscheri		11	11	0	0	0	0	11	1	1	0	ritscheri												
http://apps3.awi.de/Hustedt-Diatoms/FragCl/Set1/L044_W9.0-S7.8-PS1768-8.000628.png	44.3	9.0	7.8	NA	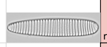	ritscheri	ritscheri	ritscheri	ritscheri	ritscheri	ritscheri		ritscheri	ritscheri	ritscheri	ritscheri	ritscheri		11	11	0	0	0	0	11	1	1	0	ritscheri												
http://apps3.awi.de/Hustedt-Diatoms/FragCl/Set1/L044_W8.0-S6.1-PS1768-8.000862.png	44.3	7.9	6.1	NA	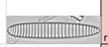	ritscheri	ritscheri	ritscheri	ritscheri	ritscheri	ritscheri		ritscheri	ritscheri	ritscheri	ritscheri	ritscheri		11	11	0	0	0	0	11	1	1	0	ritscheri												
http://apps3.awi.de/Hustedt-Diatoms/FragCl/Set1/L044_W9.0-S7.4-Hasle22-47.000025.png	44.5	8.5	7.4	NA	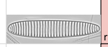	ritscheri	ritscheri	ritscheri	ritscheri	ritscheri	ritscheri		ritscheri	ritscheri	ritscheri	ritscheri	ritscheri		11	11	0	0	0	0	11	1	1	0	ritscheri												
http://apps3.awi.de/Hustedt-Diatoms/FragCl/Set1/L045_W10.0-S8.9-FragCl.ANT33-51.000013.png	44.5	9.6	8.9	9.3	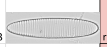	ritscheri	ritscheri	ritscheri	ritscheri	ritscheri	ritscheri		ritscheri	ritscheri	ritscheri	ritscheri	ritscheri		11	11	0	0	0	0	11	1	1	0	ritscheri												
http://apps3.awi.de/Hustedt-Diatoms/FragCl/Set1/L045_W9.0-S6.1-Wa-75b_Wa-77b.000166.png	44.6	8.6	6.1	NA	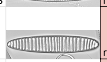	ritscheri	ritscheri	ritscheri	ritscheri	ritscheri	ritscheri	ritscheri	ritscheri	ritscheri	ritscheri	obliquecostata	ritscheri		12	11	1	0	0	0	11	0.916	1	0	ritscheri												
http://apps3.awi.de/Hustedt-Diatoms/FragCl/Set1/L045_W11.0-S9.1-PS1768-8.000581.png	44.6	10.8	9.1	NA	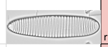	ritscheri	ritscheri	ritscheri	ritscheri	ritscheri	ritscheri		ritscheri	ritscheri	ritscheri	ritscheri	ritscheri		11	11	0	0	0	0	11	1	1	0	ritscheri												
http://apps3.awi.de/Hustedt-Diatoms/FragCl/Set1/L045_W8.0-SNA-FragCl.NBP-1402.945-946cm.000079.png	44.6	8.0	NA	6.4	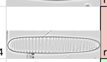	ritscheri	ritscheri	ritscheri	ritscheri	ritscheri	ritscheri	ritscheri	ritscheri	ritscheri	ritscheri	ritscheri	ritscheri		12	12	0	0	0	0	12	1	1	0	ritscheri												
http://apps3.awi.de/Hustedt-Diatoms/FragCl/Set1/L045_W6.0-S7.9-PS1768-8.000766.png	44.7	5.8	7.9	NA	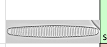	sublinearis	sublinearis	sublinearis	sublinearis	sublinearis	sublinearis		sublinearis	sublinearis	obliquecostata	sublinearis	sublinearis		11	0	1	10	0	0	10	0.909	3	0	sublinearis												
http://apps3.awi.de/Hustedt-Diatoms/FragCl/Set1/L045_W9.0-S10.1-FragCl.ANT33-51.000127.png	44.8	9.4	10.1	9.6	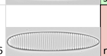	ritscheri	ritscheri	ritscheri	ritscheri	ritscheri	ritscheri	ritscheri	ritscheri	ritscheri	ritscheri	ritscheri	ritscheri		12	12	0	0	0	0	12	1	1	0	ritscheri				(= picture 156)								
http://apps3.awi.de/Hustedt-Diatoms/FragCl/Set1/L045_W8.0-S9.5-PS1768-8.000507.png	44.9	8.4	9.5	NA	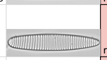	ritscheri	ritscheri	ritscheri	ritscheri	ritscheri	ritscheri	ritscheri	ritscheri	ritscheri	ritscheri	ambiguous	ritscheri		12	11	0	0	1	0	11	0.916	1	0	ritscheri											obliquecostata?: oblique costae, bu	
http://apps3.awi.de/Hustedt-Diatoms/FragCl/Set1/L045_W8.0-S7.9-FragCl.NBP-1402.945-946cm.000082.png	45.1	8.1	7.9	NA	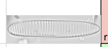	ritscheri	ritscheri	ritscheri	ritscheri	ritscheri	ritscheri		ritscheri	ritscheri	ritscheri	ritscheri	ritscheri		11	11	0	0	0	0	11	1	1	0	ritscheri												
http://apps3.awi.de/Hustedt-Diatoms/FragCl/Set1/L045_W6.0-S7.6-PS1768-8.000515.png	45.3	5.6	7.6	NA	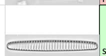	sublinearis	sublinearis	sublinearis	sublinearis	sublinearis	sublinearis		sublinearis	sublinearis	obliquecostata	sublinearis	sublinearis		11	0	1	10	0	0	10	0.909	3	0	sublinearis												
http://apps3.awi.de/Hustedt-Diatoms/FragCl/Set1/L046_W8.0-SNA-Hasle22-47.000008.png	45.5	8.2	NA	8.8	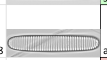	ambiguous	ambiguous	curta	curta	ritscheri	linearis	obliquecos	peragallii	ambiguous	sublinearis	ambiguous			11	1	1	1	4	4	4	0.363	4	1	ambiguous	?linearis/ritscheri	F. linearis?			inflated valve out	isopolar, linear va	Could also be another species (pera		F.curta possibly		ritscheri?: not strictly heteropolar	
http://apps3.awi.de/Hustedt-Diatoms/FragCl/Set1/L046_W9.0-S8.9-PS1768-8.000791.png	45.5	8.8	8.9	NA	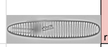	ritscheri	ritscheri	ritscheri	ritscheri	ritscheri	ritscheri	ritscheri	ritscheri	ritscheri	ritscheri	ritscheri	ritscheri		12	12	0	0	0	0	12	1	1	0	ritscheri												
http://apps3.awi.de/Hustedt-Diatoms/FragCl/Set1/L046_W10.0-S5.9-PS1768-8.000578.png	45.6	9.7	5.9	NA	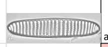	ambiguous	ritscheri	obliquecost	obliquecost	ritscheri	ritscheri	obliquecos	angulata	ambiguous	sublinearis	ritscheri	ritscheri		12	5	3	1	2	1	5	0.416	1	0	ritscheri	?ritscherii/kerguelensis		again a contorted specimen			abberrant form- kerguelensis like poroids			F.kerguelensis possibly			
http://apps3.awi.de/Hustedt-Diatoms/FragCl/Set1/L046_W8.0-SNA-FragCl.NBP-1402.945-946cm.000050.png	45.9	7.7	NA	8.1	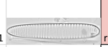	ritscheri	ritscheri	ritscheri	ritscheri	ritscheri	ritscheri	ritscheri	ritscheri	ritscheri	ritscheri	ritscheri	ritscheri		12	12	0	0	0	0	12	1	1	0	ritscheri												
http://apps3.awi.de/Hustedt-Diatoms/FragCl/Set1/L046_W7.0-S6.8-PS1768-8.000535.png	46.2	6.9	6.8	NA	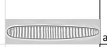	ambiguous	ritscheri	ritscheri	ritscheri	ritscheri	ritscheri	ritscheri	ritscheri	ritscheri	ritscheri	ritscheri	ritscheri		12	11	0	0	1	0	11	0.916	1	0	ritscheri	?ritscherii/kerguelensis						not 100% sure could also be obliquecostata					
http://apps3.awi.de/Hustedt-Diatoms/FragCl/Set1/L046_W8.0-S5.9-PS1768-8.000073.png	46.3	8.2	5.9	NA	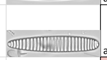	ambiguous	ritscheri	ritscheri	ritscheri	ritscheri	ritscheri	ritscheri	ritscheri	ritscheri	ritscheri	ritscheri	ritscheri		12	11	0	0	1	0	11	0.916	1	0	ritscheri	?kerguelensis/ritscherii											
http://apps3.awi.de/Hustedt-Diatoms/FragCl/Set1/L046_W8.0-S7.3-Hasle22-47.000019.png	46.4	8.3	7.3	NA	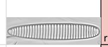	ritscheri	ritscheri	ritscheri	ritscheri	ritscheri	ritscheri	ritscheri	ritscheri	ritscheri	ritscheri	ritscheri	ritscheri		12	12	0	0	0	0	12	1	1	0	ritscheri												
http://apps3.awi.de/Hustedt-Diatoms/FragCl/Set1/L046_W9.0-S6.7-PS1768-8.000779.png	46.5	8.6	6.7	NA	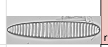	ritscheri	ritscheri	ritscheri	ritscheri	ritscheri	ritscheri	ritscheri	ritscheri	ritscheri	ritscheri	ritscheri	ritscheri		12	12	0	0	0	0	12	1	1	0	ritscheri												
http://apps3.awi.de/Hustedt-Diatoms/FragCl/Set1/L047_W9.0-S6.6-Wa-75b_Wa-77b.000165.png	46.9	8.7	6.6	NA	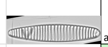	ambiguous	ambiguous	ritscheri	ritscheri	ritscheri	ritscheri		angulata	ambiguous	ritscheri	sublinearis	ritscheri		11	6	0	1	3	1	6	0.545	1	0	ritscheri	?ritscherii/kergue	most similiar to a finer F. kerguelen		More lanceolated than typical shap		could be kerguelensis			unsure...			
http://apps3.awi.de/Hustedt-Diatoms/FragCl/Set1/L047_W6.0-S8.3-PS1768-8.000462.png	47.0	6.1	8.3	NA	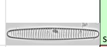	sublinearis	sublinearis	sublinearis	sublinearis	sublinearis	sublinearis	obliquecos	sublinearis	sublinearis	obliquecostata	sublinearis	sublinearis		12	0	2	10	0	0	10	0.833	3	0	sublinearis												
http://apps3.awi.de/Hustedt-Diatoms/FragCl/Set1/L047_W8.0-S9.0-Hasle22-47.000022.png	47.0	8.2	9.0	NA	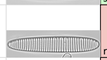	ritscheri	ritscheri	ritscheri	ritscheri	ritscheri	ritscheri		ritscheri	ritscheri	ritscheri	ritscheri	ritscheri		11	11	0	0	0	0	11	1	1	0	ritscheri												
http://apps3.awi.de/Hustedt-Diatoms/FragCl/Set1/L047_W8.0-S6.0-FragCl.NBP-1402.945-946cm.000003.png	47.1	8.2	6.0	NA	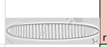	ritscheri	ritscheri	ritscheri	ritscheri	ritscheri	ritscheri	ritscheri	ritscheri	ritscheri	ritscheri	ritscheri	ritscheri		12	12	0	0	0	0	12	1	1	0	ritscheri												
http://apps3.awi.de/Hustedt-Diatoms/FragCl/Set1/L047_W6.0-S9.1-FragCl.ANT33-76.000053.png	47.3	6.2	9.1	NA	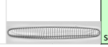	sublinearis	sublinearis	sublinearis	sublinearis	sublinearis	sublinearis	obliquecos	sublinearis	sublinearis	obliquecostata	sublinearis	sublinearis		12	0	2	10	0	0	10	0.833	3	0	sublinearis												

http://apps3.awi.de/Hustedt-Diatoms/FragCl/Set1/L047_W7.0-S5.2-FragCl.NBP-1402.945-946cm.000078.png	47.4	7.5	5.2	NA	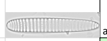	ambiguous	ritscheri	ritscheri	ritscheri	ritscheri	ritscheri	ritscheri	ritscheri	ritscheri	ritscheri	ritscheri	ritscheri	12	11	0	0	1	0	11	0.916	1	0	ritscheri	?ritscheri/obliquecostata											
http://apps3.awi.de/Hustedt-Diatoms/FragCl/Set1/L047_W7.0-S8.4-PS1768-8.000431.png	47.5	6.8	8.4	NA	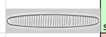	sublinearis	sublinearis	sublinearis	sublinearis	sublinearis	sublinearis	obliquecos	sublinearis	ambiguous	sublinearis	sublinearis	sublinearis	12	0	1	10	1	0	10	0.833	3	0	sublinearis									sublinearis if marginal poroids were more distinct on both sides			
http://apps3.awi.de/Hustedt-Diatoms/FragCl/Set1/L048_W11.0-S7.1-PS1768-8.000557.png	47.6	10.5	7.1	NA	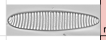	ritscheri	ritscheri	ritscheri	ritscheri	ritscheri	ritscheri		ritscheri		ritscheri	ritscheri	ritscheri	10	10	0	0	0	0	10	1	1	0	ritscheri												
http://apps3.awi.de/Hustedt-Diatoms/FragCl/Set1/L048_W8.0-S7.5-FragCl.NBP-1402.945-946cm.000002.png	47.7	7.9	7.5	NA	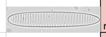	ritscheri	ritscheri	ritscheri	ritscheri	ritscheri	ritscheri		ritscheri	ritscheri	ritscheri	ritscheri	ritscheri	11	11	0	0	0	0	11	1	1	0	ritscheri												
http://apps3.awi.de/Hustedt-Diatoms/FragCl/Set1/L048_W10.0-S7.7-PS1768-8.000552.png	47.8	9.8	7.7	NA	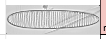	ritscheri	ritscheri	ritscheri	ritscheri	ritscheri	ritscheri		ritscheri		ritscheri	ritscheri	ritscheri	10	10	0	0	0	0	10	1	1	0	ritscheri												
http://apps3.awi.de/Hustedt-Diatoms/FragCl/Set1/L048_W8.0-S5.6-FragCl.NBP-1402.945-946cm.000080.png	47.9	7.5	5.6	NA	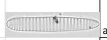	ambiguous	ritscheri	ritscheri	ritscheri	ritscheri	ritscheri		ritscheri	ritscheri	ritscheri	ritscheri	ritscheri	11	10	0	0	1	0	10	0.909	1	0	ritscheri	?ritscheri/obliquecostata											
http://apps3.awi.de/Hustedt-Diatoms/FragCl/Set1/L048_W8.0-S5.1-PS1768-8.000424.png	47.9	7.6	5.1	NA	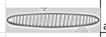	ambiguous	obliquecost	obliquecost	obliquecost	obliquecost	obliquecostata		peragallii	obliquecostata	obliquecostata	obliquecostata	obliquecostata	11	0	9	0	1	1	9	0.818	2	0	obliquecostata	?ritscherii/kergue	apical axis a little short				abberant form- weakly heteropolar, oblique costae, could be very very small ritscheri with very coarse costae and areolae.						
http://apps3.awi.de/Hustedt-Diatoms/FragCl/Set1/L048_W9.0-S5.8-PS1768-8.000760.png	48.0	8.8	5.8	NA	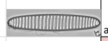	ambiguous	ritscheri	obliquecost	obliquecost	ritscheri	ritscheri		peragallii	obliquecostata	obliquecostata	obliquecostata	obliquecostata	11	3	6	0	1	1	6	0.545	2	0	obliquecostata	?ritscherii/kerguelensis					abberant form- weakly heteropolar, slightly oblique costae.						
http://apps3.awi.de/Hustedt-Diatoms/FragCl/Set1/L048_W9.0-SNA-FragCl.NBP-1402.960-961cm.000110.png	48.0	8.8	NA	8.2	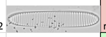	ritscheri	ritscheri	ritscheri	ritscheri	ritscheri	ritscheri		ritscheri	ritscheri	ritscheri	ritscheri	ritscheri	11	11	0	0	0	0	11	1	1	0	ritscheri									poss F. pergallii but unsure			
http://apps3.awi.de/Hustedt-Diatoms/FragCl/Set1/L048_W5.0-SNA-FragCl.NBP-1402.945-946cm.000038.png	48.1	5.3	NA	7.9	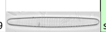	sublinearis	sublinearis	sublinearis	sublinearis	sublinearis	sublinearis		sublinearis	sublinearis	sublinearis	sublinearis	sublinearis	11	0	0	11	0	0	11	1	3	0	sublinearis												
http://apps3.awi.de/Hustedt-Diatoms/FragCl/Set1/L048_W9.0-S7.4-FragCl.NBP-1402.960-961cm.000057.png	48.1	9.3	7.4	NA	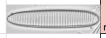	ritscheri	ritscheri	ritscheri	ritscheri	ritscheri	ritscheri		ritscheri	ritscheri	ritscheri	ritscheri	ritscheri	11	11	0	0	0	0	11	1	1	0	ritscheri												
http://apps3.awi.de/Hustedt-Diatoms/FragCl/Set1/L048_W9.0-S5.6-PS1768-8.000486.png	48.2	9.0	5.6	NA	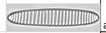	ambiguous	ritscheri	obliquecost	obliquecost	ritscheri	ritscheri			ambiguous	ritscheri	obliquecostata	obliquecostata	10	4	4	0	2	0	4	0.4	1	1	ambiguous	?ritscherii/kerguelensis					abberant form- kerguelensis like poroids			obliquecostata or kerguelensis?			
http://apps3.awi.de/Hustedt-Diatoms/FragCl/Set1/L048_W8.0-S6.8-FragCl.NBP-1402.960-961cm.000078.png	48.2	8.3	6.8	NA	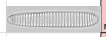	ritscheri	ritscheri	ritscheri	ritscheri	ritscheri	ritscheri		ritscheri	ambiguous	ritscheri	ritscheri	ritscheri	11	10	0	0	1	0	10	0.909	1	0	ritscheri									probably ritscheri but not sure			
http://apps3.awi.de/Hustedt-Diatoms/FragCl/Set1/L048_W8.0-S5.6-FragCl.NBP-1402.945-946cm.000005.png	48.2	8.1	5.6	NA	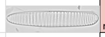	ritscheri	ritscheri	ritscheri	ritscheri	ritscheri	ritscheri		ritscheri	ritscheri	ritscheri	ritscheri	ritscheri	11	11	0	0	0	0	11	1	1	0	ritscheri												
http://apps3.awi.de/Hustedt-Diatoms/FragCl/Set1/L048_W8.0-S5.9-PS1768-8.000082.png	48.3	8.3	5.9	NA	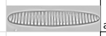	ambiguous	ritscheri	ritscheri	ritscheri	ritscheri	ritscheri		ritscheri	ritscheri	ritscheri	obliquecostata	ritscheri	11	9	1	0	1	0	9	0.818	1	0	ritscheri	?kerguelensis/ritscherii											
http://apps3.awi.de/Hustedt-Diatoms/FragCl/Set1/L048_W8.0-S5.9-PS1768-8.000580.png	48.3	7.7	5.9	NA	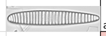	ambiguous	ritscheri	ritscheri	ritscheri	ritscheri	ritscheri		ritscheri	ritscheri	ritscheri	ritscheri	ritscheri	11	10	0	0	1	0	10	0.909	1	0	ritscheri	?ritscherii/kerguelensis											
http://apps3.awi.de/Hustedt-Diatoms/FragCl/Set1/L048_W7.0-SNA-FragCl.NBP-1402.945-946cm.000077.png	48.4	7.2	NA	5.6	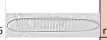	ritscheri	ritscheri	ritscheri	ritscheri	ritscheri	ritscheri		ritscheri	ritscheri	ritscheri	ritscheri	ritscheri	11	11	0	0	0	0	11	1	1	0	ritscheri		large end blunter than average										
http://apps3.awi.de/Hustedt-Diatoms/FragCl/Set1/L048_W8.0-S6.1-PS1768-8.000416.png	48.5	7.6	6.1	NA	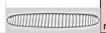	ritscheri	ritscheri	obliquecost	obliquecost	obliquecost	ritscheri			obliquecostata	obliquecostata	obliquecostata	obliquecostata	10	3	7	0	0	0	7	0.7	2	0	obliquecostata					although has ritsc	abberant form- weakly heteropolar, oblique costae, could be very very small obliqeucostata but doubt it. Same as code 151, 16						
http://apps3.awi.de/Hustedt-Diatoms/FragCl/Set1/L049_W5.0-S9.6-FragCl.ANT33-51.000365.png	48.6	5.1	9.6	NA	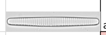	ambiguous	vanheurckii	vanheurckii	sublinearis	vanheurckii	sublinearis	obliquecos	vanheurckii	ambiguous	sublinearis	ambiguous	vanheurckii	12	0	1	3	3	5	5	0.416	5	0	nonTarget	?sublinearis/vanheurckii					looks suspciously like F. vanheurkii (pseudo-nodulus?			sublinearis or vanhuerckii		vanheurckii?: speciment seems rath	
http://apps3.awi.de/Hustedt-Diatoms/FragCl/Set1/L049_W10.0-S7.5-PS1768-8.000585.png	48.7	10.1	7.5	NA	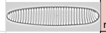	ritscheri	ritscheri	ritscheri	ritscheri	ritscheri	ritscheri		ritscheri	ritscheri	ritscheri	ritscheri	ritscheri	11	11	0	0	0	0	11	1	1	0	ritscheri												
http://apps3.awi.de/Hustedt-Diatoms/FragCl/Set1/L049_W8.0-S6.8-FragCl.NBP-1402.945-946cm.000007.png	48.9	7.8	6.8	NA	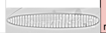	ritscheri	ritscheri	ritscheri	ritscheri	ritscheri	ritscheri	ritscheri	ritscheri	ritscheri	ritscheri	ritscheri	ritscheri	12	12	0	0	0	0	12	1	1	0	ritscheri												
http://apps3.awi.de/Hustedt-Diatoms/FragCl/Set1/L049_W8.0-S7.5-Hasle22-47.000017.png	48.9	8.2	7.5	NA	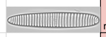	ritscheri	ritscheri	ritscheri	ritscheri	ritscheri	ritscheri	ritscheri	ritscheri	ritscheri	ritscheri	ritscheri	ritscheri	12	12	0	0	0	0	12	1	1	0	ritscheri												
http://apps3.awi.de/Hustedt-Diatoms/FragCl/Set1/L049_W10.0-S9.1-PS1768-8.000549.png	49.2	9.8	9.1	NA	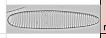	ritscheri	ritscheri	ritscheri	ritscheri	ritscheri	ritscheri	ritscheri	ritscheri	ritscheri	ritscheri	ritscheri	ritscheri	12	12	0	0	0	0	12	1	1	0	ritscheri												
http://apps3.awi.de/Hustedt-Diatoms/FragCl/Set1/L049_W9.0-S7.0-FragCl.ANT33-51.000067.png	49.2	8.9	7.0	7.0	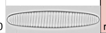	ritscheri	ritscheri	obliquecost	obliquecost	ritscheri	obliquecost	obliquecostata		ambiguous	obliquecostata	obliquecostata	obliquecostata	11	3	7	0	1	0	7	0.636	2	0	obliquecostata			some flavor of sublinearis, not sure		but not typical - slanted striae but vaguely heteropolar				ritscheri or sublinearis			
http://apps3.awi.de/Hustedt-Diatoms/FragCl/Set1/L049_W11.0-S8.6-FragCl.ANT33-51.000359.png	49.2	10.8	8.6	NA	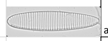	ambiguous	ritscheri	ritscheri	ritscheri	ritscheri	ritscheri	ritscheri	ritscheri	ritscheri	ritscheri	ritscheri	ritscheri	12	11	0	0	1	0	11	0.916	1	0	ritscheri	?ritscherii/obliquecostata											
http://apps3.awi.de/Hustedt-Diatoms/FragCl/Set1/L049_W9.0-S6.4-PS1768-8.000528.png	49.2	8.7	6.4	NA	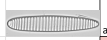	ambiguous	ritscheri	ritscheri	ritscheri	ritscheri	ritscheri	ritscheri	ritscheri	ritscheri	ritscheri	ritscheri	ritscheri	12	11	0	0	1	0	11	0.916	1	0	ritscheri	?ritscherii/kerguelensis											
http://apps3.awi.de/Hustedt-Diatoms/FragCl/Set1/L049_W9.0-S6.7-PS1768-8.000430.png	49.3	9.3	6.7	NA	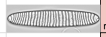	ritscheri	ritscheri	obliquecost	obliquecost	ritscheri	obliquecost	obliquecostata		ambiguous	obliquecostata	obliquecostata	obliquecostata	11	3	7	0	1	0	7	0.636	2	0	obliquecostata					but not typical - s	could be ritscheri, but inflation is middle			probably mutant F. obliquecostata			
http://apps3.awi.de/Hustedt-Diatoms/FragCl/Set1/L049_W9.0-S7.6-PS1768-8.000512.png	49.3	9.4	7.6	NA	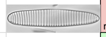	ritscheri	ritscheri	ritscheri	ritscheri	ritscheri	ritscheri	ritscheri	ritscheri	ambiguous	ritscheri	obliquecostata	ritscheri	12	10	1	0	1	0	10	0.833	1	0	ritscheri						could be confused with obliquecostata due to slightly			ritscheri or sublinearis			
http://apps3.awi.de/Hustedt-Diatoms/FragCl/Set1/L049_W7.0-S6.8-PS1768-8.000972.png	49.3	6.7	6.8	NA	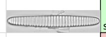	sublinearis	sublinearis	sublinearis	obliquecost	sublinearis	obliquecost	obliquecostata		ambiguous	sublinearis	obliquecostata	obliquecostata	11	0	5	5	1	0	5	0.454	2	1	ambiguous					feels too robust, l	abberant form- w	not 100% sure could be sublinearis		sublinearis or obliquecostata depending on marginal poroids			
http://apps3.awi.de/Hustedt-Diatoms/FragCl/Set1/L049_W8.0-SNA-FragCl.NBP-1402.945-946cm.000075.png	49.3	8.3	NA	7.3	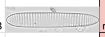	ritscheri	ritscheri	ritscheri	ritscheri	ritscheri	ritscheri	ritscheri	ritscheri	ritscheri	ritscheri	ritscheri	ritscheri	12	12	0	0	0	0	12	1	1	0	ritscheri									poss F. pergallii but unsure			
http://apps3.awi.de/Hustedt-Diatoms/FragCl/Set1/L049_W9.0-S7.8-PS1768-8.000852.png	49.4	9.3	7.8	NA	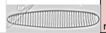	ritscheri	ritscheri	ritscheri	ritscheri	ritscheri	ritscheri	ritscheri	ritscheri	ritscheri	ritscheri	ritscheri	ritscheri	12	12	0	0	0	0	12	1	1	0	ritscheri												
http://apps3.awi.de/Hustedt-Diatoms/FragCl/Set1/L050_W6.0-S8.5-FragCl.ANT33-51.000368.png	49.6	6.0	8.5	NA	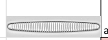	ambiguous	sublinearis	sublinearis	sublinearis	sublinearis	sublinearis	obliquecostata		sublinearis	sublinearis	sublinearis	sublinearis	11	0	1	9	1	0	9	0.818	3	0	sublinearis	?sublinearis/vanheurckii											
http://apps3.awi.de/Hustedt-Diatoms/FragCl/Set1/L050_W9.0-S7.2-FragCl.NBP-1402.945-946cm.000043.png	49.8	8.7	7.2	NA	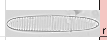	ritscheri	ritscheri	ritscheri	ritscheri	ritscheri	ritscheri		ritscheri	ambiguous	ritscheri	obliquecostata	ritscheri	11	9	1	0	1	0	9	0.818	1	0	ritscheri									ritscheri or obliquecostata			
http://apps3.awi.de/Hustedt-Diatoms/FragCl/Set1/L050_W9.0-S8.6-FragCl.NBP-1402.999-996cm.000006.png	49.8	8.6	8.6	NA	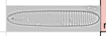	ritscheri	ritscheri	ritscheri	ritscheri	ritscheri	ritscheri		ritscheri	ritscheri	ritscheri	obliquecostata	ritscheri	11	10	1	0	0	0	10	0.909	1	0	ritscheri												
http://apps3.awi.de/Hustedt-Diatoms/FragCl/Set1/L050_W7.0-S7.0-FragCl.NBP-1402.945-946cm.000071.png	49.8	7.4	7.0	NA	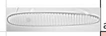	ambiguous	ritscheri	ritscheri	ritscheri	ritscheri	ritscheri	ritscheri	ritscheri	ritscheri	ritscheri	ambiguous	ritscheri	12	10	0	0	2	0	10	0.833	1	0	ritscheri	?ritscheri/obliquecostata										ritscheri?: specimen not in focus	
http://apps3.awi.de/Hustedt-Diatoms/FragCl/Set1/L050_W8.0-S5.7-PS1768-8.000533.png	50.0	8.4	5.7	NA	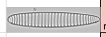	ritscheri	ritscheri	ritscheri	ritscheri	ritscheri	ritscheri	ritscheri	ritscheri	ritscheri	ritscheri	ritscheri	ritscheri	12	12	0	0	0	0	12	1	1	0	ritscheri												
http://apps3.awi.de/Hustedt-Diatoms/FragCl/Set1/L050_W8.0-S8.5-FragCl.NBP-1402.999-996cm.000005.png	50.0	8.4	8.5	NA	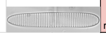	ritscheri	ritscheri	ritscheri	ritscheri	ritscheri	ritscheri		ritscheri	ritscheri	ritscheri	ritscheri	ritscheri	11	11	0	0	0	0	11	1	1	0	ritscheri												

http://apps3.awi.de/Hustedt-Diatoms/FragCl/Set1/L050_W7.0-SNA-FragCl.ANT33-51.000120.png	50.2	7.0	NA	8.5	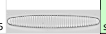	sublinearis	sublinearis	sublinearis	sublinearis	sublinearis	sublinearis	obliquecostata		sublinearis	sublinearis	sublinearis	sublinearis	11	0	1	10	0	0	10	0.909	3	0	sublinearis												
http://apps3.awi.de/Hustedt-Diatoms/FragCl/Set1/L050_W8.0-SNA-FragCl.NBP-1402.945-946cm.000021.png	50.3	8.1	NA	6.3	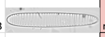	ritscheri	ritscheri	ritscheri	ritscheri	ritscheri	ritscheri		ritscheri	ritscheri	ritscheri	ritscheri	ritscheri	11	11	0	0	0	0	11	1	1	0	ritscheri												
http://apps3.awi.de/Hustedt-Diatoms/FragCl/Set1/L050_W8.0-S6.7-FragCl.NBP-1402.945-946cm.000019.png	50.3	7.6	6.7	NA	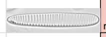	ritscheri	ritscheri	ritscheri	ritscheri	ritscheri	ritscheri		ritscheri	ritscheri	ritscheri	obliquecostata	ritscheri	11	10	1	0	0	0	10	0.909	1	0	ritscheri												
http://apps3.awi.de/Hustedt-Diatoms/FragCl/Set1/L050_W8.0-S5.9-FragCl.NBP-1402.945-946cm.000081.png	50.3	8.3	5.9	NA	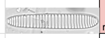	ritscheri	ritscheri	ritscheri	ritscheri	ritscheri	ritscheri	ritscheri	ritscheri	ritscheri	ritscheri	ritscheri	ritscheri	12	12	0	0	0	0	12	1	1	0	ritscheri												
http://apps3.awi.de/Hustedt-Diatoms/FragCl/Set1/L050_W8.0-S5.9-PS1768-8.000499.png	50.4	8.1	5.9	NA	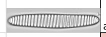	ambiguous	ambiguous	obliquecost	obliquecost	ritscheri	obliquecost	obliquecostata		obliquecostata	obliquecostata	obliquecostata	obliquecostata	11	1	8	0	2	0	8	0.727	2	0	obliquecostata	?ritscherii/obliqu	deformed			valve outline not	abberant form - could be ritscheri abbernat with obilque costae						
http://apps3.awi.de/Hustedt-Diatoms/FragCl/Set1/L050_W8.0-S6.0-FragCl.NBP-1402.945-946cm.000023.png	50.5	8.2	6.0	NA	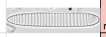	ritscheri	ritscheri	ritscheri	ritscheri	ritscheri	ritscheri	ritscheri	ritscheri	ritscheri	ritscheri	ritscheri	ritscheri	12	12	0	0	0	0	12	1	1	0	ritscheri												
http://apps3.awi.de/Hustedt-Diatoms/FragCl/Set1/L051_W9.0-S6.6-PS1768-8.000007.png	50.6	8.8	6.6	NA	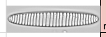	ritscheri	ritscheri	obliquecost	obliquecost	obliquecost	ritscheri	obliquecostata		obliquecostata	obliquecostata	obliquecostata	obliquecostata	11	3	8	0	0	0	8	0.727	2	0	obliquecostata					poles the same, s	abberant form or non target species due to isopolar ends- almost F. oceanica like.						
http://apps3.awi.de/Hustedt-Diatoms/FragCl/Set1/L051_W8.0-S7.6-FragCl.NBP-1402.945-946cm.000014.png	50.6	8.0	7.6	NA	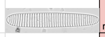	ritscheri	ritscheri	ritscheri	ritscheri	ritscheri	ritscheri	ritscheri	ritscheri	ritscheri	ritscheri	ritscheri	ritscheri	12	12	0	0	0	0	12	1	1	0	ritscheri												
http://apps3.awi.de/Hustedt-Diatoms/FragCl/Set1/L051_W7.0-S6.1-FragCl.NBP-1402.945-946cm.000058.png	50.7	7.1	6.1	NA	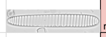	ritscheri	ritscheri	ritscheri	ritscheri	ritscheri	ritscheri		ritscheri	ritscheri	ritscheri	obliquecostata	ritscheri	11	10	1	0	0	0	10	0.909	1	0	ritscheri												
http://apps3.awi.de/Hustedt-Diatoms/FragCl/Set1/L051_W9.0-S6.9-FragCl.NBP-1402.960-961cm.000075.png	50.7	8.9	6.9	NA	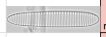	ritscheri	ritscheri	ritscheri	ritscheri	ritscheri	ritscheri	ritscheri	ritscheri	ritscheri	ritscheri	ritscheri	ritscheri	12	12	0	0	0	0	12	1	1	0	ritscheri												
http://apps3.awi.de/Hustedt-Diatoms/FragCl/Set1/L051_W7.0-SNA-FragCl.NBP-1402.945-946cm.000006.png	50.8	7.4	NA	8.5	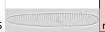	ritscheri	ritscheri	ritscheri	ritscheri	ritscheri	ritscheri		ritscheri	ritscheri	ritscheri	ritscheri	ritscheri	11	11	0	0	0	0	11	1	1	0	ritscheri												
http://apps3.awi.de/Hustedt-Diatoms/FragCl/Set1/L051_W7.0-S8.3-FragCl.ANT33-51.000518.png	50.9	7.0	8.3	NA		ambiguous	sublinearis	sublinearis	sublinearis	sublinearis	sublinearis			sublinearis	sublinearis	sublinearis	sublinearis	10	0	0	9	1	0	9	0.9	3	0	sublinearis	?sublinearis/ritscherii					internal valve view?						
http://apps3.awi.de/Hustedt-Diatoms/FragCl/Set1/L051_W6.0-SNA-FragCl.ANT33-100.000081.png	51.0	5.8	NA	7.8	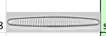	sublinearis	sublinearis	sublinearis	sublinearis	sublinearis	sublinearis		sublinearis	sublinearis	obliquecostata	sublinearis	sublinearis	11	0	1	10	0	0	10	0.909	3	0	sublinearis												
http://apps3.awi.de/Hustedt-Diatoms/FragCl/Set1/L051_W5.0-S9.0-PS1768-8.000784.png	51.1	5.2	9.0	NA	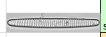	sublinearis	sublinearis	sublinearis	sublinearis	sublinearis	sublinearis		sublinearis	ambiguous	obliquecostata	sublinearis	sublinearis	11	0	1	9	1	0	9	0.818	3	0	sublinearis									sublinearis or obliquecostata depending on marginal poroids			
http://apps3.awi.de/Hustedt-Diatoms/FragCl/Set1/L051_W7.0-SNA-FragCl.NBP-1402.945-946cm.000056.png	51.1	7.3	NA	6.8	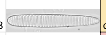	obliquecost	ritscheri	ritscheri	ritscheri	ritscheri	ritscheri		ritscheri	ritscheri	ritscheri	ritscheri	ritscheri	11	10	1	0	0	0	10	0.909	1	0	ritscheri												
http://apps3.awi.de/Hustedt-Diatoms/FragCl/Set1/L051_W8.0-S5.9-FragCl.NBP-1402.945-946cm.000051.png	51.2	7.7	5.9	NA	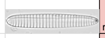	ritscheri	ritscheri	ritscheri	ritscheri	ritscheri	ritscheri		ritscheri	ritscheri	ritscheri	ritscheri	ritscheri	11	11	0	0	0	0	11	1	1	0	ritscheri												
http://apps3.awi.de/Hustedt-Diatoms/FragCl/Set1/L051_W9.0-S8.0-Wa-75b_Wa-77b.000213.png	51.2	9.1	8.0	NA	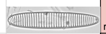	ritscheri	ritscheri	ritscheri	ritscheri	ritscheri	ritscheri	ritscheri	ritscheri	ritscheri	ritscheri	ritscheri	ritscheri	12	12	0	0	0	0	12	1	1	0	ritscheri												
http://apps3.awi.de/Hustedt-Diatoms/FragCl/Set1/L051_W10.0-S7.7-PS1768-8.000767.png	51.2	9.7	7.7	NA	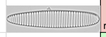	ritscheri	ritscheri	ritscheri	ritscheri	ritscheri	ritscheri		ritscheri	ritscheri	ritscheri	ritscheri	ritscheri	11	11	0	0	0	0	11	1	1	0	ritscheri												
http://apps3.awi.de/Hustedt-Diatoms/FragCl/Set1/L051_W7.0-S8.2-FragCl.ANT33-51.000153.png	51.2	6.8	8.2	8.0	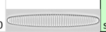	sublinearis	sublinearis	sublinearis	sublinearis	sublinearis	sublinearis			sublinearis	obliquecostata	sublinearis	sublinearis	10	0	1	9	0	0	9	0.9	3	0	sublinearis			very large but triangular keep punctae present									
http://apps3.awi.de/Hustedt-Diatoms/FragCl/Set1/L051_W7.0-S8.6-FragCl.ANT33-51.000130.png	51.3	6.8	8.6	9.6		sublinearis	sublinearis	sublinearis	sublinearis	sublinearis	sublinearis			sublinearis	obliquecostata	sublinearis	sublinearis	10	0	1	9	0	0	9	0.9	3	0	sublinearis												
http://apps3.awi.de/Hustedt-Diatoms/FragCl/Set1/L051_W8.0-SNA-FragCl.NBP-1402.999-996cm.000012.png	51.3	8.4	NA	7.1	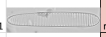	ritscheri	ritscheri	ritscheri	ritscheri	ritscheri	ritscheri	ritscheri	ritscheri	ritscheri	ritscheri	ambiguous	ritscheri	12	11	0	0	1	0	11	0.916	1	0	ritscheri											ritscheri?: specimen not in focus	
http://apps3.awi.de/Hustedt-Diatoms/FragCl/Set1/L051_W8.0-S6.0-PS1768-8.000438.png	51.3	8.5	6.0	NA	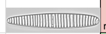	ritscheri	ritscheri	ritscheri	ambiguous	ritscheri	ritscheri		ritscheri	ambiguous	ritscheri	ritscheri	ritscheri	11	9	0	0	2	0	9	0.818	1	0	ritscheri				ritscheri/obliquecostata					ritscheri or obliquecostata			
http://apps3.awi.de/Hustedt-Diatoms/FragCl/Set1/L051_W5.0-S7.6-FragCl.ANT33-51.000364.png	51.4	5.5	7.6	NA		sublinearis	sublinearis	sublinearis	sublinearis	sublinearis	sublinearis		sublinearis	sublinearis	sublinearis	sublinearis	sublinearis	11	0	0	11	0	0	11	1	3	0	sublinearis												
http://apps3.awi.de/Hustedt-Diatoms/FragCl/Set1/L051_W8.0-S5.5-PS1768-8.000419.png	51.5	7.6	5.5	NA	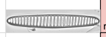	ritscheri	obliquecost	obliquecost	obliquecost	obliquecost	obliquecostata			obliquecostata	obliquecostata	obliquecostata	obliquecostata	10	1	9	0	0	0	9	0.9	2	0	obliquecostata					although not very	abberant form - could be ritscheri abbernat with obilque costae						
http://apps3.awi.de/Hustedt-Diatoms/FragCl/Set1/L052_W8.0-S6.3-FragCl.NBP-1402.945-946cm.000047.png	51.5	8.0	6.3	NA	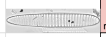	ritscheri	ritscheri	ritscheri	ritscheri	ritscheri	ritscheri	ritscheri	ritscheri	ritscheri	ritscheri	ritscheri	ritscheri	12	12	0	0	0	0	12	1	1	0	ritscheri												
http://apps3.awi.de/Hustedt-Diatoms/FragCl/Set1/L052_W8.0-S6.9-PS1768-8.000846.png	51.5	7.7	6.9	NA	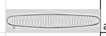	ambiguous	ritscheri	obliquecost	obliquecost	obliquecost	obliquecostata				sublinearis	obliquecostata	obliquecostata	9	1	6	1	1	0	6	0.666	2	0	obliquecostata	?ritscherii/obliquecostata				although stria not	could be confused with ritscheri, but weakly heteropo			sublinearis or obliquecostata depending on marginal poroids			
http://apps3.awi.de/Hustedt-Diatoms/FragCl/Set1/L052_W9.0-S6.3-PS1768-8.000770.png	51.5	9.0	6.3	NA	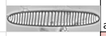	ambiguous	ritscheri	obliquecost	obliquecost	ritscheri	obliquecostata			obliquecostata	obliquecostata	obliquecostata	obliquecostata	10	2	7	0	1	0	7	0.7	2	0	obliquecostata	?ritscherii/kerguelensis					could be confused with ritscheri, but weakly heteropolar and costae oblique						
http://apps3.awi.de/Hustedt-Diatoms/FragCl/Set1/L052_W8.0-S6.1-FragCl.NBP-1402.945-946cm.000020.png	51.6	7.6	6.1	NA	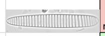	ritscheri	ritscheri	ritscheri	ritscheri	ritscheri	ritscheri	ritscheri	ritscheri	ritscheri	ritscheri	ritscheri	ritscheri	12	12	0	0	0	0	12	1	1	0	ritscheri												
http://apps3.awi.de/Hustedt-Diatoms/FragCl/Set1/L052_W6.0-S9.1-FragCl.ANT33-100.000070.png	51.7	6.1	9.1	9.3	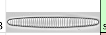	sublinearis	sublinearis	sublinearis	sublinearis	sublinearis	sublinearis	obliquecos	sublinearis	sublinearis	sublinearis	sublinearis	sublinearis	12	0	1	11	0	0	11	0.916	3	0	sublinearis												
http://apps3.awi.de/Hustedt-Diatoms/FragCl/Set1/L052_W8.0-S7.2-FragCl.NBP-1402.945-946cm.000070.png	51.7	7.6	7.2	NA	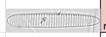	ritscheri	ritscheri	ritscheri	ritscheri	ritscheri	ritscheri		ritscheri	ritscheri	ritscheri	ritscheri	ritscheri	11	11	0	0	0	0	11	1	1	0	ritscheri												
http://apps3.awi.de/Hustedt-Diatoms/FragCl/Set1/L052_W9.0-S7.2-FragCl.ANT33-51.000133.png	51.7	8.9	7.2	7.6	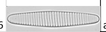	ambiguous	obliquecost	obliquecost	obliquecost	obliquecost	obliquecostata			obliquecostata	obliquecostata	obliquecostata	obliquecostata	10	0	9	0	1	0	9	0.9	2	0	obliquecostata	?ritscherii/obliquecostata											
http://apps3.awi.de/Hustedt-Diatoms/FragCl/Set1/L052_W8.0-S5.7-PS1768-8.000554.png	51.9	7.6	5.7	NA	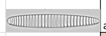	ambiguous	ritscheri	ritscheri	ritscheri	ritscheri	ritscheri	ritscheri	ritscheri	ritscheri	ritscheri	ritscheri	ritscheri	12	11	0	0	1	0	11	0.916	1	0	ritscheri	?ritscherii/kerguelensis/obliquecostata											
http://apps3.awi.de/Hustedt-Diatoms/FragCl/Set1/L052_W8.0-S7.0-FragCl.NBP-1402.960-961cm.000077.png	51.9	8.4	7.0	NA	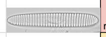	ritscheri	ritscheri	ritscheri	ritscheri	ritscheri	ritscheri		ritscheri	ritscheri	ritscheri	ritscheri	ritscheri	11	11	0	0	0	0	11	1	1	0	ritscheri												
http://apps3.awi.de/Hustedt-Diatoms/FragCl/Set1/L052_W7.0-S7.1-FragCl.NBP-1402.945-946cm.000060.png	51.9	7.0	7.1	NA	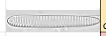	obliquecostata		ritscheri	ritscheri	ritscheri	ritscheri	ritscheri	ritscheri	ambiguous	ritscheri	ritscheri	ritscheri	11	9	1	0	1	0	9	0.818	1	0	ritscheri									probably ritzscheri but not sure			
http://apps3.awi.de/Hustedt-Diatoms/FragCl/Set1/L052_W8.0-S7.3-FragCl.NBP-1402.960-961cm.000068.png	52.0	7.9	7.3	NA	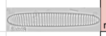	ritscheri	ritscheri	ritscheri	ritscheri	ritscheri	ritscheri		ritscheri	ritscheri	ritscheri	ritscheri	ritscheri	11	11	0	0	0	0	11	1	1	0	ritscheri												
http://apps3.awi.de/Hustedt-Diatoms/FragCl/Set1/L052_W10.0-S8.5-FragCl.ANT33-51.000096.png	52.0	10.3	8.5	7.9	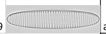	ambiguous	ritscheri	obliquecost	obliquecost	ritscheri	obliquecost	obliquecostata		ambiguous	ritscheri	obliquecostata	obliquecostata	11	3	6	0	2	0	6	0.545	2	0	obliquecostata	?ritscherii/obliquecostata				inflated valve sha	could be confused with ritscherii, but not heteropolar			obliquecostata or sublinearis?			
http://apps3.awi.de/Hustedt-Diatoms/FragCl/Set1/L052_W6.0-S5.5-PS1768-8.000961.png	52.0	5.9	5.5	NA	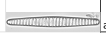	ambiguous	obliquecost	obliquecost	obliquecost	obliquecost	obliquecost	obliquecos	sublinearis	obliquecostata	obliquecostata	obliquecostata	obliquecostata	12	0	10	1	1	0	10	0.833	2	0	obliquecostata	?sublinearis/kerguelensis											

http://apps3.awi.de/Hustedt-Diatoms/FragCl/Set1/L052_W6.0-S8.0-FragCl.ANT33-51.000520.png	52.0	6.5	8.0	NA	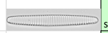	sublinearis	sublinearis	sublinearis	sublinearis	sublinearis	sublinearis	obliquecos	sublinearis	sublinearis	sublinearis	sublinearis	sublinearis	12	0	1	11	0	0	11	0.916	3	0	sublinearis							not 100 % sure (could be sublinearis?)					
http://apps3.awi.de/Hustedt-Diatoms/FragCl/Set1/L052_W7.0-S7.8-FragCl.ANT33-51.000387.png	52.0	6.6	7.8	NA	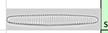	sublinearis	sublinearis	sublinearis	sublinearis	sublinearis	sublinearis	obliquecostata		sublinearis	sublinearis	sublinearis	sublinearis	11	0	1	10	0	0	10	0.909	3	0	sublinearis												
http://apps3.awi.de/Hustedt-Diatoms/FragCl/Set1/L052_W6.0-S8.6-FragCl.ANT33-51.000385.png	52.0	6.5	8.6	NA	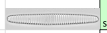	sublinearis	sublinearis	sublinearis	sublinearis	sublinearis	sublinearis	obliquecos	sublinearis	sublinearis	obliquecostata	sublinearis	sublinearis	12	0	2	10	0	0	10	0.833	3	0	sublinearis												
http://apps3.awi.de/Hustedt-Diatoms/FragCl/Set1/L052_W11.0-S8.3-FragCl.ANT33-51.000078.png	52.1	10.7	8.3	7.8	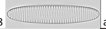	ambiguous	ritscheri	obliquecost	obliquecost	ritscheri	obliquecost	obliquecostata		ambiguous	obliquecostata	obliquecostata	obliquecostata	11	2	7	0	2	0	7	0.636	2	0	obliquecostata	?ritscherii/obliquecostata				inflated valve sha	could be confused with ritscherii, but not heteropolar			obliquecostata or sublinearis?			
http://apps3.awi.de/Hustedt-Diatoms/FragCl/Set1/L052_W9.0-S7.4-Hasle22-47.000005.png	52.1	8.9	7.4	NA	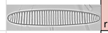	ritscheri	ritscheri	ritscheri	ritscheri	ritscheri	ritscheri		ritscheri	ritscheri	ritscheri	ritscheri	ritscheri	11	11	0	0	0	0	11	1	1	0	ritscheri												
http://apps3.awi.de/Hustedt-Diatoms/FragCl/Set1/L052_W10.0-SNA-FragCl.ANT33-51.000119.png	52.1	9.6	NA	7.6	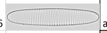	ambiguous	ritscheri	obliquecost	obliquecost	ritscheri	obliquecost	obliquecostata		ambiguous	obliquecostata	obliquecostata	obliquecostata	11	2	7	0	2	0	7	0.636	2	0	obliquecostata	?ritscherii/obliquecostata				inflated valve sha	could be confused with ritscherii, but not heteropolar			obliquecostata or sublinearis?			
http://apps3.awi.de/Hustedt-Diatoms/FragCl/Set1/L052_W9.0-S7.8-Wa-75b_Wa-77b.000023.png	52.1	8.9	7.8	NA	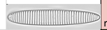	ritscheri	ritscheri	ritscheri	ritscheri	ritscheri	ritscheri		ritscheri	ritscheri	ritscheri	ritscheri	ritscheri	11	11	0	0	0	0	11	1	1	0	ritscheri												
http://apps3.awi.de/Hustedt-Diatoms/FragCl/Set1/L052_W8.0-SNA-FragCl.NBP-1402.945-946cm.000073.png	52.4	7.9	NA	6.7	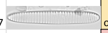	obliquecost	ritscheri	ritscheri	ritscheri	ritscheri	ritscheri		ritscheri	ritscheri	ritscheri	ritscheri	ritscheri	11	10	1	0	0	0	10	0.909	1	0	ritscheri												
http://apps3.awi.de/Hustedt-Diatoms/FragCl/Set1/L052_W8.0-S6.7-FragCl.NBP-1402.960-961cm.000101.png	52.4	8.3	6.7	NA	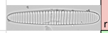	ritscheri	ritscheri	ritscheri	ritscheri	ritscheri	ritscheri	ritscheri	ritscheri	ritscheri	ritscheri	ritscheri	ritscheri	12	12	0	0	0	0	12	1	1	0	ritscheri												
http://apps3.awi.de/Hustedt-Diatoms/FragCl/Set1/L052_W6.0-S8.3-FragCl.ANT33-100.000067.png	52.5	5.8	8.3	7.8	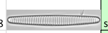	sublinearis	sublinearis	sublinearis	sublinearis	sublinearis	sublinearis	obliquecos	sublinearis	sublinearis	obliquecostata	sublinearis	sublinearis	12	0	2	10	0	0	10	0.833	3	0	sublinearis												
http://apps3.awi.de/Hustedt-Diatoms/FragCl/Set1/L053_W6.0-S9.4-PS1768-8.000440.png	52.5	6.2	9.4	NA	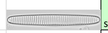	sublinearis	sublinearis	sublinearis	sublinearis	sublinearis	sublinearis		sublinearis	sublinearis	sublinearis	sublinearis	sublinearis	11	0	0	11	0	0	11	1	3	0	sublinearis												
http://apps3.awi.de/Hustedt-Diatoms/FragCl/Set1/L053_W7.0-S6.6-PS1768-8.000447.png	52.6	6.9	6.6	NA	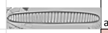	ambiguous	ambiguous	obliquecost	obliquecost	obliquecost	ritscheri			obliquecostata	obliquecostata	obliquecostata	obliquecostata	10	1	7	0	2	0	7	0.7	2	0	obliquecostata	?sublinearis/ritsc	most likely F. ritscheri										
http://apps3.awi.de/Hustedt-Diatoms/FragCl/Set1/L053_W8.0-S7.6-FragCl.NBP-1402.960-961cm.000043.png	52.6	8.3	7.6	NA	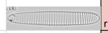	ritscheri	ritscheri	ritscheri	ritscheri	ritscheri	ritscheri	ritscheri	ritscheri	ritscheri	ritscheri	ritscheri	ritscheri	12	12	0	0	0	0	12	1	1	0	ritscheri												
http://apps3.awi.de/Hustedt-Diatoms/FragCl/Set1/L053_W9.0-S8.0-PS1768-8.000965.png	52.8	8.9	8.0	NA	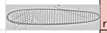	ritscheri	ritscheri	ritscheri	ritscheri	ritscheri	ritscheri	ritscheri	ritscheri	ritscheri	ritscheri	ritscheri	ritscheri	12	12	0	0	0	0	12	1	1	0	ritscheri												
http://apps3.awi.de/Hustedt-Diatoms/FragCl/Set1/L053_W8.0-S7.7-FragCl.NBP-1402.960-961cm.000095.png	52.9	8.0	7.7	NA	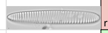	ritscheri	ritscheri	ritscheri	ritscheri	ritscheri	ritscheri	ritscheri	ritscheri	ritscheri	ritscheri	ritscheri	ritscheri	12	12	0	0	0	0	12	1	1	0	ritscheri				I am not sure… It seems tilted			could be obliquecostata: photograph not so good					
http://apps3.awi.de/Hustedt-Diatoms/FragCl/Set1/L053_W7.0-S5.9-PS1768-8.000502.png	53.0	6.6	5.9	NA	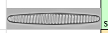	sublinearis	obliquecost	obliquecost	obliquecost	obliquecost	obliquecostata			obliquecostata	obliquecostata	obliquecostata	obliquecostata	10	0	9	1	0	0	9	0.9	2	0	obliquecostata		a llittle narrower than average										
http://apps3.awi.de/Hustedt-Diatoms/FragCl/Set1/L053_W8.0-S6.0-FragCl.NBP-1402.945-946cm.000049.png	53.0	7.7	6.0	NA	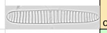	obliquecost	ritscheri	ritscheri	ritscheri	ritscheri	ritscheri	ritscheri	ritscheri	ritscheri	obliquecostata	ritscheri	ritscheri	12	10	2	0	0	0	10	0.833	1	0	ritscheri										Valve slightly broken, so it hard to determine the orier		
http://apps3.awi.de/Hustedt-Diatoms/FragCl/Set1/L053_W7.0-SNA-FragCl.ANT33-51.000514.png	53.0	6.5	NA	8.5	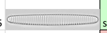	sublinearis	sublinearis	sublinearis	sublinearis	sublinearis	sublinearis	obliquecos	sublinearis	sublinearis	sublinearis	sublinearis	obliquecostata	12	0	2	10	0	0	10	0.833	3	0	sublinearis												
http://apps3.awi.de/Hustedt-Diatoms/FragCl/Set1/L053_W8.0-SNA-FragCl.NBP-1402.945-946cm.000018.png	53.0	8.3	NA	8.3	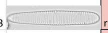	ritscheri	ritscheri	ritscheri	ritscheri	ritscheri	ritscheri	ritscheri	ritscheri	ritscheri	ritscheri	ritscheri	ritscheri	12	12	0	0	0	0	12	1	1	0	ritscheri							could be obliquecostata: photograph not so good					
http://apps3.awi.de/Hustedt-Diatoms/FragCl/Set1/L053_W10.0-S7.6-PS1768-8.000865.png	53.0	9.8	7.6	NA	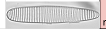	ritscheri	ritscheri	ritscheri	ritscheri	ritscheri	ritscheri		ritscheri	ritscheri	ritscheri	ritscheri	ritscheri	11	11	0	0	0	0	11	1	1	0	ritscheri												
http://apps3.awi.de/Hustedt-Diatoms/FragCl/Set1/L053_W6.0-S8.1-FragCl.ANT33-51.000528.png	53.1	6.0	8.1	NA	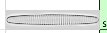	sublinearis	sublinearis	sublinearis	sublinearis	sublinearis	sublinearis		sublinearis	sublinearis	obliquecostata	sublinearis	sublinearis	11	0	1	10	0	0	10	0.909	3	0	sublinearis												
http://apps3.awi.de/Hustedt-Diatoms/FragCl/Set1/L053_W5.0-S8.5-PS1768-8.000428.png	53.1	5.4	8.5	NA	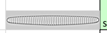	sublinearis	sublinearis	sublinearis	sublinearis	sublinearis	sublinearis		sublinearis	sublinearis	obliquecostata	sublinearis	sublinearis	11	0	1	10	0	0	10	0.909	3	0	sublinearis												
http://apps3.awi.de/Hustedt-Diatoms/FragCl/Set1/L053_W7.0-S8.1-PS1768-8.000778.png	53.2	6.6	8.1	NA	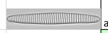	ambiguous	obliquecost	obliquecost	obliquecost	obliquecost	obliquecostata			ambiguous	obliquecostata	obliquecostata	obliquecostata	10	0	8	0	2	0	8	0.8	2	0	obliquecostata	?sublinearis/ritscherii		twisted specimen again, may mean something			abberant form			obliquecostata or sublinearis?			
http://apps3.awi.de/Hustedt-Diatoms/FragCl/Set1/L053_W6.0-S6.1-PS1768-8.000458.png	53.2	6.1	6.1	NA	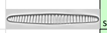	sublinearis	obliquecost	sublinearis	obliquecost	obliquecost	obliquecostata		sublinearis	obliquecostata	obliquecostata	sublinearis	obliquecostata	11	0	7	4	0	0	7	0.636	2	0	obliquecostata		a little narrower than average				could be sublinearis						
http://apps3.awi.de/Hustedt-Diatoms/FragCl/Set1/L053_W6.0-S8.1-PS1768-8.000503.png	53.3	6.4	8.1	NA	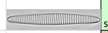	sublinearis	obliquecost	sublinearis	obliquecost	obliquecost	obliquecostata		sublinearis	ambiguous	obliquecostata	obliquecostata	obliquecostata	11	0	7	3	1	0	7	0.636	2	0	obliquecostata		a little narrower than average							obliquecostata or sublinearis?			
http://apps3.awi.de/Hustedt-Diatoms/FragCl/Set1/L053_W8.0-S5.9-FragCl.NBP-1402.945-946cm.000016.png	53.4	7.6	5.9	NA	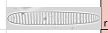	ritscheri	ritscheri	ritscheri	ritscheri	ritscheri	ritscheri	ritscheri	ritscheri	ritscheri	ritscheri	ritscheri	sublinearis	12	11	0	1	0	0	11	0.916	1	0	ritscheri												
http://apps3.awi.de/Hustedt-Diatoms/FragCl/Set1/L053_W8.0-S6.3-PS1768-8.000762.png	53.4	8.3	6.3	NA	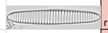	ritscheri	ritscheri	ritscheri	obliquecost	obliquecost	obliquecost	obliquecostata		ambiguous	obliquecostata	obliquecostata	ritscheri	11	4	6	0	1	0	6	0.545	2	0	obliquecostata					not particularly heteroplolar, hint of slanted striae				obliquecostata or sublinearis?			
http://apps3.awi.de/Hustedt-Diatoms/FragCl/Set1/L053_W8.0-SNA-FragCl.NBP-1402.945-946cm.000010.png	53.4	8.0	NA	7.8	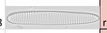	ritscheri	ritscheri	ritscheri	ritscheri	ritscheri	ritscheri	ritscheri	ritscheri	ritscheri	ritscheri	ritscheri	ritscheri	12	12	0	0	0	0	12	1	1	0	ritscheri												
http://apps3.awi.de/Hustedt-Diatoms/FragCl/Set1/L053_W10.0-SNA-FragCl.ANT33-76.000001.png	53.5	9.6	NA	8.8	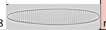	ritscheri	ritscheri	ritscheri	ritscheri	ritscheri	ritscheri		ritscheri	ritscheri	ritscheri	ritscheri	ritscheri	11	11	0	0	0	0	11	1	1	0	ritscheri												
http://apps3.awi.de/Hustedt-Diatoms/FragCl/Set1/L054_W9.0-S7.4-PS1768-8.000432.png	53.6	8.7	7.4	NA	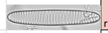	ritscheri	ritscheri	obliquecost	obliquecost	obliquecost	obliquecostata			obliquecostata	obliquecostata	obliquecostata	obliquecostata	10	2	8	0	0	0	8	0.8	2	0	obliquecostata					although I almost	could be ritscheri, but inflation is middle						
http://apps3.awi.de/Hustedt-Diatoms/FragCl/Set1/L054_W8.0-S5.2-FragCl.NBP-1402.945-946cm.000015.png	53.6	7.6	5.2	NA	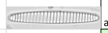	ambiguous	ritscheri		obliquecost	obliquecost	ritscheri	obliquecostata		obliquecostata	obliquecostata	obliquecostata	kerguelensis	10	2	6	0	1	1	6	0.6	2	0	obliquecostata	?obliquecostata/	oblique striae	tilted specimen, need more pics for		difficult photo	image is oblique diifcult to be sure		here I'm not sure				
http://apps3.awi.de/Hustedt-Diatoms/FragCl/Set1/L054_W7.0-S9.0-FragCl.ANT33-100.000109.png	53.7	6.5	9.0	9.6	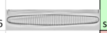	sublinearis	sublinearis	sublinearis	sublinearis	obliquecost	obliquecost	obliquecos	sublinearis	ambiguous	obliquecostata	obliquecostata	sublinearis	12	0	5	6	1	0	6	0.5	3	0	sublinearis						could be confused with sublineata due to indiscernab			sublinearis if marginal poroids were more distinct on both sides			
http://apps3.awi.de/Hustedt-Diatoms/FragCl/Set1/L054_W8.0-S7.4-FragCl.NBP-1402.960-961cm.000045.png	53.7	8.4	7.4	NA	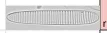	ritscheri	ritscheri	ritscheri	ritscheri	ritscheri	ritscheri	ritscheri	ritscheri	ritscheri	ritscheri	ritscheri	ritscheri	12	12	0	0	0	0	12	1	1	0	ritscheri												
http://apps3.awi.de/Hustedt-Diatoms/FragCl/Set1/L054_W9.0-S7.0-FragCl.ANT33-51.000076.png	53.9	8.5	7.0	6.3	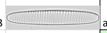	ambiguous	ritscheri	obliquecost	obliquecost	ritscheri	obliquecost	obliquecostata		obliquecostata	obliquecostata	obliquecostata	obliquecostata	11	2	8	0	1	0	8	0.727	2	0	obliquecostata	?ritscherii/obliquecostata		isopolar, max width at equan distance from apices, ap			could be confused with ritscherii, but not heteropolar enough and costae oblique, otherwise sizes similar between the two spe						
http://apps3.awi.de/Hustedt-Diatoms/FragCl/Set1/L054_W6.0-S7.3-FragCl.ANT33-76.000070.png	53.9	6.0	7.3	NA	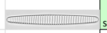	sublinearis	sublinearis	sublinearis	sublinearis	sublinearis	sublinearis	obliquecos	sublinearis	sublinearis	sublinearis	sublinearis	sublinearis	12	0	1	11	0	0	11	0.916	3	0	sublinearis												
http://apps3.awi.de/Hustedt-Diatoms/FragCl/Set1/L054_W7.0-S5.2-PS1768-8.000490.png	54.0	7.5	5.2	NA	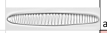	ambiguous	ritscheri		obliquecost	obliquecost	ritscheri	obliquecos	ritscheri	ambiguous	obliquecostata	obliquecostata	obliquecostata	11	3	6	0	2	0	6	0.545	2	0	obliquecostata	?sublinearis/oblic	oblique striae	tilted specimen, need more pics for		difficult photo	is oblique-viewed so could be obliquecostata			probably obliquecostata			
http://apps3.awi.de/Hustedt-Diatoms/FragCl/Set1/L054_W9.0-SNA-FragCl.NBP-1402.999-996cm.000011.png	54.1	8.9	NA	9.6	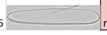	ritscheri	ritscheri	ritscheri	ritscheri	ritscheri	ritscheri		ritscheri	ritscheri	ritscheri	ritscheri	ritscheri	11	11	0	0	0	0	11	1	1	0	ritscheri												

http://apps3.awi.de/Hustedt-Diatoms/FragCl/Set1/L054_W9.0-S6.9-FragCl.NBP-1402.960-961cm.000028.png	54.2	9.1	6.9	NA	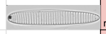	ritscheri	ritscheri	ritscheri	ritscheri	ritscheri	ritscheri	ritscheri	ritscheri	ritscheri	ritscheri	ritscheri	ritscheri	12	12	0	0	0	0	12	1	1	0	ritscheri												
http://apps3.awi.de/Hustedt-Diatoms/FragCl/Set1/L054_W8.0-S6.4-FragCl.NBP-1402.945-946cm.000024.png	54.3	8.3	6.4	NA	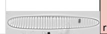	ritscheri	ritscheri	ritscheri	ritscheri	ritscheri	ritscheri	ritscheri	ritscheri	ritscheri	ritscheri	ritscheri	ritscheri	12	12	0	0	0	0	12	1	1	0	ritscheri												
http://apps3.awi.de/Hustedt-Diatoms/FragCl/Set1/L054_W9.0-S5.9-PS1768-8.000863.png	54.3	9.0	5.9	NA	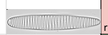	ritscheri	ritscheri	ritscheri	ritscheri	ritscheri	ritscheri	ritscheri	ritscheri	ritscheri	ritscheri	ritscheri	ritscheri	12	12	0	0	0	0	12	1	1	0	ritscheri												
http://apps3.awi.de/Hustedt-Diatoms/FragCl/Set1/L054_W6.0-S7.1-FragCl.ANT33-51.000523.png	54.4	6.3	7.1	NA	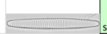	sublinearis	sublinearis	sublinearis	sublinearis	sublinearis	sublinearis	obliquecos	sublinearis	sublinearis	sublinearis	sublinearis	sublinearis	12	0	1	11	0	0	11	0.916	3	0	sublinearis												
http://apps3.awi.de/Hustedt-Diatoms/FragCl/Set1/L054_W9.0-S6.7-PS1768-8.000414.png	54.4	8.7	6.7	NA	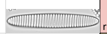	ritscheri	ritscheri	obliquecost	obliquecost	ritscheri	ritscheri	obliquecostata		obliquecostata	obliquecostata	obliquecostata	obliquecostata	11	4	7	0	0	0	7	0.636	2	0	obliquecostata			seems isopolar even though apical costae not very deviated on upper apex, need more pics for identification									
http://apps3.awi.de/Hustedt-Diatoms/FragCl/Set1/L055_W8.0-S5.7-FragCl.NBP-1402.945-946cm.000022.png	54.8	8.0	5.7	NA		obliquecost	ritscheri	ritscheri	ritscheri	ritscheri	ritscheri		ritscheri	ritscheri	ritscheri	ritscheri	ritscheri	11	10	1	0	0	0	10	0.909	1	0	ritscheri												
http://apps3.awi.de/Hustedt-Diatoms/FragCl/Set1/L055_W6.0-S7.9-PS1768-8.000476.png	54.9	5.5	7.9	NA	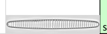	sublinearis	sublinearis	sublinearis	sublinearis	sublinearis	sublinearis		sublinearis	sublinearis	obliquecostata	sublinearis	sublinearis	11	0	1	10	0	0	10	0.909	3	0	sublinearis												
http://apps3.awi.de/Hustedt-Diatoms/FragCl/Set1/L055_W9.0-S8.8-FragCl.NBP-1402.960-961cm.000009.png	54.9	9.3	8.8	NA	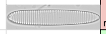	ritscheri	ritscheri	ritscheri	ritscheri	ritscheri	ritscheri		ritscheri	ritscheri	ritscheri	ritscheri	ritscheri	11	11	0	0	0	0	11	1	1	0	ritscheri												
http://apps3.awi.de/Hustedt-Diatoms/FragCl/Set1/L055_W6.0-SNA-PS1768-8.000761.png	54.9	5.8	NA	8.1	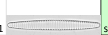	sublinearis	sublinearis	sublinearis	sublinearis	sublinearis	sublinearis		sublinearis	sublinearis	obliquecostata	sublinearis	sublinearis	11	0	1	10	0	0	10	0.909	3	0	sublinearis												
http://apps3.awi.de/Hustedt-Diatoms/FragCl/Set1/L055_W10.0-S6.2-PS1768-8.000525.png	54.9	9.8	6.2	NA	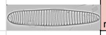	ritscheri	ritscheri	ritscheri	ritscheri	ritscheri	ritscheri		ritscheri	ritscheri	ritscheri	ritscheri	ritscheri	11	11	0	0	0	0	11	1	1	0	ritscheri												
http://apps3.awi.de/Hustedt-Diatoms/FragCl/Set1/L055_W7.0-S6.7-PS1768-8.000534.png	55.0	6.6	6.7	NA	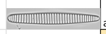	ambiguous	ritscheri	ritscheri	ambiguous	ritscheri	ritscheri		ritscheri	ambiguous	obliquecostata	obliquecostata	obliquecostata	11	5	3	0	3	0	5	0.454	1	0	ritscheri	?sublinearis/kerguelensis/obliquecc		heteropolar, stro	ritscheri/obliquecostata					ritscheri or obliquecostata			???? Heteropolar
http://apps3.awi.de/Hustedt-Diatoms/FragCl/Set1/L055_W8.0-S5.2-FragCl.NBP-1402.945-946cm.000065.png	55.2	7.7	5.2	NA	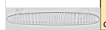	obliquecost	ritscheri	ritscheri	ritscheri	ritscheri	ritscheri			ritscheri	ritscheri	obliquecostata	ritscheri	10	8	2	0	0	0	8	0.8	1	0	ritscheri			heteropolar, stro	I am not sure… It seems tilted								
http://apps3.awi.de/Hustedt-Diatoms/FragCl/Set1/L055_W11.0-SNA-FragCl.ANT33-51.000107.png	55.2	10.5	NA	9.3	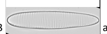	ambiguous	ritscheri	ritscheri	ritscheri	ritscheri	ritscheri		ritscheri	ritscheri	ritscheri	ritscheri	ritscheri	11	10	0	0	1	0	10	0.909	1	0	ritscheri	?ritscherii/obliquecostata											
http://apps3.awi.de/Hustedt-Diatoms/FragCl/Set1/L055_W7.0-S7.0-FragCl.ANT33-76.000057.png	55.3	7.4	7.0	NA	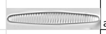	ambiguous	obliquecostata		obliquecost	obliquecost	obliquecostata			ambiguous	obliquecostata	obliquecostata	obliquecostata	9	0	7	0	2	0	7	0.777	2	0	obliquecostata	?sublinearis/ritscherii		tilted specimen, r	I am not sure… It seems tilted		diatom is not flat though			obliquecostata or sublinearis?			
http://apps3.awi.de/Hustedt-Diatoms/FragCl/Set1/L056_W8.0-S6.4-PS1768-8.000967.png	55.6	7.7	6.4	NA	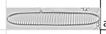	ambiguous	ritscheri	peragallii	obliquecost	obliquecost	peragalli		peragallii	ambiguous	obliquecostata	obliquecostata	obliquecostata	11	1	5	0	2	3	5	0.454	2	0	obliquecostata	?ritscherii/peragallii/linearis		overall shape makes me think of F. peragallii but my s			cf. peragalli, but costae oblique, not target species			probably obliquecostata			
http://apps3.awi.de/Hustedt-Diatoms/FragCl/Set1/L056_W9.0-S7.2-FragCl.ANT33-51.000011.png	55.7	9.5	7.2	7.1	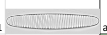	ambiguous	ritscheri	ritscheri	obliquecost	ritscheri	obliquecostata		obliquecostata	ambiguous	obliquecostata	obliquecostata	obliquecostata	11	3	6	0	2	0	6	0.545	2	0	obliquecostata	?ritscherii/obliquecostata		but not sure, seems heteropolar			could be confused with ritscherii, but not heteropolar			probably obliquecostata			
http://apps3.awi.de/Hustedt-Diatoms/FragCl/Set1/L056_W7.0-SNA-FragCl.ANT33-51.000355.png	55.7	6.7	NA	10.1	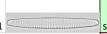	sublinearis	sublinearis	sublinearis	sublinearis	sublinearis	sublinearis		sublinearis	sublinearis	sublinearis	sublinearis	sublinearis	11	0	0	11	0	0	11	1	3	0	sublinearis												
http://apps3.awi.de/Hustedt-Diatoms/FragCl/Set1/L056_W8.0-S7.5-FragCl.NBP-1402.960-961cm.000037.png	55.9	8.2	7.5	NA	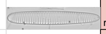	ritscheri	ritscheri	ritscheri	ritscheri	ritscheri	ritscheri		ritscheri	ritscheri	ritscheri	ritscheri	ritscheri	11	11	0	0	0	0	11	1	1	0	ritscheri												
http://apps3.awi.de/Hustedt-Diatoms/FragCl/Set1/L056_W9.0-S7.6-PS1768-8.000429.png	56.0	9.4	7.6	NA	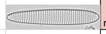	ritscheri	ritscheri	ritscheri	ritscheri	ritscheri	ritscheri	ritscheri	ritscheri	ritscheri	ritscheri	ritscheri	ritscheri	12	12	0	0	0	0	12	1	1	0	ritscheri												
http://apps3.awi.de/Hustedt-Diatoms/FragCl/Set1/L056_W7.0-S5.2-PS1768-8.000968.png	56.0	7.5	5.2	NA	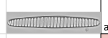	ambiguous	obliquecost	obliquecost	obliquecost	obliquecost	obliquecostata		sublinearis	obliquecostata	obliquecostata	obliquecostata	obliquecostata	11	0	9	1	1	0	9	0.818	2	0	obliquecostata	?kerguelensis/obliquecostata/ritscherii											
http://apps3.awi.de/Hustedt-Diatoms/FragCl/Set1/L056_W7.0-S6.6-FragCl.NBP-1402.945-946cm.000041.png	56.2	7.5	6.6	NA	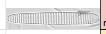	ritscheri	ritscheri	ritscheri	ritscheri	ritscheri	ritscheri		ritscheri	ritscheri	ritscheri	ritscheri	ritscheri	11	11	0	0	0	0	11	1	1	0	ritscheri												
http://apps3.awi.de/Hustedt-Diatoms/FragCl/Set1/L056_W7.0-S6.1-FragCl.NBP-1402.945-946cm.000074.png	56.3	7.3	6.1	NA	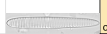	obliquecost	ritscheri	ritscheri	ritscheri	ritscheri	ritscheri		ritscheri	ritscheri	ritscheri	ritscheri	ritscheri	11	10	1	0	0	0	10	0.909	1	0	ritscheri				I am not sure… It seems tilted								
http://apps3.awi.de/Hustedt-Diatoms/FragCl/Set1/L056_W8.0-S8.1-FragCl.NBP-1402.960-961cm.000072.png	56.4	8.3	8.1	NA	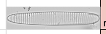	ritscheri	ritscheri	ritscheri	ritscheri	ritscheri	ritscheri		ritscheri	ritscheri	ritscheri	ritscheri	ritscheri	11	11	0	0	0	0	11	1	1	0	ritscheri										F. ritscheri although 56-57 micrometers (apical axis) st		
http://apps3.awi.de/Hustedt-Diatoms/FragCl/Set1/L057_W8.0-S7.0-FragCl.NBP-1402.960-961cm.000099.png	56.7	8.1	7.0	NA	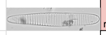	ritscheri	ritscheri	ritscheri	ritscheri	ritscheri	ritscheri	ritscheri	ritscheri	ritscheri	ritscheri	obliquecostata	ritscheri	12	11	1	0	0	0	11	0.916	1	0	ritscheri												
http://apps3.awi.de/Hustedt-Diatoms/FragCl/Set1/L057_W10.0-S8.8-FragCl.ANT33-51.000007.png	56.7	10.0	8.8	9.3	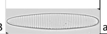	ambiguous	ritscheri	ritscheri	ritscheri	ritscheri	ritscheri	ritscheri	ritscheri	ritscheri	ritscheri	obliquecostata	ritscheri	12	10	1	0	1	0	10	0.833	1	0	ritscheri	?ritscherii/obliquecostata											
http://apps3.awi.de/Hustedt-Diatoms/FragCl/Set1/L057_W8.0-S5.7-FragCl.NBP-1402.945-946cm.000068.png	56.8	8.0	5.7	NA	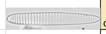	obliquecost	ritscheri	ritscheri	ritscheri	ritscheri	ritscheri	ritscheri	ritscheri	ritscheri	ritscheri	ritscheri	ritscheri	12	11	1	0	0	0	11	0.916	1	0	ritscheri												
http://apps3.awi.de/Hustedt-Diatoms/FragCl/Set1/L057_W10.0-S8.5-FragCl.ANT33-51.000105.png	56.8	10.1	8.5	8.5	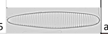	ambiguous	ritscheri	ritscheri	ritscheri	ritscheri	ritscheri	ritscheri	ritscheri	ritscheri	ritscheri	ritscheri	ritscheri	12	11	0	0	1	0	11	0.916	1	0	ritscheri	?ritscherii/obliquecostata											
http://apps3.awi.de/Hustedt-Diatoms/FragCl/Set1/L057_W9.0-S6.9-FragCl.NBP-1402.999-996cm.000015.png	56.8	9.2	6.9	NA	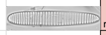	ritscheri	ritscheri	ritscheri	ritscheri	ritscheri	ritscheri	ritscheri	ritscheri	ritscheri	ritscheri	ritscheri	ritscheri	12	12	0	0	0	0	12	1	1	0	ritscheri												
http://apps3.awi.de/Hustedt-Diatoms/FragCl/Set1/L057_W9.0-SNA-FragCl.NBP-1402.960-961cm.000051.png	56.9	8.7	NA	6.0	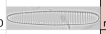	ritscheri	ritscheri	ritscheri	ritscheri	ritscheri	ritscheri	ritscheri	ritscheri	ritscheri	ritscheri	ritscheri	ritscheri	12	12	0	0	0	0	12	1	1	0	ritscheri							not 100% sure could also be obliquecostata					
http://apps3.awi.de/Hustedt-Diatoms/FragCl/Set1/L057_W8.0-S5.9-FragCl.NBP-1402.945-946cm.000027.png	56.9	8.4	5.9	NA	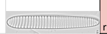	ritscheri	ritscheri	ritscheri	ritscheri	ritscheri	ritscheri	ritscheri	ritscheri	ritscheri	ritscheri	ritscheri	ritscheri	12	12	0	0	0	0	12	1	1	0	ritscheri												
http://apps3.awi.de/Hustedt-Diatoms/FragCl/Set1/L057_W7.0-S7.0-PS1768-8.000488.png	57.0	7.1	7.0	NA	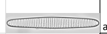	ambiguous	ambiguous	obliquecost	obliquecost	sublinearis	obliquecost	obliquecos	sublinearis	ambiguous	sublinearis	obliquecostata	obliquecostata	12	0	6	3	3	0	6	0.5	2	0	obliquecostata	?sublinearis/oblic	obliquecostata - ritscheri			although starting to have obliquecostata valve outline, striae don't appe				obliquecostata or sublinearis?			
http://apps3.awi.de/Hustedt-Diatoms/FragCl/Set1/L057_W7.0-S5.8-PS1768-8.000521.png	57.1	7.0	5.8	NA	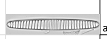	ambiguous	ambiguous	obliquecost	obliquecost	obliquecost	obliquecost	obliquecos	sublinearis	obliquecostata	obliquecostata	obliquecostata	obliquecostata	12	0	9	1	2	0	9	0.75	2	0	obliquecostata	?sublinearis/oblic	obliquecostata - ritscheri										
http://apps3.awi.de/Hustedt-Diatoms/FragCl/Set1/L057_W10.0-S7.7-FragCl.ANT33-51.000104.png	57.1	9.6	7.7	8.1	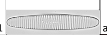	ambiguous	ritscheri	obliquecost	obliquecost	obliquecost	obliquecost	obliquecos	obliquecostata	ambiguous	obliquecostata	obliquecostata	obliquecostata	12	1	9	0	2	0	9	0.75	2	0	obliquecostata	?ritscherii/obliquecostata					inflated central region			obliquecostata or sublinearis?			
http://apps3.awi.de/Hustedt-Diatoms/FragCl/Set1/L057_W9.0-S8.7-PS1768-8.000422.png	57.1	9.5	8.7	NA	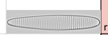	ritscheri	ritscheri	ritscheri	ritscheri	ritscheri	ritscheri	ritscheri	ritscheri	ritscheri	ritscheri	ritscheri	ritscheri	12	12	0	0	0	0	12	1	1	0	ritscheri												
http://apps3.awi.de/Hustedt-Diatoms/FragCl/Set1/L057_W9.0-S7.0-FragCl.NBP-1402.960-961cm.000067.png	57.2	8.6	7.0	NA	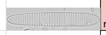	ritscheri	ritscheri	ritscheri	ritscheri	ritscheri	ritscheri	ritscheri	ritscheri	ambiguous	ritscheri	ritscheri	ritscheri	12	11	0	0	1	0	11	0.916	1	0	ritscheri							not 100% sure could also be oblique		ritscheri or sublinearis			
http://apps3.awi.de/Hustedt-Diatoms/FragCl/Set1/L057_W8.0-S6.8-FragCl.NBP-1402.945-946cm.000029.png	57.2	8.1	6.8	NA	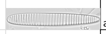	ambiguous	ritscheri	ritscheri	ritscheri	ritscheri	ritscheri	ritscheri	ritscheri	ritscheri	ritscheri	ritscheri	ritscheri	12	11	0	0	1	0	11	0.916	1	0	ritscheri	?ritscheri/obliquecostata											
http://apps3.awi.de/Hustedt-Diatoms/FragCl/Set1/L057_W7.0-SNA-FragCl.ANT33-51.000521.png	57.3	7.4	NA	8.5	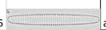	ambiguous	sublinearis	sublinearis	sublinearis	sublinearis	sublinearis	obliquecos	peragallii	sublinearis	sublinearis	sublinearis	sublinearis	12	0	1	9	1	1	9	0.75	3	0	sublinearis	?sublinearis/ritscherii						not 100% sure could be sublinearis					

http://apps3.awi.de/Hustedt-Diatoms/FragCl/Set1/L057_W9.0-S6.1-PS1768-8.000491.png	57.3	8.7	6.1	NA	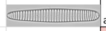	ambiguous	ritscheri	ritscheri	ritscheri	ritscheri	ritscheri	ritscheri	ritscheri	ritscheri	ritscheri	obliquecostata	ritscheri	12	10	1	0	1	0	10	0.833	1	0	ritscheri	?ritscherii/obliquecostata						
http://apps3.awi.de/Hustedt-Diatoms/FragCl/Set1/L058_W8.0-S5.9-FragCl.NBP-1402.945-946cm.000054.png	57.6	8.0	5.9	NA	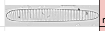	ritscheri	ritscheri	ritscheri	ritscheri	ritscheri	ritscheri		ritscheri	ritscheri	ritscheri	ritscheri	ritscheri	11	11	0	0	0	0	11	1	1	0	ritscheri							
http://apps3.awi.de/Hustedt-Diatoms/FragCl/Set1/L058_W8.0-S6.6-FragCl.NBP-1402.945-946cm.000052.png	57.6	7.7	6.6	NA	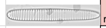	ritscheri	ritscheri	ritscheri	ritscheri	ritscheri	ritscheri		obliquecostata	ritscheri	ritscheri	ritscheri	ritscheri	11	10	1	0	0	0	10	0.909	1	0	ritscheri							
http://apps3.awi.de/Hustedt-Diatoms/FragCl/Set1/L058_W7.0-S5.7-PS1768-8.000452.png	57.7	7.2	5.7	NA	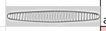	ambiguous	ambiguous	obliquecost	obliquecost	obliquecost	obliquecost	obliquecos	sublinearis	obliquecostata	obliquecostata	obliquecostata	obliquecostata	12	0	9	1	2	0	9	0.75	2	0	obliquecostata	?sublinearis/oblic	most like F. obliquecostata		abberant form			
http://apps3.awi.de/Hustedt-Diatoms/FragCl/Set1/L058_W10.0-S6.9-PS1768-8.000774.png	58.1	9.9	6.9	NA	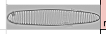	ritscheri	ritscheri	ritscheri	ritscheri	ritscheri	obliquecost	ritscheri	ritscheri	ritscheri	ritscheri	obliquecostata	ritscheri	12	10	2	0	0	0	10	0.833	1	0	ritscheri				could be confused with ritscheri due to strong hetropolarity, but costae weakly oblique, and width over-inflated in the central			
http://apps3.awi.de/Hustedt-Diatoms/FragCl/Set1/L058_W9.0-S6.3-PS1768-8.000520.png	58.1	8.6	6.3	NA	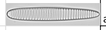	ambiguous	ritscheri	ritscheri	ritscheri	ritscheri	ritscheri		obliquecostata	ritscheri	ritscheri	ritscheri	ritscheri	11	9	1	0	1	0	9	0.818	1	0	ritscheri	?ritscherii/obliquecostata						
http://apps3.awi.de/Hustedt-Diatoms/FragCl/Set1/L058_W7.0-S5.1-PS1768-8.000763.png	58.2	6.8	5.1	NA	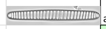	ambiguous	ambiguous	obliquecost	obliquecost	obliquecost	obliquecost	obliquecos	sublinearis	obliquecostata	obliquecostata	obliquecostata	obliquecostata	12	0	9	1	2	0	9	0.75	2	0	obliquecostata	?sublinearis/kerg	most like F. obliquecostata					
http://apps3.awi.de/Hustedt-Diatoms/FragCl/Set1/L058_W6.0-S8.5-FragCl.ANT33-51.000525.png	58.2	6.4	8.5	NA	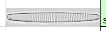	sublinearis	sublinearis	sublinearis	sublinearis	sublinearis	sublinearis	obliquecos	sublinearis	ambiguous	sublinearis	sublinearis	sublinearis	12	0	1	10	1	0	10	0.833	3	0	sublinearis						sublinearis if marginal poroids were more distinct on both sides	
http://apps3.awi.de/Hustedt-Diatoms/FragCl/Set1/L058_W9.0-S5.6-PS1768-8.000859.png	58.3	9.1	5.6	NA	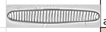	ambiguous	ritscheri	obliquecost	obliquecost	obliquecost	obliquecostata		sublinearis	obliquecostata	obliquecostata	obliquecostata	obliquecostata	11	1	8	1	1	0	8	0.727	2	0	obliquecostata	?ritscherii/obliquecostata		difficult	abberant form			
http://apps3.awi.de/Hustedt-Diatoms/FragCl/Set1/L058_W9.0-S6.4-FragCl.NBP-1402.960-961cm.000074.png	58.3	8.8	6.4	NA	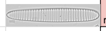	ritscheri	ritscheri	ritscheri	ritscheri	ritscheri	ritscheri		obliquecostata	ritscheri	ritscheri	ritscheri	ritscheri	11	10	1	0	0	0	10	0.909	1	0	ritscheri							
http://apps3.awi.de/Hustedt-Diatoms/FragCl/Set1/L058_W9.0-S8.1-PS1768-8.000539.png	58.3	9.4	8.1	NA	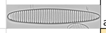	ambiguous	ritscheri	ritscheri	ritscheri	ritscheri	ritscheri		obliquecostata	ritscheri	ritscheri	ritscheri	ritscheri	11	9	1	0	1	0	9	0.818	1	0	ritscheri	?ritscherii/obliquecostata						
http://apps3.awi.de/Hustedt-Diatoms/FragCl/Set1/L058_W8.0-S6.2-FragCl.NBP-1402.945-946cm.000061.png	58.3	7.8	6.2	NA	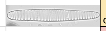	obliquecost	ritscheri	ritscheri	ritscheri	ritscheri	ritscheri		obliquecostata	ritscheri	ritscheri	obliquecostata	ritscheri	11	8	3	0	0	0	8	0.727	1	0	ritscheri							
http://apps3.awi.de/Hustedt-Diatoms/FragCl/Set1/L058_W8.0-S8.1-FragCl.NBP-1402.999-996cm.000010.png	58.5	8.4	8.1	NA	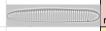	ritscheri	ritscheri	ritscheri	ritscheri	ritscheri	ritscheri		obliquecostata	ritscheri	ritscheri	ritscheri	ritscheri	11	10	1	0	0	0	10	0.909	1	0	ritscheri							
http://apps3.awi.de/Hustedt-Diatoms/FragCl/Set1/L058_W8.0-S5.6-FragCl.NBP-1402.945-946cm.000062.png	58.5	7.9	5.6	NA	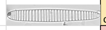	obliquecost	ritscheri	ritscheri	ritscheri	ritscheri	ritscheri	ritscheri	ritscheri	ritscheri	ritscheri	ritscheri	ritscheri	12	11	1	0	0	0	11	0.916	1	0	ritscheri					not 100% sure could also be obliquecostata		
http://apps3.awi.de/Hustedt-Diatoms/FragCl/Set1/L059_W10.0-S9.0-FragCl.ANT33-76.000049.png	58.5	10.2	9.0	NA	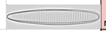	ritscheri	ritscheri	ritscheri	ritscheri	ritscheri	ritscheri		obliquecostata	ritscheri	ritscheri	ritscheri	ritscheri	11	10	1	0	0	0	10	0.909	1	0	ritscheri							
http://apps3.awi.de/Hustedt-Diatoms/FragCl/Set1/L059_W10.0-S8.8-PS1768-8.000436.png	58.7	9.7	8.8	NA	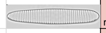	ritscheri	ritscheri	ritscheri	ritscheri	ritscheri	ritscheri		obliquecostata	ritscheri	ritscheri	ritscheri	ritscheri	11	10	1	0	0	0	10	0.909	1	0	ritscheri							
http://apps3.awi.de/Hustedt-Diatoms/FragCl/Set1/L059_W9.0-S7.4-FragCl.NBP-1402.960-961cm.000073.png	59.1	8.6	7.4	NA	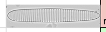	ritscheri	ritscheri	ritscheri	ritscheri	ritscheri	ritscheri	ritscheri	obliquecostata	ritscheri	ritscheri	ritscheri	ritscheri	12	11	1	0	0	0	11	0.916	1	0	ritscheri							
http://apps3.awi.de/Hustedt-Diatoms/FragCl/Set1/L059_W6.0-S8.3-FragCl.ANT33-51.000151.png	59.1	6.0	8.3	8.2	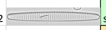	sublinearis	sublinearis	sublinearis	sublinearis	sublinearis	sublinearis		sublinearis	sublinearis	sublinearis	sublinearis	sublinearis	11	0	0	11	0	0	11	1	3	0	sublinearis							
http://apps3.awi.de/Hustedt-Diatoms/FragCl/Set1/L059_W9.0-S9.1-FragCl.ANT33-51.000122.png	59.2	9.4	9.1	8.9	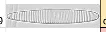	obliquecost	ritscheri	ritscheri	ritscheri	ritscheri	ritscheri	ritscheri	obliquecostata	ritscheri	ritscheri	ritscheri	ritscheri	12	10	2	0	0	0	10	0.833	1	0	ritscheri							
http://apps3.awi.de/Hustedt-Diatoms/FragCl/Set1/L059_W8.0-S7.2-FragCl.NBP-1402.960-961cm.000050.png	59.2	8.5	7.2	NA	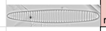	ritscheri	ritscheri	ritscheri	ritscheri	ritscheri	ritscheri		obliquecostata	ritscheri	ritscheri	ritscheri	ritscheri	11	10	1	0	0	0	10	0.909	1	0	ritscheri							
http://apps3.awi.de/Hustedt-Diatoms/FragCl/Set1/L059_W7.0-S5.7-PS1768-8.000787.png	59.2	7.0	5.7	NA		ambiguous	obliquecost	obliquecost	obliquecost	obliquecost	obliquecostata		obliquecostata	obliquecostata	obliquecostata	obliquecostata	obliquecostata	11	0	10	0	1	0	10	0.909	2	0	obliquecostata	?sublinearis/kerguelensis			image is oblique difficult to be sure			
http://apps3.awi.de/Hustedt-Diatoms/FragCl/Set1/L059_W10.0-SNA-FragCl.ANT33-51.000025.png	59.3	9.7	NA	9.5	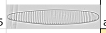	ambiguous	ritscheri	ritscheri	ritscheri	ritscheri	ritscheri		obliquecostata	ritscheri	ritscheri	ritscheri	ritscheri	11	9	1	0	1	0	9	0.818	1	0	ritscheri	?ritscherii/obliquecostata						
http://apps3.awi.de/Hustedt-Diatoms/FragCl/Set1/L059_W9.0-S6.6-FragCl.ANT33-51.000085.png	59.3	8.9	6.6	7.0	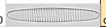	obliquecost	ritscheri	obliquecost	obliquecost	ritscheri	obliquecost	obliquecos	obliquecostata	obliquecostata	obliquecostata	obliquecostata	obliquecostata	12	2	10	0	0	0	10	0.833	2	0	obliquecostata						sublinearis if marginal poroids were more distinct on both sides	
http://apps3.awi.de/Hustedt-Diatoms/FragCl/Set1/L059_W8.0-S8.7-FragCl.NBP-1402.960-961cm.000093.png	59.3	8.4	8.7	NA	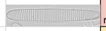	ritscheri	ritscheri	ritscheri	ritscheri	ritscheri	ritscheri	ritscheri	obliquecostata	ritscheri	ritscheri	ritscheri	ritscheri	12	11	1	0	0	0	11	0.916	1	0	ritscheri							
http://apps3.awi.de/Hustedt-Diatoms/FragCl/Set1/L059_W8.0-S6.0-FragCl.NBP-1402.945-946cm.000012.png	59.3	7.7	6.0	NA	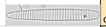	obliquecost	ritscheri	ritscheri	ritscheri	ritscheri	ritscheri		obliquecostata	ritscheri	ritscheri	ritscheri	ritscheri	11	9	2	0	0	0	9	0.818	1	0	ritscheri							
http://apps3.awi.de/Hustedt-Diatoms/FragCl/Set1/L059_W10.0-SNA-FragCl.ANT33-51.000222.png	59.4	10.1	NA	9.5	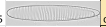	obliquecost	ritscheri	ritscheri	ritscheri	ritscheri	ritscheri		obliquecostata	ritscheri	ritscheri	ritscheri	ritscheri	11	9	2	0	0	0	9	0.818	1	0	ritscheri				marked heteropolarity			
http://apps3.awi.de/Hustedt-Diatoms/FragCl/Set1/L059_W8.0-S6.2-FragCl.NBP-1402.945-946cm.000048.png	59.4	7.6	6.2	NA	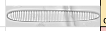	obliquecost	ritscheri	ritscheri	ritscheri	ritscheri	ritscheri		obliquecostata	ritscheri	ritscheri	ritscheri	ritscheri	11	9	2	0	0	0	9	0.818	1	0	ritscheri							
http://apps3.awi.de/Hustedt-Diatoms/FragCl/Set1/L059_W10.0-S8.7-PS1768-8.000764.png	59.4	9.8	8.7	NA	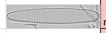	ritscheri	ritscheri	ritscheri	ritscheri	ritscheri	ritscheri		obliquecostata	ritscheri	ritscheri	ritscheri	ritscheri	11	10	1	0	0	0	10	0.909	1	0	ritscheri							
http://apps3.awi.de/Hustedt-Diatoms/FragCl/Set1/L059_W8.0-S5.5-PS1768-8.000433.png	59.5	8.1	5.5	NA	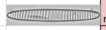	ritscheri	ritscheri	obliquecost	obliquecost	obliquecost	obliquecostata		obliquecostata	obliquecostata	obliquecostata	obliquecostata	obliquecostata	11	2	9	0	0	0	9	0.818	2	0	obliquecostata							
http://apps3.awi.de/Hustedt-Diatoms/FragCl/Set1/L059_W9.0-SNA-FragCl.NBP-1402.960-961cm.000096.png	59.5	9.0	NA	7.6	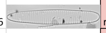	ritscheri	ritscheri	ritscheri	ritscheri	ritscheri	ritscheri	ritscheri	obliquecostata	ritscheri	ritscheri	ritscheri	ritscheri	12	11	1	0	0	0	11	0.916	1	0	ritscheri							
http://apps3.awi.de/Hustedt-Diatoms/FragCl/Set1/L060_W8.0-S7.1-FragCl.NBP-1402.945-946cm.000013.png	59.6	7.8	7.1	NA	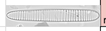	ritscheri	ritscheri	ritscheri	ritscheri	ritscheri	ritscheri	ritscheri	obliquecostata	ritscheri	ritscheri	ritscheri	ritscheri	12	11	1	0	0	0	11	0.916	1	0	ritscheri							
http://apps3.awi.de/Hustedt-Diatoms/FragCl/Set1/L060_W11.0-SNA-PS1768-8.000546.png	59.7	10.8	NA	7.8	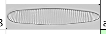	ambiguous	ritscheri	ritscheri	ritscheri	ritscheri	ritscheri	ritscheri	obliquecostata	ritscheri	ritscheri	ritscheri	ritscheri	12	10	1	0	1	0	10	0.833	1	0	ritscheri	?ritscherii/obliquecostata						
http://apps3.awi.de/Hustedt-Diatoms/FragCl/Set1/L060_W7.0-S8.0-FragCl.ANT33-51.000389.png	59.7	6.6	8.0	NA	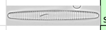	sublinearis	sublinearis	sublinearis	sublinearis	sublinearis	sublinearis	sublinearis	sublinearis	sublinearis	sublinearis	sublinearis	sublinearis	12	0	0	12	0	0	12	1	3	0	sublinearis							same specimen as 349 and 350
http://apps3.awi.de/Hustedt-Diatoms/FragCl/Set1/L060_W6.0-SNA-FragCl.ANT33-51.000391.png	59.7	6.3	NA	7.9	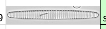	sublinearis	sublinearis	sublinearis	sublinearis	sublinearis	sublinearis	sublinearis	sublinearis	sublinearis	sublinearis	sublinearis	sublinearis	12	0	0	12	0	0	12	1	3	0	sublinearis							
http://apps3.awi.de/Hustedt-Diatoms/FragCl/Set1/L060_W5.0-S8.3-PS1768-8.000959.png	59.8	5.1	8.3	NA	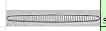	sublinearis	sublinearis	sublinearis	sublinearis	sublinearis	sublinearis	sublinearis	sublinearis	sublinearis	sublinearis	sublinearis	sublinearis	12	0	0	12	0	0	12	1	3	0	sublinearis							
http://apps3.awi.de/Hustedt-Diatoms/FragCl/Set1/L060_W6.0-SNA-FragCl.ANT33-51.000516.png	59.9	6.4	NA	7.4	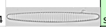	sublinearis	sublinearis	sublinearis	sublinearis	sublinearis	sublinearis	sublinearis	sublinearis	sublinearis	sublinearis	sublinearis	sublinearis	12	0	0	12	0	0	12	1	3	0	sublinearis							
http://apps3.awi.de/Hustedt-Diatoms/FragCl/Set1/L060_W6.0-S7.8-PS1768-8.000772.png	59.9	5.8	7.8	NA	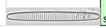	sublinearis	sublinearis	sublinearis	sublinearis	sublinearis	sublinearis	sublinearis	sublinearis	sublinearis	sublinearis	sublinearis	sublinearis	12	0	0	12	0	0	12	1	3	0	sublinearis					based purely on Transapical size (otherwise could also be obliquecostata)		

http://apps3.awi.de/Hustedt-Diatoms/FragCl/Set1/L060_W8.0-S6.3-FragCl.NBP-1402.945-946cm.000009.png	60.0	7.9	6.3	NA	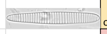	obliquecost	ritscheri	ritscheri	ritscheri	ritscheri	ritscheri	ritscheri	obliquecostata	ritscheri	ritscheri	ritscheri	ritscheri	12	10	2	0	0	0	10	0.833	1	0	ritscheri												
http://apps3.awi.de/Hustedt-Diatoms/FragCl/Set1/L060_W8.0-S6.8-FragCl.NBP-1402.960-961cm.000105.png	60.0	8.4	6.8	NA	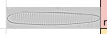	ritscheri	ritscheri	ritscheri	ritscheri	ritscheri	ritscheri	ritscheri	obliquecostata	ritscheri	ritscheri	ritscheri	ritscheri	12	11	1	0	0	0	11	0.916	1	0	ritscheri												
http://apps3.awi.de/Hustedt-Diatoms/FragCl/Set1/L060_W7.0-S5.9-FragCl.NBP-1402.945-946cm.000072.png	60.0	7.4	5.9	NA	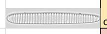	obliquecost	ritscheri	ritscheri	ritscheri	ritscheri	ritscheri	ritscheri	obliquecostata	ritscheri	ritscheri	ritscheri	ritscheri	12	10	2	0	0	0	10	0.833	1	0	ritscheri												
http://apps3.awi.de/Hustedt-Diatoms/FragCl/Set1/L060_W9.0-S7.8-FragCl.NBP-1402.999-996cm.000008.png	60.3	8.9	7.8	NA	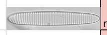	ritscheri	ritscheri	ritscheri	ritscheri	ritscheri	ritscheri	ritscheri	obliquecostata	ritscheri	ritscheri	ritscheri	ritscheri	12	11	1	0	0	0	11	0.916	1	0	ritscheri												
http://apps3.awi.de/Hustedt-Diatoms/FragCl/Set1/L060_W9.0-SNA-FragCl.NBP-1402.960-961cm.000081.png	60.3	9.2	NA	6.9	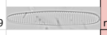	ritscheri	ritscheri	ritscheri	ritscheri	ritscheri	ritscheri		obliquecostata	ritscheri	ritscheri	ritscheri	ritscheri	11	10	1	0	0	0	10	0.909	1	0	ritscheri												
http://apps3.awi.de/Hustedt-Diatoms/FragCl/Set1/L060_W8.0-S6.4-FragCl.NBP-1402.960-961cm.000106.png	60.3	8.3	6.4	NA	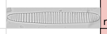	ritscheri	ritscheri	ritscheri	ritscheri	ritscheri	ritscheri	ritscheri	obliquecostata	ritscheri	ritscheri	ritscheri	ritscheri	12	11	1	0	0	0	11	0.916	1	0	ritscheri												
http://apps3.awi.de/Hustedt-Diatoms/FragCl/Set1/L060_W9.0-S6.8-FragCl.NBP-1402.960-961cm.000044.png	60.4	8.6	6.8	NA	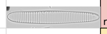	ritscheri	ritscheri	ritscheri	ritscheri	ritscheri	ritscheri	ritscheri	obliquecostata	ritscheri	ritscheri	ritscheri	ritscheri	12	11	1	0	0	0	11	0.916	1	0	ritscheri												
http://apps3.awi.de/Hustedt-Diatoms/FragCl/Set1/L060_W8.0-S7.0-FragCl.NBP-1402.945-946cm.000059.png	60.5	8.4	7.0	NA	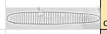	obliquecost	ritscheri	ritscheri	ritscheri	ritscheri	ritscheri	ritscheri	obliquecostata	ritscheri	ritscheri	ritscheri	ritscheri	12	10	2	0	0	0	10	0.833	1	0	ritscheri												
http://apps3.awi.de/Hustedt-Diatoms/FragCl/Set1/L060_W9.0-SNA-FragCl.NBP-1402.960-961cm.000048.png	60.5	8.9	NA	7.8	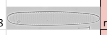	ritscheri	ritscheri	ritscheri	ritscheri	ritscheri	ritscheri		obliquecostata	ritscheri	ritscheri	ritscheri	ritscheri	11	10	1	0	0	0	10	0.909	1	0	ritscheri												
http://apps3.awi.de/Hustedt-Diatoms/FragCl/Set1/L061_W10.0-S8.3-FragCl.NBP-1402.960-961cm.000031.png	60.7	9.8	8.3	NA	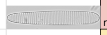	ritscheri	ritscheri	ritscheri	ritscheri	ritscheri	ritscheri		obliquecostata	ritscheri	ritscheri	ritscheri	ritscheri	11	10	1	0	0	0	10	0.909	1	0	ritscheri												
http://apps3.awi.de/Hustedt-Diatoms/FragCl/Set1/L061_W10.0-SNA-FragCl.ANT33-51.000080.png	60.7	10.4	NA	9.8	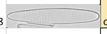	obliquecost	ritscheri	ritscheri	ritscheri	ritscheri	ritscheri		obliquecostata	ritscheri	ritscheri	ritscheri	ritscheri	11	9	2	0	0	0	9	0.818	1	0	ritscheri						could be confused with obliquecostata due to slightly oblique costae BUT heteropolarity too pointed one end (i.e. not obtuse e						
http://apps3.awi.de/Hustedt-Diatoms/FragCl/Set1/L061_W6.0-S5.1-PS1768-8.000966.png	60.8	6.3	5.1	NA	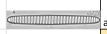	ambiguous	ambiguous	obliquecost	obliquecost	obliquecost	obliquecostata		sublinearis	obliquecostata	obliquecostata	obliquecostata	obliquecostata	11	0	8	1	2	0	8	0.727	2	0	obliquecostata	?sublinearis/kerguelensis											
http://apps3.awi.de/Hustedt-Diatoms/FragCl/Set1/L061_W8.0-S6.6-FragCl.ANT33-51.000190.png	60.8	7.9	6.6	6.5	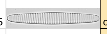	obliquecost	obliquecost	obliquecost	obliquecost	obliquecost	obliquecostata		obliquecostata	obliquecostata	obliquecostata	obliquecostata	obliquecostata	11	0	11	0	0	0	11	1	2	0	obliquecostata		sort of like F. subl	I would need more pics with other focus to be sure			could be confused with sublineata due to indiscernab			sublinearis if marginal poroids were more distinct on both sides			
http://apps3.awi.de/Hustedt-Diatoms/FragCl/Set1/L061_W10.0-S7.2-FragCl.ANT33-51.000089.png	60.8	9.9	7.2	7.2	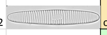	obliquecost	ritscheri	ritscheri	obliquecost	obliquecost	obliquecostata		obliquecostata	ambiguous	ritscheri	obliquecostata	ritscheri	11	4	6	0	1	0	6	0.545	2	0	obliquecostata					not heteropolar, but quite ritscheri-like				probably obliquecostata			
http://apps3.awi.de/Hustedt-Diatoms/FragCl/Set1/L061_W7.0-S6.7-PS1768-8.000768.png	61.2	7.3	6.7	NA	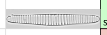	sublinearis	obliquecost	obliquecost	obliquecost	obliquecost	obliquecostata		obliquecostata	ambiguous	obliquecostata	obliquecostata	obliquecostata	11	0	9	1	1	0	9	0.818	2	0	obliquecostata		sort of like F. subl	I would need more pics with other focus to be sure						obliquecostata or sublinearis?			
http://apps3.awi.de/Hustedt-Diatoms/FragCl/Set1/L061_W8.0-S7.1-FragCl.NBP-1402.960-961cm.000080.png	61.2	8.3	7.1	NA	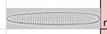	ritscheri	ritscheri	ritscheri	ritscheri	ritscheri	ritscheri		obliquecostata	ritscheri	ritscheri	ritscheri	ritscheri	11	10	1	0	0	0	10	0.909	1	0	ritscheri												
http://apps3.awi.de/Hustedt-Diatoms/FragCl/Set1/L061_W8.0-SNA-FragCl.NBP-1402.960-961cm.000076.png	61.5	8.4	NA	8.6	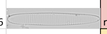	ritscheri	ritscheri	ritscheri	ritscheri	ritscheri	ritscheri		obliquecostata	ritscheri	ritscheri	ritscheri	ritscheri	11	10	1	0	0	0	10	0.909	1	0	ritscheri												
http://apps3.awi.de/Hustedt-Diatoms/FragCl/Set1/L062_W10.0-SNA-FragCl.ANT33-51.000088.png	61.5	10.4	NA	9.2	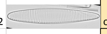	obliquecost	ritscheri	ritscheri	ritscheri	ritscheri	ritscheri		obliquecostata	ritscheri	obliquecostata	ritscheri	ritscheri	11	8	3	0	0	0	8	0.727	1	0	ritscheri				I am not sure. Atypical shape?								
http://apps3.awi.de/Hustedt-Diatoms/FragCl/Set1/L062_W8.0-S6.3-FragCl.NBP-1402.945-946cm.000069.png	61.6	8.3	6.3	NA	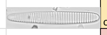	obliquecost	ritscheri	ritscheri	ritscheri	ritscheri	ritscheri		obliquecostata	ritscheri	ritscheri	ritscheri	ritscheri	11	9	2	0	0	0	9	0.818	1	0	ritscheri												
http://apps3.awi.de/Hustedt-Diatoms/FragCl/Set1/L062_W9.0-S7.0-FragCl.NBP-1402.960-961cm.000086.png	61.6	8.5	7.0	NA	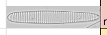	ritscheri	ritscheri	ritscheri	ritscheri	ritscheri	ritscheri	ritscheri	obliquecostata	ritscheri	ritscheri	ritscheri	ritscheri	12	11	1	0	0	0	11	0.916	1	0	ritscheri												
http://apps3.awi.de/Hustedt-Diatoms/FragCl/Set1/L062_W10.0-SNA-FragCl.ANT33-51.000184.png	61.7	10.2	NA	9.3	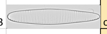	obliquecost	ritscheri	ritscheri	ritscheri	ritscheri	ritscheri		obliquecostata	ritscheri	ritscheri	ritscheri	ritscheri	11	9	2	0	0	0	9	0.818	1	0	ritscheri												
http://apps3.awi.de/Hustedt-Diatoms/FragCl/Set1/L062_W8.0-S6.0-FragCl.NBP-1402.945-946cm.000076.png	61.7	7.7	6.0	NA	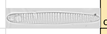	obliquecost	ritscheri	ritscheri	ambiguous	ritscheri	ritscheri		obliquecostata	ambiguous	sublinearis	ritscheri	ritscheri	11	6	2	1	2	0	6	0.545	1	0	ritscheri				ritscheri/obliquecostata		abberant form			ritscheri or obliquecostata			
http://apps3.awi.de/Hustedt-Diatoms/FragCl/Set1/L062_W9.0-S6.5-FragCl.NBP-1402.945-946cm.000044.png	61.8	8.9	6.5	NA	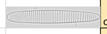	obliquecost	ritscheri	ritscheri	ritscheri	ritscheri	ritscheri		obliquecostata	ritscheri	ritscheri	ritscheri	ritscheri	11	9	2	0	0	0	9	0.818	1	0	ritscheri												
http://apps3.awi.de/Hustedt-Diatoms/FragCl/Set1/L062_W7.0-S5.9-FragCl.NBP-1402.945-946cm.000033.png	61.8	7.5	5.9	NA	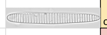	obliquecost	ritscheri	ritscheri	ritscheri	ritscheri	ritscheri		obliquecostata	ritscheri	ritscheri	ritscheri	ritscheri	11	9	2	0	0	0	9	0.818	1	0	ritscheri												
http://apps3.awi.de/Hustedt-Diatoms/FragCl/Set1/L062_W9.0-S8.0-FragCl.NBP-1402.960-961cm.000041.png	62.0	9.1	8.0	NA	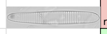	ritscheri	ritscheri	ritscheri	ritscheri	ritscheri	ritscheri	ritscheri	obliquecostata	ritscheri	ritscheri	ritscheri	ritscheri	12	11	1	0	0	0	11	0.916	1	0	ritscheri												
http://apps3.awi.de/Hustedt-Diatoms/FragCl/Set1/L062_W6.0-S8.5-FragCl.ANT33-51.000159.png	62.1	5.8	8.5	8.5	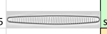	sublinearis	sublinearis	sublinearis	sublinearis	sublinearis	sublinearis		sublinearis	sublinearis	obliquecostata	sublinearis	sublinearis	11	0	1	10	0	0	10	0.909	3	0	sublinearis												
http://apps3.awi.de/Hustedt-Diatoms/FragCl/Set1/L062_W8.0-SNA-FragCl.NBP-1402.945-946cm.000030.png	62.1	8.0	NA	6.0	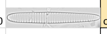	obliquecost	ritscheri	ritscheri	ritscheri	ritscheri	ritscheri		obliquecostata	ritscheri	ritscheri	ritscheri	ritscheri	11	9	2	0	0	0	9	0.818	1	0	ritscheri												
http://apps3.awi.de/Hustedt-Diatoms/FragCl/Set1/L062_W9.0-S5.6-PS1768-8.000969.png	62.1	8.5	5.6	NA	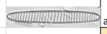	ambiguous	ambiguous	obliquecost	obliquecost	obliquecost	obliquecostata		obliquecostata	obliquecostata	obliquecostata	obliquecostata	obliquecostata	11	0	9	0	2	0	9	0.818	2	0	obliquecostata	?obliquecostata/r	obliquecostata - ritscheri										
http://apps3.awi.de/Hustedt-Diatoms/FragCl/Set1/L062_W9.0-S6.7-PS1768-8.000493.png	62.2	8.6	6.7	NA	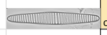	obliquecost	obliquecost	obliquecost	obliquecost	obliquecost	obliquecost	obliquecos	obliquecostata	obliquecostata	obliquecostata	obliquecostata	obliquecostata	12	0	12	0	0	0	12	1	2	0	obliquecostata												
http://apps3.awi.de/Hustedt-Diatoms/FragCl/Set1/L062_W9.0-SNA-FragCl.NBP-1402.960-961cm.000104.png	62.4	8.7	NA	7.3	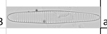	ambiguous	ritscheri	ritscheri	ritscheri	ritscheri	ritscheri	ritscheri	obliquecostata	ritscheri	ritscheri	ritscheri	ritscheri	12	10	1	0	1	0	10	0.833	1	0	ritscheri	?ritscheri/obliquecostata											
http://apps3.awi.de/Hustedt-Diatoms/FragCl/Set1/L063_W7.0-S5.1-PS1768-8.000775.png	62.6	6.6	5.1	NA	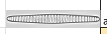	ambiguous	obliquecost	obliquecost	obliquecost	obliquecost	obliquecost	obliquecos	sublinearis	obliquecostata	obliquecostata	obliquecostata	obliquecostata	12	0	10	1	1	0	10	0.833	2	0	obliquecostata	?sublinearis/kerguelensis											
http://apps3.awi.de/Hustedt-Diatoms/FragCl/Set1/L063_W8.0-S6.6-FragCl.ANT33-76.000056.png	62.6	8.2	6.6	NA	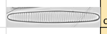	obliquecost	obliquecost	ritscheri	obliquecost	obliquecost	obliquecost	obliquecos	obliquecostata	ambiguous	obliquecostata	obliquecostata	sublinearis	12	1	9	1	1	0	9	0.75	2	0	obliquecostata									sublinearis if marginal poroids were more distinct on both sides			
http://apps3.awi.de/Hustedt-Diatoms/FragCl/Set1/L063_W9.0-S8.0-FragCl.ANT33-76.000046.png	62.8	9.1	8.0	NA	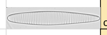	obliquecost	ritscheri	ritscheri	ritscheri	ritscheri	ritscheri	ritscheri	obliquecostata	ritscheri	ritscheri	ritscheri	ritscheri	12	10	2	0	0	0	10	0.833	1	0	ritscheri				Atypical shape?								
http://apps3.awi.de/Hustedt-Diatoms/FragCl/Set1/L063_W8.0-S6.0-PS1768-8.000468.png	62.8	8.3	6.0	NA	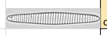	obliquecost	obliquecost	obliquecost	obliquecost	obliquecost	obliquecost	obliquecos	obliquecostata	obliquecostata	obliquecostata	obliquecostata	obliquecostata	12	0	12	0	0	0	12	1	2	0	obliquecostata												
http://apps3.awi.de/Hustedt-Diatoms/FragCl/Set1/L063_W8.0-S6.8-FragCl.ANT33-76.000050.png	62.9	7.7	6.8	NA	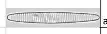	ambiguous	obliquecost	ritscheri	obliquecost	obliquecost	obliquecost	obliquecos	sublinearis	ambiguous	obliquecostata	obliquecostata	sublinearis	12	1	7	2	2	0	7	0.583	2	0	obliquecostata	?kerguelensis/obl	could also be a F. ritscheri							sublinearis if marginal poroids were more distinct on both sides			
http://apps3.awi.de/Hustedt-Diatoms/FragCl/Set1/L063_W8.0-S6.1-FragCl.NBP-1402.945-946cm.000011.png	63.1	8.5	6.1	NA	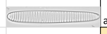	ambiguous	ritscheri	ritscheri	ritscheri	ritscheri	ritscheri	ritscheri	obliquecostata	ritscheri	ritscheri	ritscheri	ritscheri	12	10	1	0	1	0	10	0.833	1	0	ritscheri	?ritscheri/obliquecostata											
http://apps3.awi.de/Hustedt-Diatoms/FragCl/Set1/L063_W10.0-S9.0-FragCl.ANT33-51.000008.png	63.3	10.3	9.0	9.2	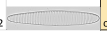	obliquecost	ritscheri	ritscheri	ritscheri	ritscheri	ritscheri	ritscheri	obliquecostata	ritscheri	ritscheri	ritscheri	ritscheri	12	10	2	0	0	0	10	0.833	1	0	ritscheri						could be confused with obliquecostata but more heteropolar enough although costae oblique and central region slightly inflate						

http://apps3.awi.de/Hustedt-Diatoms/FragCl/Set1/L063_W9.0-S6.4-FragCl.NBP-1402.960-961cm.000036.png	63.3	8.5	6.4	NA	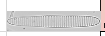	ritscheri	ritscheri	ritscheri	ritscheri	ritscheri	ritscheri	ritscheri	obliquecostata	ritscheri	ritscheri	ritscheri	ritscheri	12	11	1	0	0	0	11	0.916	1	0	ritscheri												
http://apps3.awi.de/Hustedt-Diatoms/FragCl/Set1/L063_W8.0-S5.6-PS1768-8.000977.png	63.4	8.1	5.6	NA	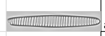	ambiguous	obliquecost	obliquecost	obliquecost	obliquecost	obliquecost	obliquecos	obliquecostata	obliquecostata	obliquecostata	obliquecostata	obliquecostata	12	0	11	0	1	0	11	0.916	2	0	obliquecostata	?obliquecostata/kerguelensis											
http://apps3.awi.de/Hustedt-Diatoms/FragCl/Set1/L063_W7.0-S6.1-PS1768-8.000514.png	63.4	7.5	6.1	NA	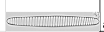	ambiguous	obliquecost	obliquecost	obliquecost	obliquecost	obliquecost	obliquecos	sublinearis	obliquecostata	obliquecostata	obliquecostata	obliquecostata	12	0	10	1	1	0	10	0.833	2	0	obliquecostata	?sublinearis/obliquecostata											
http://apps3.awi.de/Hustedt-Diatoms/FragCl/Set1/L063_W11.0-S8.1-PS1768-8.000970.png	63.4	11.0	8.1	NA	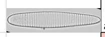	ambiguous	ritscheri	ritscheri	ritscheri	ritscheri	ritscheri	ritscheri	obliquecostata	ritscheri	ritscheri	ritscheri	ritscheri	12	10	1	0	1	0	10	0.833	1	0	ritscheri	?ritscherii/obliquecostata											
http://apps3.awi.de/Hustedt-Diatoms/FragCl/Set1/L063_W8.0-S7.0-FragCl.NBP-1402.999-996cm.000007.png	63.4	8.4	7.0	NA	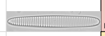	ritscheri	ritscheri	ritscheri	ritscheri	ritscheri	ritscheri	ritscheri	obliquecostata	ritscheri	ritscheri	ritscheri	ritscheri	12	11	1	0	0	0	11	0.916	1	0	ritscheri												
http://apps3.awi.de/Hustedt-Diatoms/FragCl/Set1/L063_W8.0-S6.1-FragCl.NBP-1402.945-946cm.000026.png	63.5	8.0	6.1	NA	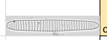	obliquecost	ambiguous	ritscheri	ritscheri	ritscheri	ritscheri	ritscheri	obliquecostata	ritscheri	sublinearis	ritscheri	ritscheri	12	8	2	1	1	0	8	0.666	1	0	ritscheri		obliquecostata - ritscheri										
http://apps3.awi.de/Hustedt-Diatoms/FragCl/Set1/L064_W8.0-S7.2-FragCl.NBP-1402.999-996cm.000019.png	63.6	7.8	7.2	NA	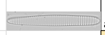	ambiguous	ritscheri	ritscheri	ritscheri	ritscheri	ritscheri		obliquecostata	ritscheri	ritscheri	ritscheri	ritscheri	11	9	1	0	1	0	9	0.818	1	0	ritscheri	?ritscheri/obliquecostata											
http://apps3.awi.de/Hustedt-Diatoms/FragCl/Set1/L064_W9.0-SNA-FragCl.ANT33-51.000221.png	63.6	8.7	NA	7.1	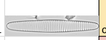	obliquecost	obliquecost	ritscheri	obliquecost	obliquecost	obliquecostata		obliquecostata	obliquecostata	obliquecostata	obliquecostata	ritscheri	11	2	9	0	0	0	9	0.818	2	0	obliquecostata		gradational to F. ritscheri							sublinearis if marginal poroids were more distinct on both sides			
http://apps3.awi.de/Hustedt-Diatoms/FragCl/Set1/L064_W9.0-S7.2-FragCl.NBP-1402.999-996cm.000013.png	63.7	8.8	7.2	NA	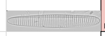	ritscheri	ritscheri	ritscheri	ritscheri	ritscheri	ritscheri		obliquecostata	ritscheri	obliquecostata	ritscheri	ritscheri	11	9	2	0	0	0	9	0.818	1	0	ritscheri		gradational to F. obliquecostata										
http://apps3.awi.de/Hustedt-Diatoms/FragCl/Set1/L064_W11.0-S8.4-PS1768-8.000012.png	63.7	11.3	8.4	NA	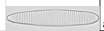	ambiguous	ritscheri	ritscheri	ritscheri	ritscheri	ritscheri	ritscheri	obliquecostata	ritscheri	ritscheri	ritscheri	ritscheri	12	10	1	0	1	0	10	0.833	1	0	ritscheri	?ritscherii/obliquecostata											
http://apps3.awi.de/Hustedt-Diatoms/FragCl/Set1/L064_W7.0-S6.7-PS1768-8.000769.png	63.7	6.9	6.7	NA	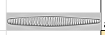	ambiguous	obliquecost	ritscheri	ambiguous	obliquecost	obliquecost	obliquecos	sublinearis	ambiguous	obliquecostata	obliquecostata	ritscheri	12	2	6	1	3	0	6	0.5	2	0	obliquecostata	?sublinearis/kerguelensis			ritscheri/obliquec	although striae ar	image is oblique (	not 100% sure (object angle on pho		probably obliquecostata			
http://apps3.awi.de/Hustedt-Diatoms/FragCl/Set1/L064_W8.0-S6.7-FragCl.ANT33-51.000191.png	63.8	8.1	6.7	6.4	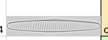	obliquecost	obliquecost	ritscheri	obliquecost	obliquecost	obliquecost	obliquecos	obliquecostata	obliquecostata	obliquecostata	obliquecostata	ritscheri	12	2	10	0	0	0	10	0.833	2	0	obliquecostata									sublinearis if marginal poroids were more distinct on both sides			
http://apps3.awi.de/Hustedt-Diatoms/FragCl/Set1/L064_W6.0-SNA-FragCl.ANT33-51.000519.png	64.0	5.8	NA	9.1		sublinearis	sublinearis	sublinearis	sublinearis	sublinearis	sublinearis	sublinearis	sublinearis	sublinearis	sublinearis	sublinearis	sublinearis	12	0	0	12	0	0	12	1	3	0	sublinearis					although could be	could be confused	based purely on Transapical size (ot		possibly obliquecostata			
http://apps3.awi.de/Hustedt-Diatoms/FragCl/Set1/L064_W9.0-S6.9-FragCl.NBP-1402.960-961cm.000088.png	64.3	8.9	6.9	NA	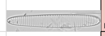	ritscheri	ritscheri	ritscheri	ritscheri	ritscheri	ritscheri		obliquecostata	ritscheri	ritscheri	ritscheri	ritscheri	11	10	1	0	0	0	10	0.909	1	0	ritscheri												
http://apps3.awi.de/Hustedt-Diatoms/FragCl/Set1/L065_W10.0-S7.0-PS1768-8.000847.png	64.8	9.5	7.0	NA	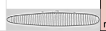	ritscheri	ritscheri	ritscheri	ritscheri	ritscheri	ritscheri		obliquecostata	ritscheri	ritscheri	ritscheri	ritscheri	11	10	1	0	0	0	10	0.909	1	0	ritscheri						could be confused with obliquecostata due to slightly oblique costae BUT heteropolarity too pointed one end						
http://apps3.awi.de/Hustedt-Diatoms/FragCl/Set1/L065_W8.0-S6.9-FragCl.NBP-1402.960-961cm.000085.png	64.8	8.2	6.9	NA	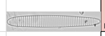	ritscheri	ritscheri	ritscheri	ritscheri	ritscheri	ritscheri		obliquecostata	ritscheri	obliquecostata	ritscheri	ritscheri	11	9	2	0	0	0	9	0.818	1	0	ritscheri												
http://apps3.awi.de/Hustedt-Diatoms/FragCl/Set1/L065_W10.0-S7.0-PS1768-8.000974.png	64.8	9.8	7.0	NA	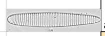	ambiguous	ritscheri	ritscheri	ritscheri	ritscheri	ritscheri		obliquecostata	ritscheri	ritscheri	ritscheri	ritscheri	11	9	1	0	1	0	9	0.818	1	0	ritscheri	?ritscherii/obliquecostata											
http://apps3.awi.de/Hustedt-Diatoms/FragCl/Set1/L065_W11.0-SNA-FragCl.ANT33-51.000095.png	64.8	10.7	NA	9.7	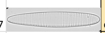	obliquecost	ritscheri	ritscheri	ritscheri	ritscheri	ritscheri		obliquecostata	ritscheri	sublinearis	ritscheri	ritscheri	11	8	2	1	0	0	8	0.727	1	0	ritscheri				Atypical shape?								
http://apps3.awi.de/Hustedt-Diatoms/FragCl/Set1/L065_W9.0-S7.2-PS1768-8.000464.png	65.1	9.0	7.2	NA	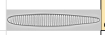	obliquecost	ritscheri	ritscheri	ritscheri	ritscheri	ritscheri		obliquecostata	ritscheri	ritscheri	ritscheri	ritscheri	11	9	2	0	0	0	9	0.818	1	0	ritscheri					heteropolar							
http://apps3.awi.de/Hustedt-Diatoms/FragCl/Set1/L065_W9.0-S6.4-PS1768-8.000850.png	65.3	8.5	6.4	NA	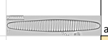	ambiguous	obliquecost	ritscheri	obliquecost	obliquecost	obliquecostata		obliquecostata	ambiguous	obliquecostata	obliquecostata	ritscheri	11	2	7	0	2	0	7	0.636	2	0	obliquecostata	?kerguelensis/obliquecostata/ritscherii				although striae are not very slanted, shape is ok				sublinearis or obliquecostata depending on marginal poroids			
http://apps3.awi.de/Hustedt-Diatoms/FragCl/Set1/L065_W9.0-S7.8-FragCl.ANT33-51.000388.png	65.4	9.2	7.8	NA	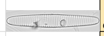	obliquecost	obliquecost	ritscheri	obliquecost	obliquecost	obliquecostata		obliquecostata	obliquecostata	obliquecostata	obliquecostata	ritscheri	11	2	9	0	0	0	9	0.818	2	0	obliquecostata		gradational to F. ritscheri							sublinearis if marginal poroids were more distinct on both sides			
http://apps3.awi.de/Hustedt-Diatoms/FragCl/Set1/L066_W8.0-S6.3-FragCl.NBP-1402.945-946cm.000046.png	65.5	7.6	6.3	NA	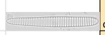	obliquecost	ritscheri	ritscheri	ritscheri	ritscheri	ritscheri		obliquecostata	ritscheri	ritscheri	ritscheri	ritscheri	11	9	2	0	0	0	9	0.818	1	0	ritscheri					striae striaght so not obliquecostata, but not typical ritscheri. would say sublinearis if ends were same shape							
http://apps3.awi.de/Hustedt-Diatoms/FragCl/Set1/L066_W9.0-SNA-FragCl.ANT33-100.000082.png	65.6	8.9	NA	9.6	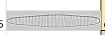	obliquecost	obliquecost	ritscheri	ritscheri	ritscheri	ritscheri	obliquecos	obliquecostata	ambiguous	obliquecostata	obliquecostata	ritscheri	12	5	6	0	1	0	6	0.5	2	0	obliquecostata				Atypical shape?	has obliquecostat	could be confused with obliquecostata due to inflated			?			
http://apps3.awi.de/Hustedt-Diatoms/FragCl/Set1/L066_W9.0-S7.9-FragCl.NBP-1402.999-996cm.000014.png	65.8	8.5	7.9	NA	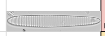	ritscheri	ritscheri	ritscheri	ritscheri	ritscheri	ritscheri	ritscheri	obliquecostata	ritscheri	ritscheri	obliquecostata	ritscheri	12	10	2	0	0	0	10	0.833	1	0	ritscheri												
http://apps3.awi.de/Hustedt-Diatoms/FragCl/Set1/L066_W8.0-S7.2-FragCl.ANT33-51.000197.png	66.0	8.4	7.2	NA	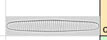	obliquecost	obliquecost	ritscheri	obliquecost	obliquecost	obliquecost	obliquecos	obliquecostata	ambiguous	obliquecostata	obliquecostata	ritscheri	12	2	9	0	1	0	9	0.75	2	0	obliquecostata									sublinearis or obliquecostata depending on marginal poroids			
http://apps3.awi.de/Hustedt-Diatoms/FragCl/Set1/L066_W6.0-S8.8-FragCl.ANT33-51.000524.png	66.2	6.3	8.8	NA		sublinearis	sublinearis	sublinearis	sublinearis	sublinearis	sublinearis		sublinearis	sublinearis	obliquecostata	sublinearis	sublinearis	11	0	1	10	0	0	10	0.909	3	0	sublinearis						could be confused with obliquecostata due to inflated central section and cosata oblique towards poles but has indiscernable p						
http://apps3.awi.de/Hustedt-Diatoms/FragCl/Set1/L066_W8.0-S6.2-PS1768-8.000427.png	66.2	8.4	6.2	NA	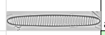	ambiguous	obliquecost	obliquecost	obliquecost	obliquecost	obliquecostata		obliquecostata	obliquecostata	obliquecostata	obliquecostata	obliquecostata	11	0	10	0	1	0	10	0.909	2	0	obliquecostata	?ritschrii/obliquecostata											
http://apps3.awi.de/Hustedt-Diatoms/FragCl/Set1/L066_W6.0-S8.0-FragCl.ANT33-51.000515.png	66.3	6.4	8.0	NA		sublinearis	sublinearis	sublinearis	sublinearis	sublinearis	sublinearis		sublinearis	sublinearis	sublinearis	sublinearis	sublinearis	11	0	0	11	0	0	11	1	3	0	sublinearis												
http://apps3.awi.de/Hustedt-Diatoms/FragCl/Set1/L066_W8.0-S5.9-FragCl.NBP-1402.945-946cm.000028.png	66.4	8.1	5.9	NA	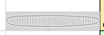	obliquecost	ritscheri	ritscheri	ritscheri	ritscheri	ritscheri		obliquecostata	ritscheri	obliquecostata	ritscheri	ritscheri	11	8	3	0	0	0	8	0.727	1	0	ritscheri												
http://apps3.awi.de/Hustedt-Diatoms/FragCl/Set1/L066_W6.0-SNA-FragCl.ANT33-51.000099.png	66.4	6.1	NA	7.8		sublinearis	sublinearis	sublinearis	sublinearis	sublinearis	sublinearis		sublinearis	ambiguous	sublinearis	sublinearis	sublinearis	11	0	0	10	1	0	10	0.909	3	0	sublinearis									sublinearis if marginal poroids were more distinct on both sides			
http://apps3.awi.de/Hustedt-Diatoms/FragCl/Set1/L066_W9.0-S7.4-FragCl.NBP-1402.999-996cm.000018.png	66.5	9.0	7.4	NA	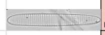	ritscheri	ritscheri	ritscheri	ritscheri	ritscheri	ritscheri	ritscheri	obliquecostata	ritscheri	ritscheri	ritscheri	ritscheri	12	11	1	0	0	0	11	0.916	1	0	ritscheri												
http://apps3.awi.de/Hustedt-Diatoms/FragCl/Set1/L067_W9.0-S7.7-FragCl.NBP-1402.960-961cm.000092.png	66.7	8.9	7.7	NA	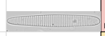	ritscheri	ritscheri	ritscheri	ritscheri	ritscheri	ritscheri	ritscheri	obliquecostata	ritscheri	obliquecostata	obliquecostata	ritscheri	12	9	3	0	0	0	9	0.75	1	0	ritscheri						could be confused with obliquecostata due to slightly oblique costae BUT heteropolarity too pointed one end (i.e. not obtuse e						
http://apps3.awi.de/Hustedt-Diatoms/FragCl/Set1/L067_W8.0-S6.1-FragCl.NBP-1402.945-946cm.000017.png	67.0	8.0	6.1	NA	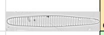	obliquecost	ritscheri	ritscheri	ritscheri	ritscheri	ritscheri	ritscheri	obliquecostata	ritscheri	obliquecostata	obliquecostata	ritscheri	12	8	4	0	0	0	8	0.666	1	0	ritscheri												
http://apps3.awi.de/Hustedt-Diatoms/FragCl/Set1/L067_W6.0-S8.7-PS1768-8.000960.png	67.2	5.9	8.7	NA	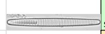	sublinearis	sublinearis	sublinearis	sublinearis	sublinearis	obliquecost	sublinearis	sublinearis	sublinearis	obliquecostata	sublinearis	sublinearis	12	0	2	10	0	0	10	0.833	3	0	sublinearis							based purely on Transapical size (otherwise could also be obliquecostata)					
http://apps3.awi.de/Hustedt-Diatoms/FragCl/Set1/L067_W9.0-S6.0-FragCl.NBP-1402.945-946cm.000025.png	67.2	8.9	6.0	NA	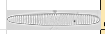	obliquecost	ritscheri	ritscheri	ambiguous	ritscheri	ritscheri	ritscheri	obliquecostata	ritscheri	obliquecostata	ritscheri	ritscheri	12	8	3	0	1	0	8	0.666	1	0	ritscheri				ritscheri/obliquecostata								
http://apps3.awi.de/Hustedt-Diatoms/FragCl/Set1/L067_W10.0-S7.5-FragCl.ANT33-51.000137.png	67.3	9.7	7.5	6.9	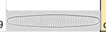	obliquecost	obliquecost	ritscheri	obliquecost	obliquecost	obliquecost	obliquecos	obliquecostata	ambiguous	obliquecostata	obliquecostata	ritscheri	12	2	9	0	1	0	9	0.75	2	0	obliquecostata									sublinearis or obliquecostata depending on marginal poroids			
http://apps3.awi.de/Hustedt-Diatoms/FragCl/Set1/L067_W7.0-S5.4-PS1768-8.000771.png	67.4	7.2	5.4	NA	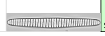	sublinearis	obliquecost	obliquecost	obliquecost	obliquecost	obliquecost	obliquecos	obliquecostata	obliquecostata	obliquecostata	obliquecostata	obliquecostata	12	0	11	1	0	0	11	0.916	2	0	obliquecostata												

http://apps3.awi.de/Hustedt-Diatoms/FragCl/Set1/L067_W9.0-S7.3-FragCl.ANT33-51.000169.png	67.4	9.2	7.3	8.3	obliquecost	ambiguous	ritscheri	obliquecost	ritscheri	obliquecost	obliquecos	obliquecostata	ambiguous	obliquecostata	obliquecostata	ritscheri	12	3	7	0	2	0	7	0.583	2	0	obliquecostata		gradational F. obliquecostata - F. ritscheri										
http://apps3.awi.de/Hustedt-Diatoms/FragCl/Set1/L067_W8.0-S5.6-FragCl.NBP-1402.945-946cm.000055.png	67.4	8.2	5.6	NA	obliquecost	ritscheri	ritscheri	ritscheri	ritscheri	ritscheri	ritscheri	obliquecostata	ritscheri	ritscheri	ritscheri	ritscheri	12	10	2	0	0	0	10	0.833	1	0	ritscheri												
http://apps3.awi.de/Hustedt-Diatoms/FragCl/Set1/L068_W7.0-S6.0-PS1768-8.000425.png	67.7	6.9	6.0	NA	sublinearis	ambiguous	obliquecost	ambiguous	sublinearis	sublinearis	obliquecos	sublinearis	ambiguous	sublinearis	sublinearis	sublinearis	12	0	2	7	3	0	7	0.583	3	0	sublinearis		striae not oblique	seems isopolar	obliquecostata/sublinearis			based purely on Transapical size (ot		sublinearis or obliquecostata depending on marginal poroids			
http://apps3.awi.de/Hustedt-Diatoms/FragCl/Set1/L068_W8.0-S6.4-FragCl.NBP-1402.960-961cm.000040.png	67.7	8.2	6.4	NA	ritscheri	ritscheri	ritscheri	ritscheri	ritscheri	ritscheri	ritscheri	obliquecostata	ritscheri	obliquecostata	ritscheri	ritscheri	12	10	2	0	0	0	10	0.833	1	0	ritscheri												
http://apps3.awi.de/Hustedt-Diatoms/FragCl/Set1/L068_W10.0-SNA-FragCl.ANT33-76.000042.png	67.7	9.9	NA	10.4	ambiguous	ritscheri	ritscheri	ritscheri	ritscheri	ritscheri	ritscheri	obliquecostata	ritscheri	obliquecostata	ritscheri	ritscheri	12	9	2	0	1	0	9	0.75	1	0	ritscheri	?ritscherii/obliquecostata											
http://apps3.awi.de/Hustedt-Diatoms/FragCl/Set1/L068_W8.0-S4.8-PS1768-8.000776.png	67.7	7.5	4.8	NA	ambiguous	obliquecost	obliquecost	obliquecost	obliquecost	obliquecost	obliquecos	sublinearis	obliquecostata	obliquecostata	obliquecostata	obliquecostata	12	0	10	1	1	0	10	0.833	2	0	obliquecostata	?sublinearis/kerguelensis											
http://apps3.awi.de/Hustedt-Diatoms/FragCl/Set1/L068_W7.0-SNA-PS1768-8.000501.png	67.9	7.2	NA	4.9	ambiguous	obliquecost	obliquecost	obliquecost	obliquecost	sublinearis	obliquecos	sublinearis	obliquecostata	obliquecostata	obliquecostata	obliquecostata	12	0	9	2	1	0	9	0.75	2	0	obliquecostata	?sublinearis/obliquecostata											
http://apps3.awi.de/Hustedt-Diatoms/FragCl/Set1/L068_W9.0-S7.7-FragCl.ANT33-76.000043.png	67.9	8.7	7.7	NA	ambiguous	obliquecost	ritscheri	obliquecost	sublinearis	obliquecostata		obliquecostata	ambiguous	obliquecostata	obliquecostata	ritscheri	11	2	6	1	2	0	6	0.545	2	0	obliquecostata	?ritscherii/obliquecostata					could be confused with ritscherii, but not heteropolar			sublinearis or obliquecostata depending on marginal poroids			
http://apps3.awi.de/Hustedt-Diatoms/FragCl/Set1/L068_W8.0-S6.7-FragCl.NBP-1402.999-996cm.000017.png	68.0	8.0	6.7	NA	ambiguous	ritscheri	ritscheri	ritscheri	ritscheri	ritscheri		obliquecostata	ritscheri	obliquecostata	ritscheri	ritscheri	11	8	2	0	1	0	8	0.727	1	0	ritscheri	?ritscheri/obliquecostata				heteropolar, although some hint of slanted striae							
http://apps3.awi.de/Hustedt-Diatoms/FragCl/Set1/L068_W7.0-S8.7-FragCl.ANT33-51.000125.png	68.0	6.5	8.7	8.7	sublinearis	sublinearis	sublinearis	sublinearis	sublinearis	sublinearis	sublinearis	sublinearis	sublinearis	obliquecostata	sublinearis	sublinearis	12	0	1	11	0	0	11	0.916	3	0	sublinearis			keel punctae present?				based purely on Transapical size (otherwise could also be obliquecostata)					
http://apps3.awi.de/Hustedt-Diatoms/FragCl/Set1/L068_W8.0-S6.7-FragCl.NBP-1402.960-961cm.000097.png	68.4	8.1	6.7	NA	ritscheri	ritscheri	ritscheri	ritscheri	ritscheri	ritscheri	ritscheri	obliquecostata	ritscheri	ritscheri	ritscheri	ritscheri	12	11	1	0	0	0	11	0.916	1	0	ritscheri												
http://apps3.awi.de/Hustedt-Diatoms/FragCl/Set1/L069_W10.0-S6.7-PS1768-8.000495.png	68.7	10.0	6.7	NA	obliquecost	ritscheri	ritscheri	ritscheri	ritscheri	ritscheri		obliquecostata	ritscheri	obliquecostata	obliquecostata	ritscheri	11	7	4	0	0	0	7	0.636	1	0	ritscheri		gradational to F. obliquecostata										
http://apps3.awi.de/Hustedt-Diatoms/FragCl/Set1/L069_W8.0-S7.7-FragCl.NBP-1402.960-961cm.000090.png	68.8	8.2	7.7	NA	ritscheri	ritscheri	ritscheri	ritscheri	ritscheri	ritscheri		obliquecostata	ritscheri	obliquecostata	ritscheri	ritscheri	11	9	2	0	0	0	9	0.818	1	0	ritscheri		gradational to F. obliquecostata				could be confused with obliquecostata due to slightly oblique costae BUT heteropolarity too pointed one end						
http://apps3.awi.de/Hustedt-Diatoms/FragCl/Set1/L069_W8.0-S6.0-FragCl.NBP-1402.945-946cm.000031.png	68.8	8.1	6.0	NA	obliquecost	ritscheri	ritscheri	ritscheri	ritscheri	ritscheri		obliquecostata	ritscheri	obliquecostata	obliquecostata	obliquecostata	11	6	5	0	0	0	6	0.545	1	0	ritscheri												
http://apps3.awi.de/Hustedt-Diatoms/FragCl/Set1/L069_W7.0-S5.7-PS1768-8.000544.png	69.1	7.2	5.7	NA	ambiguous	obliquecost	obliquecost	obliquecost	obliquecost	obliquecostata		peragallii	obliquecostata	obliquecostata	sublinearis	obliquecostata	11	0	8	1	1	1	8	0.727	2	0	obliquecostata	?sublinearis/obliquecostata											
http://apps3.awi.de/Hustedt-Diatoms/FragCl/Set1/L069_W7.0-S6.0-PS1768-8.000480.png	69.1	7.2	6.0	NA	ambiguous	obliquecost	obliquecost	obliquecost	obliquecost	obliquecostata		sublinearis	obliquecostata	obliquecostata	sublinearis	obliquecostata	11	0	8	2	1	0	8	0.727	2	0	obliquecostata	?sublinearis/obliquecostata											
http://apps3.awi.de/Hustedt-Diatoms/FragCl/Set1/L069_W9.0-S5.7-FragCl.ANT33-100.000107.png	69.4	9.0	5.7	6.0	ambiguous	obliquecost	obliquecost	obliquecost	obliquecost	sublinearis		obliquecostata	ambiguous	obliquecostata	obliquecostata	obliquecostata	11	0	8	1	2	0	8	0.727	2	0	obliquecostata	?kerguelensis/obliquecostata											seems isopolar
http://apps3.awi.de/Hustedt-Diatoms/FragCl/Set1/L069_W7.0-S5.7-PS1768-8.000858.png	69.4	7.1	5.7	NA	ambiguous	obliquecost	obliquecost	obliquecost	obliquecost	obliquecostata		sublinearis	obliquecostata	obliquecostata	sublinearis	obliquecostata	11	0	8	2	1	0	8	0.727	2	0	obliquecostata	?sublinearis/obliquecostata											
http://apps3.awi.de/Hustedt-Diatoms/FragCl/Set1/L069_W10.0-S7.6-PS1768-8.000466.png	69.4	10.1	7.6	NA	obliquecost	ritscheri	ritscheri	ritscheri	ritscheri	ritscheri		obliquecostata	ritscheri	ritscheri	obliquecostata	ritscheri	11	8	3	0	0	0	8	0.727	1	0	ritscheri												
http://apps3.awi.de/Hustedt-Diatoms/FragCl/Set1/L069_W9.0-S6.4-FragCl.NBP-1402.960-961cm.000108.png	69.5	8.6	6.4	NA	obliquecost	ritscheri	ritscheri	ritscheri	ritscheri	ritscheri		obliquecostata	ritscheri	obliquecostata	obliquecostata	ritscheri	11	7	4	0	0	0	7	0.636	1	0	ritscheri					heteropolar	could be confused with obliquecostata due to slightly oblique costae BUT heteropolarity too pointed one end						
http://apps3.awi.de/Hustedt-Diatoms/FragCl/Set1/L070_W8.0-S8.0-PS1768-8.000973.png	69.5	8.0	8.0	NA	ambiguous	obliquecost	obliquecost	obliquecost	obliquecost	obliquecostata		obliquecostata	ambiguous	sublinearis	obliquecostata	obliquecostata	11	0	8	1	2	0	8	0.727	2	0	obliquecostata	?ritscherii/obliqu	gradational to F. ritscheri			really not sure about this; not heteropolar, some slanted striae				sublinearis or obliquecostata depending on marginal poroids			
http://apps3.awi.de/Hustedt-Diatoms/FragCl/Set1/L070_W5.0-SNA-FragCl.NBP-1402.960-961cm.000084.png	69.9	5.3	NA	9.0	sublinearis	sublinearis	sublinearis	sublinearis	sublinearis	sublinearis		vanheurckii	sublinearis	obliquecostata	sublinearis	sublinearis	11	0	1	9	0	1	9	0.818	3	0	sublinearis					could be vanheurckii							
http://apps3.awi.de/Hustedt-Diatoms/FragCl/Set1/L070_W8.0-S6.8-PS1768-8.000441.png	70.2	7.5	6.8	NA	ambiguous	obliquecost	obliquecost	ambiguous	sublinearis	obliquecostata		obliquecostata	ambiguous	sublinearis	sublinearis	obliquecostata	11	0	5	3	3	0	5	0.454	2	0	obliquecostata	?sublinearis/obliq	gradational to F. ritscheri		obliquecostata/sublinearis		could be sublinearis			sublinearis or obliquecostata depending on marginal poroids			
http://apps3.awi.de/Hustedt-Diatoms/FragCl/Set1/L070_W8.0-SNA-FragCl.NBP-1402.945-946cm.000053.png	70.4	8.0	NA	6.4	obliquecost	ritscheri	ritscheri	ritscheri	ritscheri	ritscheri		obliquecostata	ritscheri	obliquecostata	obliquecostata	ritscheri	11	7	4	0	0	0	7	0.636	1	0	ritscheri												
http://apps3.awi.de/Hustedt-Diatoms/FragCl/Set1/L071_W8.0-S6.1-FragCl.NBP-1402.945-946cm.000057.png	70.8	8.2	6.1	NA	obliquecost	ritscheri	ritscheri	ambiguous	ritscheri	ritscheri		obliquecostata	ritscheri	obliquecostata	obliquecostata	ritscheri	11	6	4	0	1	0	6	0.545	1	0	ritscheri		oblique striae - can see both valves		obliquecostata/sublinearis (out of focus)								
http://apps3.awi.de/Hustedt-Diatoms/FragCl/Set1/L071_W6.0-S6.1-PS1768-8.000423.png	70.9	6.4	6.1	NA	sublinearis	sublinearis	obliquecost	ambiguous	sublinearis	sublinearis		sublinearis	sublinearis	sublinearis	sublinearis	obliquecostata	11	0	2	8	1	0	8	0.727	3	0	sublinearis				obliquecostata/sublinearis								
http://apps3.awi.de/Hustedt-Diatoms/FragCl/Set1/L071_W8.0-S7.2-PS1768-8.000415.png	71.0	7.7	7.2	NA	ambiguous	obliquecost	obliquecost	obliquecost	sublinearis	obliquecost	obliquecos	sublinearis	sublinearis	obliquecostata	sublinearis	obliquecostata	12	0	7	4	1	0	7	0.583	2	0	obliquecostata	?sublinearis/obliquecostata											
http://apps3.awi.de/Hustedt-Diatoms/FragCl/Set1/L071_W8.0-S6.5-FragCl.NBP-1402.960-961cm.000071.png	71.2	7.9	6.5	NA	obliquecost	ritscheri	ritscheri	ritscheri	ritscheri	ritscheri		obliquecostata	ritscheri	obliquecostata	ritscheri	ritscheri	11	8	3	0	0	0	8	0.727	1	0	ritscheri												
http://apps3.awi.de/Hustedt-Diatoms/FragCl/Set1/L071_W8.0-S8.0-PS1768-8.000439.png	71.4	7.8	8.0	NA	ambiguous	obliquecost	obliquecost	obliquecost	sublinearis	obliquecost	obliquecos	obliquecostata	ambiguous	obliquecostata	obliquecostata	obliquecostata	12	0	9	1	2	0	9	0.75	2	0	obliquecostata	?sublinearis/obliquecostata								sublinearis or obliquecostata depending on marginal poroids			
http://apps3.awi.de/Hustedt-Diatoms/FragCl/Set1/L072_W8.0-S6.5-PS1768-8.000524.png	71.9	8.0	6.5	NA	obliquecost	obliquecost	obliquecost	obliquecost	obliquecost	obliquecost	obliquecos	obliquecostata	obliquecostata	obliquecostata	obliquecostata	obliquecostata	12	0	12	0	0	0	12	1	2	0	obliquecostata									sublinearis or obliquecostata depending on marginal poroids			
http://apps3.awi.de/Hustedt-Diatoms/FragCl/Set1/L072_W9.0-S7.8-FragCl.ANT33-76.000052.png	72.1	9.3	7.8	NA	obliquecost	ritscheri	ritscheri	ritscheri	ritscheri	ritscheri	ritscheri	obliquecostata	ritscheri	obliquecostata	obliquecostata	ritscheri	12	8	4	0	0	0	8	0.666	1	0	ritscheri		gradational to F. obliquecostata		Atypical shape?		could be confused with obliquecostata due to slightly oblique costae BUT heteropolarity too pointed one end (i.e. not obtuse e						
http://apps3.awi.de/Hustedt-Diatoms/FragCl/Set1/L072_W9.0-S6.5-FragCl.NBP-1402.960-961cm.000100.png	72.3	8.8	6.5	NA	ambiguous	ritscheri	ritscheri	ritscheri	ritscheri	ritscheri	ritscheri	obliquecostata	ritscheri	obliquecostata	obliquecostata	ritscheri	12	8	3	0	1	0	8	0.666	1	0	ritscheri	?ritscheri/obliquecostata											
http://apps3.awi.de/Hustedt-Diatoms/FragCl/Set1/L073_W8.0-S5.3-PS1768-8.000861.png	72.6	7.7	5.3	NA	obliquecost	obliquecost	obliquecost	obliquecost	obliquecost	obliquecost	obliquecos	sublinearis	obliquecostata	obliquecostata	obliquecostata	obliquecostata	12	0	11	1	0	0	11	0.916	2	0	obliquecostata												
http://apps3.awi.de/Hustedt-Diatoms/FragCl/Set1/L073_W8.0-S6.2-FragCl.NBP-1402.960-961cm.000098.png	72.6	8.0	6.2	NA	ambiguous	ritscheri	ritscheri	ritscheri	ritscheri	ritscheri	ritscheri	obliquecostata	ritscheri	ritscheri	obliquecostata	ritscheri	12	9	2	0	1	0	9	0.75	1	0	ritscheri	?ritscheri/obliquecostata											
http://apps3.awi.de/Hustedt-Diatoms/FragCl/Set1/L073_W10.0-SNA-FragCl.ANT33-51.000126.png	72.7	10.0	NA	9.2	obliquecost	ritscheri	ritscheri	ritscheri	ritscheri	obliquecost	ritscheri	obliquecostata	ritscheri	obliquecostata	obliquecostata	ritscheri	12	7	5	0	0	0	7	0.583	1	0	ritscheri		gradational to F. obliquecostata		Atypical shape?		could be confused with ritscheri due to strong hetropolarity, but costae oblique, and width over-inflated albeit not in the centr						
http://apps3.awi.de/Hustedt-Diatoms/FragCl/Set1/L073_W7.0-S6.9-PS1768-8.000845.png	73.4	6.7	6.9	NA	ambiguous	obliquecost	obliquecost	obliquecost	obliquecost	obliquecost	obliquecos	sublinearis	obliquecostata	obliquecostata	obliquecostata	obliquecostata	12	0	10	1	1	0	10	0.833	2	0	obliquecostata	?sublinearis/obliquecostata											
http://apps3.awi.de/Hustedt-Diatoms/FragCl/Set1/L073_W8.0-S5.7-PS1768-8.000857.png	73.4	7.5	5.7	NA	obliquecost	obliquecost	obliquecost	obliquecost	obliquecost	obliquecost	obliquecos	sublinearis	obliquecostata	obliquecostata	obliquecostata	ritscheri	12	1	10	1	0	0	10	0.833	2	0	obliquecostata												

http://apps3.awi.de/Hustedt-Diatoms/FragCl/Set1/L074_W7.0-S8.2-FragCl.ANT33-51.000522.png	73.8	6.8	8.2	NA	ambiguous	obliquecost	sublinearis	sublinearis	sublinearis	obliquecost	obliquecos	sublinearis	ambiguous	obliquecostata	sublinearis	sublinearis	12	0	4	6	2	0	6	0.5	3	0	sublinearis	?sublinearis/ritsc	gradational to F. sublinearis				could be confused with sublineata due to indiscernab			sublinearis or obliquecostata depending on marginal poroids			
http://apps3.awi.de/Hustedt-Diatoms/FragCl/Set1/L074_W7.0-SNA-FragCl.NBP-1402.960-961cm.000042.png	74.0	7.4	NA	6.9	obliquecost	obliquecost	ritscheri	obliquecost	obliquecost	obliquecost	obliquecos	sublinearis	obliquecostata	obliquecostata	obliquecostata	ritscheri	12	2	9	1	0	0	9	0.75	2	0	obliquecostata			tilted specimen but seems heteropolar to me									
http://apps3.awi.de/Hustedt-Diatoms/FragCl/Set1/L074_W8.0-S6.4-FragCl.ANT33-51.000165.png	74.1	7.8	6.4	6.2	obliquecost	obliquecost	ritscheri	obliquecost	obliquecost	obliquecost	obliquecos	obliquecostata	obliquecostata	obliquecostata	obliquecostata	ritscheri	12	2	10	0	0	0	10	0.833	2	0	obliquecostata												
http://apps3.awi.de/Hustedt-Diatoms/FragCl/Set1/L074_W8.0-S5.0-FragCl.ANT33-76.000047.png	74.4	8.4	5.0	NA	ambiguous	obliquecost	obliquecost	obliquecost	obliquecost	obliquecost	obliquecos	obliquecostata	obliquecostata	obliquecostata	obliquecostata	obliquecostata	12	0	11	0	1	0	11	0.916	2	0	obliquecostata	?kerguelensis/obliquecostata											
http://apps3.awi.de/Hustedt-Diatoms/FragCl/Set1/L075_W8.0-S7.0-FragCl.NBP-1402.960-961cm.000103.png	74.6	7.8	7.0	NA	obliquecost	obliquecost	ritscheri	ritscheri	ritscheri	ritscheri	ritscheri	obliquecostata	ambiguous	sublinearis	obliquecostata	ritscheri	12	6	4	1	1	0	6	0.5	1	0	ritscheri		gradational to F. ritscheri					not 100% sure could also be oblique		sublinearis or obliquecostata depending on marginal poroids			
http://apps3.awi.de/Hustedt-Diatoms/FragCl/Set1/L075_W10.0-S7.7-FragCl.ANT33-51.000183.png	74.7	9.7	7.7	6.9	obliquecost	obliquecost	ritscheri	obliquecost	ritscheri	obliquecost	obliquecos	obliquecostata	ambiguous	obliquecostata	obliquecostata	ritscheri	12	3	8	0	1	0	8	0.666	2	0	obliquecostata		gradational to F. ritscheri							sublinearis or obliquecostata depending on marginal poroids			
http://apps3.awi.de/Hustedt-Diatoms/FragCl/Set1/L075_W8.0-S7.1-FragCl.ANT33-76.000054.png	74.9	7.7	7.1	NA	ambiguous	obliquecost	ritscheri	obliquecost	obliquecost	obliquecost	obliquecos	sublinearis	ambiguous	obliquecostata	obliquecostata	ritscheri	12	2	7	1	2	0	7	0.583	2	0	obliquecostata	?sublinearis/oblic	gradational to F. ritscheri							sublinearis or obliquecostata depending on marginal poroids			
http://apps3.awi.de/Hustedt-Diatoms/FragCl/Set1/L075_W8.0-S8.4-FragCl.ANT33-76.000048.png	75.0	8.5	8.4	NA	obliquecost	obliquecost	ritscheri	obliquecost	obliquecost	obliquecost	obliquecos	obliquecostata	ambiguous	obliquecostata	obliquecostata	ritscheri	12	2	9	0	1	0	9	0.75	2	0	obliquecostata									sublinearis or obliquecostata depending on marginal poroids			
http://apps3.awi.de/Hustedt-Diatoms/FragCl/Set1/L075_W8.0-S5.8-FragCl.NBP-1402.960-961cm.000082.png	75.1	8.1	5.8	NA	ambiguous	ritscheri	ritscheri	ritscheri	ritscheri	obliquecost	ritscheri	obliquecostata	ritscheri	obliquecostata	obliquecostata	ritscheri	12	7	4	0	1	0	7	0.583	1	0	ritscheri	?ritscheri/oblique	gradational to F. obliquecostata				could be confuse	not 100% sure could also be obliquecostata					
http://apps3.awi.de/Hustedt-Diatoms/FragCl/Set1/L075_W6.0-SNA-FragCl.ANT33-100.000079.png	75.3	6.1	NA	9.1	sublinearis	obliquecost	sublinearis	sublinearis	obliquecost	sublinearis	sublinearis	sublinearis	ambiguous	sublinearis	sublinearis	sublinearis	12	0	2	9	1	0	9	0.75	3	0	sublinearis		gradational to F. s	don't see much th	Atypical shape?	possibly sublinea	could be confuse	not sure could also be obliquecosta		sublinearis or obliquecostata depending on marginal poroids			
http://apps3.awi.de/Hustedt-Diatoms/FragCl/Set1/L076_W9.0-S8.1-FragCl.NBP-1402.999-996cm.000016.png	75.7	8.5	8.1	NA	ambiguous	ritscheri	ritscheri	ambiguous	ritscheri	ritscheri		obliquecostata	ritscheri	obliquecostata	ambiguous	ritscheri	11	6	2	0	3	0	6	0.545	1	0	ritscheri	?ritscheri/obliquecostata			ritscheri/obliqueo	heteropolar						obliquecostata?: specimen out of fo	
http://apps3.awi.de/Hustedt-Diatoms/FragCl/Set1/L076_W8.0-S5.7-FragCl.ANT33-76.000051.png	75.8	7.7	5.7	NA	ambiguous	obliquecost	ritscheri	obliquecost	obliquecost	obliquecostata		sublinearis	obliquecostata	obliquecostata	obliquecostata	ritscheri	11	2	7	1	1	0	7	0.636	2	0	obliquecostata	?kerguelensis/obliquecostata		heteropolar, strong ornementation looks more like obliquecostata									
http://apps3.awi.de/Hustedt-Diatoms/FragCl/Set1/L076_W8.0-S5.9-PS1768-8.000460.png	75.9	7.7	5.9	NA	ambiguous	obliquecost	sublinearis	obliquecost	obliquecost	obliquecostata		sublinearis	ambiguous	obliquecostata	sublinearis	sublinearis	11	0	5	4	2	0	5	0.454	2	0	obliquecostata	?sublinearis/obliquecostata		based on presence of keel punctae but need more foc			could be confused with sublineata due to indiscernab			sublinearis or obliquecostata depending on marginal poroids			
http://apps3.awi.de/Hustedt-Diatoms/FragCl/Set1/L076_W9.0-S6.3-FragCl.ANT33-51.000121.png	76.0	9.3	6.3	6.9	obliquecost	obliquecost	ritscheri	obliquecost	obliquecost	obliquecostata		obliquecostata	obliquecostata	obliquecostata	obliquecostata	ritscheri	11	2	9	0	0	0	9	0.818	2	0	obliquecostata												
http://apps3.awi.de/Hustedt-Diatoms/FragCl/Set1/L076_W10.0-S7.6-FragCl.ANT33-51.000152.png	76.1	9.7	7.6	7.1	obliquecost	obliquecost	ritscheri	obliquecost	obliquecost	obliquecost	obliquecos	obliquecostata	obliquecostata	obliquecostata	ritscheri	obliquecostata	12	2	10	0	0	0	10	0.833	2	0	obliquecostata									possibly sublinearis			
http://apps3.awi.de/Hustedt-Diatoms/FragCl/Set1/L076_W8.0-S7.5-PS1768-8.000492.png	76.2	8.3	7.5	NA	obliquecost	obliquecost	ritscheri	obliquecost	obliquecost	obliquecostata		obliquecostata	ambiguous	obliquecostata	obliquecostata	sublinearis	11	1	8	1	1	0	8	0.727	2	0	obliquecostata									sublinearis or obliquecostata depending on marginal poroids			
http://apps3.awi.de/Hustedt-Diatoms/FragCl/Set1/L076_W7.0-S5.5-PS1768-8.000853.png	76.3	7.4	5.5	NA	ambiguous	obliquecost	obliquecost	obliquecost	obliquecost	obliquecostata		sublinearis	obliquecostata	obliquecostata	obliquecostata	obliquecostata	11	0	9	1	1	0	9	0.818	2	0	obliquecostata	?sublinearis/obliquecostata											
http://apps3.awi.de/Hustedt-Diatoms/FragCl/Set1/L076_W9.0-S7.0-FragCl.NBP-1402.999-996cm.000009.png	76.4	9.0	7.0	NA	ambiguous	ritscheri	ritscheri	ritscheri	ritscheri	ritscheri		obliquecostata	ritscheri	ritscheri	ritscheri	ritscheri	11	9	1	0	1	0	9	0.818	1	0	ritscheri	?ritscheri/obliquecostata											
http://apps3.awi.de/Hustedt-Diatoms/FragCl/Set1/L076_W9.0-S7.4-FragCl.ANT33-51.000114.png	76.4	9.3	7.4	7.0	obliquecost	obliquecost	ritscheri	obliquecost	obliquecost	obliquecostata		obliquecostata	ambiguous	obliquecostata	obliquecostata	ritscheri	11	2	8	0	1	0	8	0.727	2	0	obliquecostata									sublinearis or obliquecostata depending on marginal poroids			
http://apps3.awi.de/Hustedt-Diatoms/FragCl/Set1/L076_W9.0-S8.1-FragCl.ANT33-51.000129.png	76.5	9.0	8.1	7.3	obliquecost	obliquecost	ritscheri	obliquecost	obliquecost	obliquecostata		obliquecostata	ambiguous	obliquecostata	obliquecostata	ritscheri	11	2	8	0	1	0	8	0.727	2	0	obliquecostata									sublinearis or obliquecostata depending on marginal poroids			
http://apps3.awi.de/Hustedt-Diatoms/FragCl/Set1/L077_W9.0-S6.8-PS1768-8.000469.png	76.6	9.1	6.8	NA	obliquecost	ritscheri	ritscheri	ritscheri	ritscheri	obliquecostata		obliquecostata	ritscheri	sublinearis	obliquecostata	ritscheri	11	6	4	1	0	0	6	0.545	1	0	ritscheri												
http://apps3.awi.de/Hustedt-Diatoms/FragCl/Set1/L077_W9.0-S5.7-PS1768-8.000496.png	77.3	8.7	5.7	NA	obliquecost	obliquecost	obliquecost	obliquecost	obliquecost	ritscheri		obliquecostata	obliquecostata	obliquecostata	obliquecostata	obliquecostata	11	1	10	0	0	0	10	0.909	2	0	obliquecostata												
http://apps3.awi.de/Hustedt-Diatoms/FragCl/Set1/L077_W10.0-S7.8-FragCl.ANT33-51.000146.png	77.5	10.1	7.8	7.6	obliquecost	obliquecost	ritscheri	obliquecost	obliquecost	obliquecostata		obliquecostata	ambiguous	obliquecostata	obliquecostata	ritscheri	11	2	8	0	1	0	8	0.727	2	0	obliquecostata									sublinearis or obliquecostata depending on marginal poroids			
http://apps3.awi.de/Hustedt-Diatoms/FragCl/Set1/L078_W8.0-S5.5-PS1768-8.000445.png	77.5	7.8	5.5	NA	ambiguous	obliquecost	obliquecost	obliquecost	obliquecost	obliquecostata		obliquecostata	obliquecostata	obliquecostata	obliquecostata	obliquecostata	11	0	10	0	1	0	10	0.909	2	0	obliquecostata	?sublinearis/obliquecostata											
http://apps3.awi.de/Hustedt-Diatoms/FragCl/Set1/L078_W9.0-S5.8-FragCl.NBP-1402.960-961cm.000089.png	77.6	9.0	5.8	NA	ritscheri	ritscheri	ritscheri	ritscheri	ritscheri	ritscheri		obliquecostata	ritscheri	obliquecostata	obliquecostata	ritscheri	11	8	3	0	0	0	8	0.727	1	0	ritscheri						could be confused with obliquecostata due to slightly oblique costae BUT heteropolarity too pointed one end						
http://apps3.awi.de/Hustedt-Diatoms/FragCl/Set1/L079_W9.0-SNA-FragCl.NBP-1402.945-946cm.000067.png	79.2	8.8	NA	7.3	obliquecost	ritscheri	ritscheri	ritscheri	ritscheri	ritscheri		obliquecostata	ritscheri	obliquecostata	ritscheri	ritscheri	11	8	3	0	0	0	8	0.727	1	0	ritscheri												
http://apps3.awi.de/Hustedt-Diatoms/FragCl/Set1/L079_W9.0-S7.4-FragCl.ANT33-51.000382.png	79.3	9.2	7.4	NA	obliquecost	obliquecost	ritscheri	obliquecost	obliquecost	obliquecostata		obliquecostata	obliquecostata	obliquecostata	obliquecostata	ritscheri	11	2	9	0	0	0	9	0.818	2	0	obliquecostata		gradational to F. ritscheri							sublinearis if marginal poroids were more distinct on both sides			
http://apps3.awi.de/Hustedt-Diatoms/FragCl/Set1/L079_W9.0-S6.9-FragCl.ANT33-51.000347.png	79.3	9.2	6.9	NA	obliquecost	obliquecost	ritscheri	obliquecost	obliquecost	obliquecostata		obliquecostata	obliquecostata	obliquecostata	obliquecostata	ritscheri	11	2	9	0	0	0	9	0.818	2	0	obliquecostata		gradational to F. ritscheri							sublinearis if marginal poroids were more distinct on both sides			
http://apps3.awi.de/Hustedt-Diatoms/FragCl/Set1/L079_W8.0-S6.0-PS1768-8.000964.png	79.4	7.9	6.0	NA	ambiguous	ambiguous	obliquecost	obliquecost	sublinearis	obliquecostata		obliquecostata	ambiguous	obliquecostata	sublinearis	obliquecostata	11	0	6	2	3	0	6	0.545	2	0	obliquecostata	?obliquecostata/l	kerguelensis-ish: just so coarse							obliquecostata or sublinearis?			
http://apps3.awi.de/Hustedt-Diatoms/FragCl/Set1/L080_W7.0-S6.1-PS1768-8.000442.png	79.5	7.4	6.1	NA	ambiguous	obliquecost	obliquecost	obliquecost	sublinearis	obliquecost	obliquecos	peragallii	obliquecostata	obliquecostata	sublinearis	obliquecostata	12	0	8	2	1	1	8	0.666	2	0	obliquecostata	?sublinearis/obliquecostata								sublinearis if marginal poroids were more distinct on both sides			
http://apps3.awi.de/Hustedt-Diatoms/FragCl/Set1/L080_W10.0-S7.0-PS1768-8.000463.png	79.7	10.3	7.0	NA	obliquecost	ritscheri	ritscheri	ritscheri	ritscheri	obliquecost	ritscheri	obliquecostata	ritscheri	obliquecostata	ritscheri	ritscheri	12	8	4	0	0	0	8	0.666	1	0	ritscheri						could be confuse	size like obliquecostata, shape like ritscheri					
http://apps3.awi.de/Hustedt-Diatoms/FragCl/Set1/L080_W8.0-S7.4-FragCl.NBP-1402.960-961cm.000094.png	80.1	8.5	7.4	NA	ritscheri	ritscheri	ritscheri	ritscheri	ritscheri	ritscheri	ritscheri	obliquecostata	ambiguous	obliquecostata	ritscheri	ritscheri	12	9	2	0	1	0	9	0.75	1	0	ritscheri							size like obliquecostata, shape like r		ritscheri or sublinearis			
http://apps3.awi.de/Hustedt-Diatoms/FragCl/Set1/L082_W10.0-S6.0-PS1768-8.000454.png	81.6	9.6	6.0	NA	obliquecost	ambiguous	obliquecost	obliquecost	sublinearis	kerguelensi	obliquecos	obliquecostata	ambiguous	obliquecostata	obliquecostata	obliquecostata	12	0	8	1	2	1	8	0.666	2	0	obliquecostata		kerguelensis-ish: just so coarse		Tilted?								
http://apps3.awi.de/Hustedt-Diatoms/FragCl/Set1/L082_W9.0-S7.3-FragCl.ANT33-51.000181.png	81.7	9.4	7.3	7.1	obliquecost	obliquecost	ritscheri	obliquecost	obliquecost	obliquecost	obliquecos	obliquecostata	obliquecostata	obliquecostata	obliquecostata	ritscheri	12	2	10	0	0	0	10	0.833	2	0	obliquecostata												
http://apps3.awi.de/Hustedt-Diatoms/FragCl/Set1/L082_W8.0-S5.6-PS1768-8.000962.png	81.9	7.5	5.6	NA	ambiguous	obliquecost	obliquecost	obliquecost	obliquecost	obliquecost	obliquecos	peragallii	obliquecostata	obliquecostata	obliquecostata	obliquecostata	12	0	10	0	1	1	10	0.833	2	0	obliquecostata	?obliquecostata/kerguelensis											
http://apps3.awi.de/Hustedt-Diatoms/FragCl/Set1/L083_W7.0-S4.9-PS1768-8.000765.png	82.5	6.7	4.9	NA	ambiguous	obliquecost	obliquecost	obliquecost	obliquecost	obliquecost	obliquecos	sublinearis	obliquecostata	obliquecostata	obliquecostata	obliquecostata	12	0	10	1	1	0	10	0.833	2	0	obliquecostata	?sublinearis/kerguelensis											
http://apps3.awi.de/Hustedt-Diatoms/FragCl/Set1/L083_W8.0-S5.5-PS1768-8.000484.png	83.4	8.2	5.5	NA	ambiguous	obliquecost	obliquecost	obliquecost	obliquecost	obliquecost	obliquecos	obliquecostata	obliquecostata	obliquecostata	obliquecostata	obliquecostata	12	0	11	0	1	0	11	0.916	2	0	obliquecostata	?sublinearis/obliquecostata											

http://apps3.awi.de/Hustedt-Diatoms/FragCl/Set1/L083_W7.0-S7.5-FragCl.NBP-1402.960-961cm.000107.png	83.4	7.4	7.5	NA		ambiguous	ambiguous		obliquecost	sublinearis	obliquecost	obliquecos	obliquecostata	obliquecostata		sublinearis	sublinearis	10	0	5	3	2	0	5	0.5	2	0	obliquecostata	?obliquecostata/s	sublinearis - obliq	tilted			could be confused with ritscherii, but not heteropolar enough. Costae may be illusionally oblique, as with transapical width bei						
http://apps3.awi.de/Hustedt-Diatoms/FragCl/Set1/L084_W9.0-S7.9-FragCl.ANT33-51.000205.png	84.1	9.4	7.9	NA		obliquecost	obliquecost	obliquecost	obliquecost	obliquecost	obliquecost	obliquecos	obliquecostata	obliquecostata	obliquecostata	obliquecostata	sublinearis	12	0	11	1	0	0	11	0.916	2	0	obliquecostata									sublinearis if marginal poroids were more distinct on both sides			
http://apps3.awi.de/Hustedt-Diatoms/FragCl/Set1/L086_W8.0-SNA-FragCl.NBP-1402.945-946cm.000066.png	86.1	8.1	NA	7.4		obliquecost	obliquecost	ritscheri	ritscheri	ritscheri	obliquecost	obliquecos	obliquecostata	ambiguous	obliquecostata	obliquecostata	ritscheri	12	4	7	0	1	0	7	0.583	2	0	obliquecostata			but not sure, seems heteropolar			could be ritscherii			sublinearis or obliquecostata depending on marginal poroids			
http://apps3.awi.de/Hustedt-Diatoms/FragCl/Set1/L086_W8.0-S5.7-PS1768-8.000963.png	86.4	8.0	5.7	NA		obliquecost	obliquecost	obliquecost	obliquecost	obliquecost	obliquecost	obliquecos	obliquecostata	obliquecostata	obliquecostata	obliquecostata	obliquecostata	12	0	12	0	0	0	12	1	2	0	obliquecostata												
http://apps3.awi.de/Hustedt-Diatoms/FragCl/Set1/L088_W9.0-S6.7-FragCl.ANT33-51.000526.png	87.7	9.1	6.7	NA		obliquecost	obliquecost	ritscheri	obliquecost	obliquecost	obliquecost	obliquecos	obliquecostata	ambiguous	obliquecostata	obliquecostata	sublinearis	12	1	9	1	1	0	9	0.75	2	0	obliquecostata			largest widht not equidistant from apices (to the lower part)						sublinearis or obliquecostata depending on marginal poroids			
http://apps3.awi.de/Hustedt-Diatoms/FragCl/Set1/L088_W9.0-S5.1-PS1768-8.000777.png	88.2	8.6	5.1	NA		ambiguous	obliquecost	obliquecost	obliquecost	obliquecost	obliquecost	obliquecos	obliquecostata	obliquecostata	obliquecostata	obliquecostata	obliquecostata	12	0	11	0	1	0	11	0.916	2	0	obliquecostata	?obliquecostata/kerguelensis											
http://apps3.awi.de/Hustedt-Diatoms/FragCl/Set1/L089_W8.0-S6.3-PS1768-8.000849.png	89.2	8.1	6.3	NA		obliquecost	obliquecost	obliquecost	obliquecost	obliquecost	obliquecost	obliquecos	obliquecostata	obliquecostata	obliquecostata	obliquecostata	obliquecostata	12	0	12	0	0	0	12	1	2	0	obliquecostata									sublinearis if marginal poroids were more distinct on both sides			
http://apps3.awi.de/Hustedt-Diatoms/FragCl/Set1/L090_W9.0-S6.1-FragCl.ANT33-51.000349.png	90.4	8.5	6.1	NA		obliquecost	obliquecost	obliquecost	obliquecost	obliquecost	obliquecost	obliquecos	obliquecostata	obliquecostata	obliquecostata	obliquecostata	obliquecostata	12	0	12	0	0	0	12	1	2	0	obliquecostata												
http://apps3.awi.de/Hustedt-Diatoms/FragCl/Set1/L090_W9.0-SNA-FragCl.ANT33-51.000171.png	90.5	9.3	NA	8.1		obliquecost	obliquecost	obliquecost	obliquecost	obliquecost	obliquecost	obliquecos	obliquecostata	obliquecostata	obliquecostata	obliquecostata	sublinearis	12	0	11	1	0	0	11	0.916	2	0	obliquecostata												
http://apps3.awi.de/Hustedt-Diatoms/FragCl/Set1/L091_W9.0-S8.9-FragCl.ANT33-100.000106.png	90.6	9.2	8.9	8.4		obliquecost	obliquecost	ritscheri	ritscheri	sublinearis	obliquecost	obliquecos	obliquecostata	ambiguous	obliquecostata	obliquecostata	ritscheri	12	3	7	1	1	0	7	0.583	2	0	obliquecostata				Atypical very long	really not sure - stria are not slanted, heteropolar but both pointy ... not				sublinearis or obliquecostata depending on marginal poroids			
http://apps3.awi.de/Hustedt-Diatoms/FragCl/Set1/L091_W9.0-S7.1-FragCl.ANT33-51.000160.png	90.9	9.4	7.1	6.9		obliquecost	obliquecost	obliquecost	obliquecost	obliquecost	obliquecost	obliquecos	obliquecostata	sublinearis	obliquecostata	obliquecostata	obliquecostata	12	0	11	1	0	0	11	0.916	2	0	obliquecostata									possibly obliquecostata			
http://apps3.awi.de/Hustedt-Diatoms/FragCl/Set1/L092_W8.0-S5.7-PS1768-8.000421.png	91.7	7.8	5.7	NA		obliquecost	obliquecost	obliquecost	obliquecost	obliquecost	obliquecost	obliquecos	obliquecostata	obliquecostata	obliquecostata	obliquecostata	obliquecostata	12	0	12	0	0	0	12	1	2	0	obliquecostata												low silicification
http://apps3.awi.de/Hustedt-Diatoms/FragCl/Set1/L092_W8.0-S7.3-FragCl.ANT33-76.000041.png	91.8	8.0	7.3	NA		obliquecost	obliquecost	obliquecost	obliquecost	obliquecost	obliquecostata		obliquecostata	obliquecostata	obliquecostata	obliquecostata	obliquecostata	11	0	11	0	0	0	11	1	2	0	obliquecostata									sublinearis if marginal poroids were more distinct on both sides			
http://apps3.awi.de/Hustedt-Diatoms/FragCl/Set1/L092_W8.0-S7.3-FragCl.ANT33-51.000350.png	92.1	8.4	7.3	NA		obliquecost	obliquecost	obliquecost	obliquecost	obliquecost	obliquecost	obliquecos	obliquecostata	obliquecostata	obliquecostata	obliquecostata	ritscheri	12	1	11	0	0	0	11	0.916	2	0	obliquecostata									sublinearis if marginal poroids were more distinct on both sides			
http://apps3.awi.de/Hustedt-Diatoms/FragCl/Set1/L094_W9.0-S6.8-FragCl.NBP-1402.960-961cm.000091.png	93.7	8.5	6.8	NA		ritscheri	obliquecost	ritscheri	ritscheri	ritscheri	ritscheri		obliquecostata	sublinearis	obliquecostata	ritscheri	ritscheri	11	7	3	1	0	0	7	0.636	1	0	ritscheri												
http://apps3.awi.de/Hustedt-Diatoms/FragCl/Set1/L094_W9.0-S7.2-FragCl.ANT33-51.000348.png	94.3	8.7	7.2	NA		obliquecost	obliquecost	ritscheri	obliquecost	obliquecost	obliquecostata		obliquecostata	obliquecostata	obliquecostata	obliquecostata	obliquecostata	11	1	10	0	0	0	10	0.909	2	0	obliquecostata									sublinearis if marginal poroids were more distinct on b			low silicification
http://apps3.awi.de/Hustedt-Diatoms/FragCl/Set1/L095_W8.0-S5.2-PS1768-8.000759.png	95.2	7.6	5.2	NA		ambiguous	obliquecost	obliquecost	obliquecost	obliquecost	obliquecostata		obliquecostata	obliquecostata	obliquecostata	obliquecostata	obliquecostata	11	0	10	0	1	0	10	0.909	2	0	obliquecostata	?obliquecostata/ritscherii											
http://apps3.awi.de/Hustedt-Diatoms/FragCl/Set1/L098_W9.0-S7.9-FragCl.ANT33-51.000147.png	98.0	8.8	7.9	8.0		obliquecost	obliquecost	obliquecost	obliquecost	obliquecost	obliquecostata		obliquecostata	ambiguous	obliquecostata	obliquecostata	obliquecostata	11	0	10	0	1	0	10	0.909	2	0	obliquecostata			but not sure, seems heteropolar			with F. curta inside! :-)			sublinearis or obliquecostata depending on marginal p			low silicification
http://apps3.awi.de/Hustedt-Diatoms/FragCl/Set1/L101_W9.0-SNA-FragCl.NBP-1402.945-946cm.000040.png	100.6	9.2	NA	6.7		obliquecost	obliquecost	ritscheri	ritscheri	obliquecost	obliquecost	obliquecos	obliquecostata	ambiguous	obliquecostata	obliquecostata	ritscheri	12	3	8	0	1	0	8	0.666	2	0	obliquecostata				Hard to see					sublinearis or obliquecostata depending on marginal poroids			
http://apps3.awi.de/Hustedt-Diatoms/FragCl/Set1/L101_W9.0-S6.7-FragCl.ANT33-51.000384.png	100.6	8.6	6.7	NA		obliquecost	obliquecost	obliquecost	obliquecost	obliquecost	obliquecost	obliquecos	obliquecostata	ambiguous	obliquecostata	obliquecostata	obliquecostata	12	0	11	0	1	0	11	0.916	2	0	obliquecostata									sublinearis or obliquecostata depending on marginal p			low silicification
http://apps3.awi.de/Hustedt-Diatoms/FragCl/Set1/L102_W9.0-S7.2-FragCl.ANT33-51.000517.png	102.0	8.8	7.2	NA		obliquecost	obliquecost	obliquecost	obliquecost	obliquecost	obliquecost	obliquecos	obliquecostata	obliquecostata	obliquecostata	obliquecostata	obliquecostata	12	0	12	0	0	0	12	1	2	0	obliquecostata									sublinearis if marginal poroids were more distinct on b			low silicification
http://apps3.awi.de/Hustedt-Diatoms/FragCl/Set1/L103_W8.0-S7.4-FragCl.ANT33-76.000064.png	103.1	8.1	7.4	NA		obliquecost	obliquecost	ritscheri	obliquecost	obliquecost	obliquecost	obliquecos	obliquecostata	obliquecostata	obliquecostata	obliquecostata	ritscheri	12	2	10	0	0	0	10	0.833	2	0	obliquecostata									sublinearis if marginal poroids were more distinct on both sides			
http://apps3.awi.de/Hustedt-Diatoms/FragCl/Set1/L118_W9.0-S7.0-FragCl.ANT33-76.000069.png	118.3	9.3	7.0	NA		obliquecost	obliquecost	obliquecost	obliquecost	obliquecost	obliquecost	obliquecos	obliquecostata	obliquecostata	obliquecostata	obliquecostata	obliquecostata	12	0	12	0	0	0	12	1	2	0	obliquecostata									sublinearis if marginal poroids were more distinct on b			low silicification
http://apps3.awi.de/Hustedt-Diatoms/FragCl/Set1/L121_W8.0-S6.4-FragCl.ANT33-76.000071.png	120.5	8.3	6.4	NA		obliquecost	obliquecost	obliquecost	obliquecost	sublinearis	obliquecost	obliquecos	obliquecostata	obliquecostata	obliquecostata	obliquecostata	obliquecostata	12	0	11	1	0	0	11	0.916	2	0	obliquecostata					difficult photo, apparently striaght striae, not slanted				sublinearis if marginal poroids were more distinct on b			low silicification
